# Association between triglyceride-glucose index and sarcopenia: a meta-analysis

**DOI:** 10.7717/peerj.21424

**Published:** 2026-07-23

**Authors:** Shuying Zou, Yuzhou Li, Xiangnan Zhu, Li Tang, Qianqian Zhang, Caixia Xie

**Affiliations:** 1 School of Nursing, Chengdu University of Traditional Chinese Medicine, Chengdu, Sichuan, China; 2 Department of Nursing, Sichuan Provincial People’s Hospital, School of Medicine, University of Electronic Science and Technology of China, Chengdu, China

**Keywords:** Triglyceride-glucose index, Sarcopenia, Meta-analysis, Triglyceride-glucose body mass index

## Abstract

**Background:**

With accelerating population aging, sarcopenia has become increasingly common in the elderly. Because the triglyceride-glucose index is correlated with sarcopenia, this study aims to investigate the association between the triglyceride-glucose index and sarcopenia.

**Methods:**

A systematic search of PubMed, Web of Science, Embase, and Cochrane Library databases was conducted for relevant studies published up to June 15, 2025. Pooled odds ratios (ORs) with 95% confidence intervals (CIs) were calculated using a random-effects model in STATA 15.0.

**Results:**

Analysis of 14 studies (*n* = 82,798) revealed a significant association between the triglyceride- glucose index (TyG index) and sarcopenia (OR = 2.15, 95% CI [1.60–2.89]). A significant correlation was also observed in the TyG quartile groups: Q2 (OR = 1.38, 95% CI [1.08–1.75]), Q3 (OR = 1.64, 95% CI [1.21–2.20]), and Q4 (OR = 1.75, 95% CI [1.21–2.55]). The TyG-BMI index demonstrated a stronger association, with the strength of this association increasing significantly as the quartile level rose Q2 (OR = 2.51, 95% CI [1.55–4.05]); Q3 (OR = 5.01, 95% CI [1.89–13.25]); Q4 (OR = 9.08, 95% CI [2.91–28.37]). Subgroup analyses revealed that the Asian Working Group for Sarcopenia 2019 (AWGS 2019) diagnostic criteria (OR = 5.28, 95% CI [2.27–12.3]), middle-aged individuals (OR = 1.87, 95% CI [1.46–2.42]), individuals with comorbidities (OR = 1.94, 95% CI [1.54–2.44]), and different TyG measurement methods (OR = 2.14, 95% CI [1.24–3.71]) were also significantly associated with sarcopenia.

**Conclusions:**

This meta-analysis confirms a significant association between the TyG index and sarcopenia. The prevalence of sarcopenia increases with the elevation of the TyG index. This highlights the potential value of the TyG index as a key indicator for sarcopenia screening and early intervention. Trial registration:CRD420251067364, June 25, 2025.

## Background

Sarcopenia is a progressive musculoskeletal condition defined by a loss of muscle mass, strength, and physical performance. Following its onset, patients typically experience a marked reduction in muscular strength, ultimately leading to restricted mobility and a significant deterioration in quality of life (Ding et al., 2024). Key adverse outcomes encompass heightened risks of fragility fractures, falls, injuries, disability, and reduced physical capacity. Moreover, the condition can threaten the independent living of older adults (Chen et al., 2020; Cruz-Jentoft et al., 2019). Reduced muscle mass and strength are associated with worse outcomes from mechanical ventilation and longer hospital stays (Cruz-Jentoft & Sayer, 2019). Epidemiological data show that sarcopenia affects at least 4.6% of older adults and rises to about 25% in elderly inpatients (Ahn, Lee & Lee, 2020). Muscle loss is especially pronounced after age 70, with annual loss rates of 0.5% to 1.0% (Chen et al., 2021). As a result, sarcopenia is widely seen as a major public health issue.

The triglyceride-glucose (TyG) index is a recently established marker of metabolic function and a practical surrogate for insulin resistance. This index is calculated by combining the values of fasting plasma glucose and triglyceride concentrations. Compared with the hyperinsulinemic-euglycemic clamp technique—the reference method for quantifying insulin resistance (IR)—the TyG index provides distinct practical benefits: it is inexpensive, easy to implement, and demonstrates high reproducibility. Its calculation relies solely on routine laboratory tests using a straightforward formula (Marcovina et al., 2007). Recently, the TyG index has been recognized as a robust predictor of metabolic disease incidence. Owing to its strong correlation with insulin resistance (IR), the TyG index is useful for assessing several metabolic disorders, including metabolic syndrome, type 2 diabetes, and metabolic dysfunction-associated steatotic liver disease (MASLD) (Dong et al., 2026; Hasanato et al., 2026; Mondal et al., 2026). Additionally, accumulating evidence supports the TyG index as a reliable marker for a range of cardiovascular conditions, including heart failure, acute coronary syndrome, hypertrophic cardiomyopathy, atherosclerosis, atrial fibrillation, and cardio-renal-metabolic syndrome (Bampali et al., 2026; Hasan et al., 2026; Li et al., 2026; Li et al., 2025b; Wang et al., 2026a; Wang et al., 2026b). Sarcopenia is strongly linked to chronic low-grade inflammation (CLIP). Older individuals with CLIP often show reductions in muscle mass and strength. Moreover, inflammatory processes exacerbate IR, potentially elevating the TyG index (Beyer, Mets & Bautmans, 2012). One study suggests that proinflammatory mediators contribute significantly to tissue-specific IR by disrupting insulin signaling pathways (Rehman & Akash, 2016). Observational studies have confirmed a direct association between sarcopenia and IR (Zhao et al., 2025a). In this context, the TyG index serves as a convenient proxy for IR. A cross-sectional study documented a significant positive association between the TyG index and skeletal muscle mass index in Korean adults (Ahn, Lee & Lee, 2020). In contrast, a nationwide cohort study and Mendelian randomization analysis revealed an inverse relationship between the TyG index and both grip strength and walking speed (Page et al., 2021). This apparent inconsistency in the direction of associations arises from the diverse diagnostic dimensions of sarcopenia. Sarcopenia is diagnosed based on assessments of low muscle mass, reduced muscle strength, and impaired physical performance, according to major international criteria. The observed inconsistency may also stem from differences in study populations, analytical designs, and outcome assessments across individual investigations. Conflicting findings from single studies have fueled ongoing debate regarding the overall association between the TyG index and sarcopenia. This underscores the need for a comprehensive meta-analysis to synthesize existing evidence and draw a unified quantitative conclusion. Thus, this meta-analysis aims to evaluate the relationship between the TyG index and sarcopenia and to provide quantitative evidence on their association.

## Materials and Methods

The present meta-analysis was conducted in accordance with the Preferred Reporting Items for Systematic Reviews and Meta-Analyses (PRISMA 2020) guidelines (Page et al., 2021). The study protocol has been registered with the International Prospective Register of Systematic Reviews (PROSPERO) under the registration number CRD420251067364.

### Literature search and selection

The search strategy was collaboratively developed by two researchers (Shuying Zou and Yuzhou Li). Retrieved records were screened against predefined inclusion and exclusion criteria using EndNote X9 (Clarivate). Duplicate records were identified and removed in EndNote X9 prior to the screening process. A comprehensive search strategy was designed across PubMed, Embase, Web of Science, and the Cochrane Library databases, up to June 15, 2025, using a combination of Medical Subject Headings (MeSH) terms and free-text keywords. Key search terms included “sarcopenia”, “Triglyceride-Glucose Index”, “sarcopenias”, and “TyG index”. Two researchers (Shuying Zou and Yuzhou Li) independently screened titles and abstracts for relevance. Subsequently, potentially eligible articles were assessed in full-text independently by the same two reviewers for final inclusion. Any disagreements during the selection process were resolved through discussion and consensus, with a third researcher (Caixia Xie) available to arbitrate if necessary. The complete search strategy is presented in Supplementary Materials. The initial systematic literature search was conducted up to June 15, 2025. To ensure the inclusion of studies published between the completion of the initial search and manuscript submission, a first updated search was performed on January 21, 2026, covering the period from June 16, 2025, to January 3, 2026 (the initial submission date). For records identified in this first updated search, titles, abstracts, and full texts were independently screened against the eligibility criteria by two authors (Shuying Zou and Yuzhou Li) on January 21, 2026. No new eligible studies were identified through this updated search; therefore, data extraction for new studies was not applicable. Any disagreements during the screening process were resolved by consensus or, if necessary, through consultation with a third author (Caixia Xie). Given the time elapsed since the previous search and to ensure the timeliness of our systematic review, we performed a second updated search on April 23, 2026, covering the period from January 4, 2026, to April 23, 2026, to avoid missing any recently published studies that might affect the conclusions of our meta-analysis. The same screening process was applied, and no new eligible studies were identified.

### Study selection

Studies were considered eligible if they fulfilled all of the following criteria: 1. Published in peer-reviewed journals; 2. Employed a cross-sectional design; 3. Defined sarcopenia using both low muscle mass and low muscle strength or impaired physical function, consistent with major international diagnostic criteria such as AWGS 2019, AWGS 2014 (Chen et al., 2020), FNIH (Studenski et al., 2014), or EWGSOP 2010 (Cruz-Jentoft et al., 2010); 4. Examined the association between either the TyG index or the TyG-BMI index and sarcopenia; 5. Provided data enabling the calculation of pooled odds ratios (ORs) and corresponding 95% confidence intervals (CIs) according to TyG index quartiles (Q2: 2.90–3.39; Q3: 3.40–8.74; Q4: ≥8.75) and TyG-BMI index quartiles (Q2: 196–235; Q3: 236–282; Q4: ≥283). Articles were excluded if they: 1. Non-human studies, including animal experiments; 2. Letters, conference abstracts, editorials, or narrative reviews; 3. Unavailability of essential raw data; 4. Duplicate studies published within the same original publication or within the database.

### Data extraction

The study screening and data collection were conducted independently by two evaluators (Shuying Zou and Xiangnan Zhu). Any disagreements in this process were resolved by consensus following team discussions. A standardized Excel spreadsheet was used to collect the following variables: first author, year of publication, country, sample size, participant age and sex, comorbidities, the diagnostic criteria for sarcopenia, and the statistical model employed. Where data were incomplete, we attempted to contact the first or corresponding authors to acquire the necessary information.

### Quality assessment

The methodological quality of the included studies was independently evaluated by two reviewers trained in evidence-based research. Accordingly, the assessment employed the risk-of-bias tool developed by the Agency for Healthcare Research and Quality (AHRQ). Discrepancies between reviewers were resolved through consultation with a third investigator (Caixia Xie). This tool comprises 11 items, each scored as “yes” (1 point) or “no/unclear” (0 points). Total scores were interpreted as follows: 0–3, low quality; 4–7, moderate quality; 8–11, high quality.

### Data synthesis and statistical analyses

Statistical analyses were conducted with STATA software (version 15.0). The primary outcome measures were the associations of the TyG index and TyG-BMI index with sarcopenia, expressed as pooled odds ratios (ORs) with 95% confidence intervals (CIs). Between-study heterogeneity was quantified using the Q test and *I*^2^ statistic. Considering the inherent clinical and methodological heterogeneity among observational studies, we pre-specified the use of a random-effects model for all primary meta-analyses to provide more conservative pooled estimates. Subgroup analyses and meta-regression were conducted to identify sources of heterogeneity. The studies were stratified according to study population, sarcopenia diagnostic criteria, comorbidities, and TyG index measurement methods. To examine result stability, a sensitivity analysis was performed using a stepwise exclusion method, recalculating the pooled effect size after sequentially excluding one study at a time. Potential publication bias was assessed through funnel plots and Egger’s test. If funnel plots exhibited asymmetry, a trimming procedure was applied to further evaluate the robustness of the findings. Statistical significance was defined as a *P* value <0.05.

## Results

### Study selection

**Figure 1:**
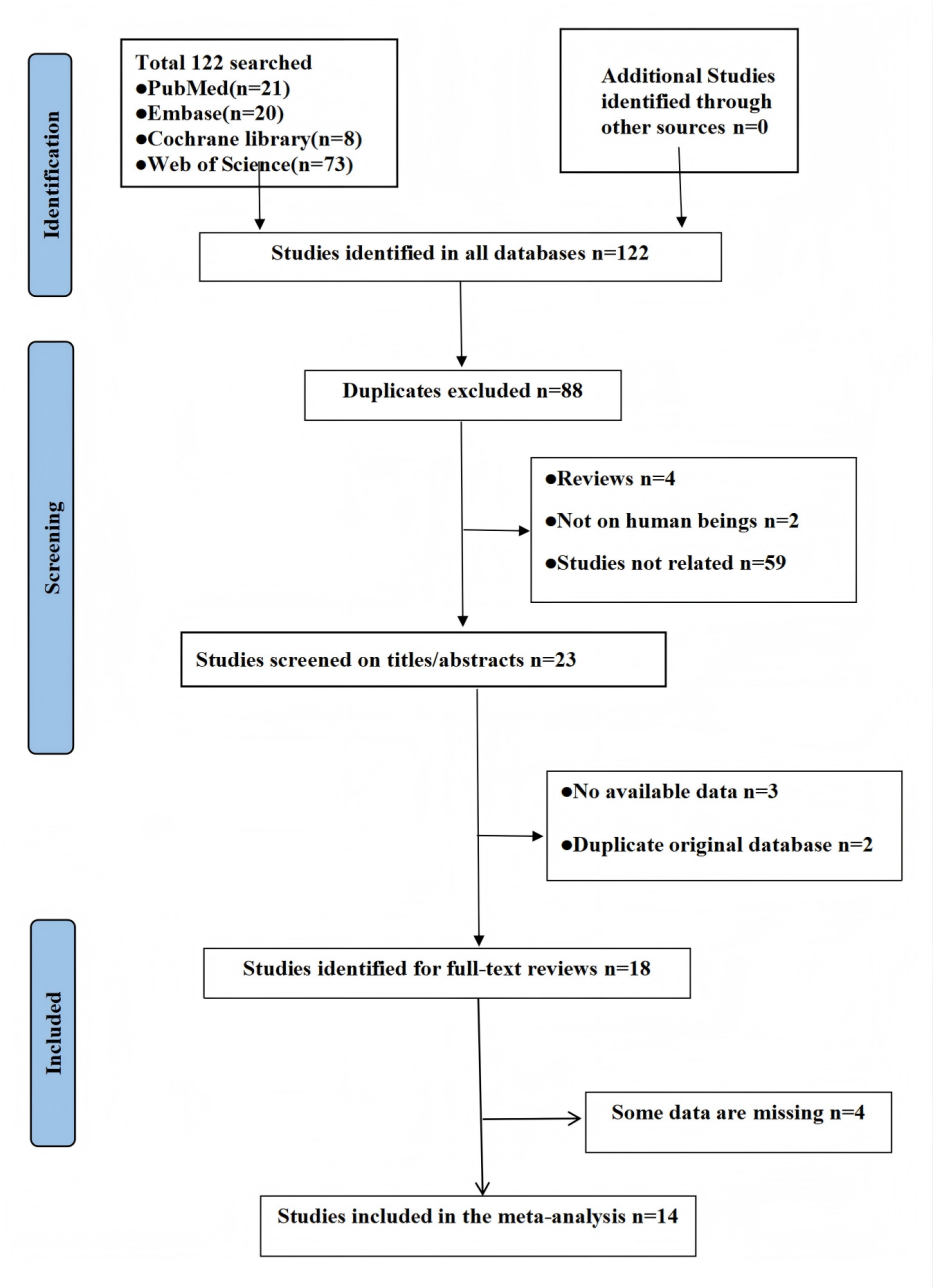
Literature search flowchart.

A preliminary literature search identified 122 records, which were reduced to 88 after removing duplicates. Following the screening of titles and abstracts, 23 articles remained. Upon reviewing the full texts, five articles were excluded due to incomplete data. Of the remaining 18 articles, two were excluded because the data could not be extracted, and another two were excluded due to duplication in the original database. Consequently, 14 studies met all eligibility criteria and were included in the final meta-analysis. A flowchart illustrating the study selection process and results is presented in Fig. 1.

### Study and patient characteristics

This meta-analysis included 14 cross-sectional studies (Chen et al., 2022; Chen, Liu & Hu, 2024; Hu, Ma & Xing, 2023; Kim et al., 2021; Li et al., 2025a; Li et al., 2024; Li et al., 2026; Wei & Liu, 2024; Yang et al., 2024a; Yang et al., 2024b; Yu et al., 2025; Zhang, Chen & Jiang, 2024; Zhao et al., 2024; Zhao, Ji & Wang, 2025), involving a total of 82,798 participants. The average age of participants was 52.18 years, and 52% of the sample being male. All studies were geographically based in Asia. With the exception of the study by Kim et al (2021), which originated in South Korea; the remaining studies were conducted in China. Regarding sarcopenia diagnostic criteria, eight studies (Chen et al., 2022; Li et al., 2025a; Li et al., 2024; Pan et al., 2024; Yang et al., 2024a; Yang et al., 2024b; Yu et al., 2025; Zhao, Ji & Wang, 2025) used the AWGS criteria, one study (Hu, Ma & Xing, 2023) applied the 2010 EWGSOP criteria, and four studies (Chen, Liu & Hu, 2024; Wei & Liu, 2024; Zhang, Chen & Jiang, 2024; Zhao et al., 2024) employed non-international diagnostic standards. Additionally, one study (Kim et al., 2021) adopted the FNIH diagnostic criteria. Detailed characteristics are presented in Table 1.

**Table 1:** Main characteristics of studies included in the systematic review.

Study	Year	Country	Study design	Sample size	Gender (M/F)	Mean age	Definition of sarcopenia	Complicating disease	Regression model
Wenchao Hu	2023	China	Cross-sectional	1,098	588/510	58.58	(EWGSOP 2010): SMI < 7.0 kg/m^2^ (men) SMI < 5.4 kg/m^2^ (women)	A	Multiple linear regression
Jiju Yang	2024	China	Cross-sectional	4,030	2,012/2,018	39.39	(AWGS 2019): ASM/BMI < 0.789 (men) and <0.512 (women)	A; B	Weighted multivariate logistic regression
Min Li	2025	China	Cross-sectional	460	/	64	(AWGS 2019): RSMI: (women) < 5.4 kg/m^2^; HS (women) < 18 kg; 6 m walking test <1 m/s	/	Binary logistic regression
Ruirong Pan	2024	China	Cross-sectional	4,835	2,388/2,447	39.53	(AWGS 2019): ASM/BMI < 0.512 (female) and <0.789 (male)	B	Multivariate logistic regression
Xinyu Yu	2025	China	Cross-sectional	792	351/441	71.71	(AWGS 2014): Handgrip strength < 28.0 kg (males) and <18.0 kg (females); SMI <7.0 kg/m^2^ (males) and <5.7 kg/m^2^ (females)	A; B	Multivariate logistic regression
Xue Wei	2024	China	Cross-sectional	2,504	1,308/1,196	39.27	LMM <0.289 (men) And <0.224 (women); LMS <0.897 (men) and 0.637 (women)	A; B; C	Multifactorial regression
Yong Chen	2024	China	Cross-sectional	1,819	942/877	66.5	(ASM/ height^2^)/ (SMI) <4.950 kg/m^2^ (women) and <6.797 kg/m^2^ (men); five chair stand tests taking ≥ 12s or a gait speed <1.0 m/s	A; B; E; F; G; H	Multivariable-adjusted Cox regression
Zihao Zhang	2024	China	Cross-sectional	10,537	4,845/5,692	59.34	low grip strength <18 kg (women) and <28 kg (men); ASM/Ht2 <7.08 kg/m^2^ (men) and ASM/Ht^2^ <5.43 kg/m^2^ (women)	B; E	Multiple linear regression
Runtao Zhao	2025	China	Cross-sectional	7,161	3,684/3,477	41.77	(AWGS 2019): ASM/BMI <0.789 (men) and <0.512 (women)	A; B	Ordinal logistic regression
Ruoxin Chen	2022	China	Cross-sectional	142	75/67	54.05	(AWGS 2014): handgrip strength <28 kg (men) and <18 kg (women); SMI <7.0 kg/m^2^ (men) and <5.7 kg/m^2^ (women); the 6-meter walking test <1.0 m/s	/	Multivariate logistic regression
Zhenzhen Li	2024	China	Cross-sectional	36,275	21,206/ 15,069	43.74	(AWGS 2014): ASMI <5.7 kg/m^2^ (women) and 7.0 kg/m^2^ (men)	A; B	Multiple logistic regression
JungA. Kim	2021	Korea	Cross-sectional	9,477	3,988/5,479	56.7	(FNIH): ALM/weight <2 standard deviation (SD) <the mean of a young reference group (age 20–39 years), <28.8% (men) and 22.8% (women)	B	Multivariate logistic regression
Qinying Zhao	2024	China	Cross-sectional	2,687	1,316/1,371	57.8	/	A; B	Multiple regression
Xinping Yang	2024	China	Cross-sectional	981	452/529	38.12	(AWGS 2019): ASM/BMI ratio <0.789 (men) and <0.512 (women)	A; B; D	Logistic regression

**Notes.**

Complicating disease: A: Type 2 diabetes mellitus or diabetes; B: hypertension; C: Cardiovascular Disease; D: chronic inflammatory airway disease; E: Heart disease; F: Kidney disease; G: Digestive disease; H: Arthritis or rheumatis.

Note: Citations for studies (Chen et al., 2022; Chen, Liu & Hu, 2024; Hu, Ma & Xing, 2023; Kim et al., 2021; Li et al., 2025a; Li et al., 2024; Li et al., 2026; Wei & Liu, 2024; Yang et al., 2024a; Yang et al., 2024b; Yu et al., 2025; Zhang, Chen & Jiang, 2024; Zhao et al., 2024; Zhao, Ji & Wang, 2025).

### Study quality

All 14 studies included in our analysis employed a cross-sectional design. Methodological quality was assessed using the AHRQ instrument (Agency for Healthcare Research and Quality). Based on the scoring system, studies were classified as low quality (score ≤ 3), moderate quality (score 4–7), or high quality (score ≥ 8). Among the 14 studies, all were rated as either moderate or high quality, with a mean AHRQ score of 6, indicating overall moderate methodological quality (Table 2).

**Table 2:** The quality assessment of the included studies.

Cross-sectional											
Study	Is the source of the data identified (survey, literature review)?	Are the inclusion and exclusion criteria for the exposed and non - exposed groups (cases and controls) listed, or is reference made to previous publications?	Has the time period for identifying patients been given?	If it’s not related to the source of the population, are the research subjects continuous?	Do the subjective factors of the evaluator obscure other aspects of the research object?	Describes any assessments carried out to ensure quality (such as the testing/ re - testing of primary outcome indicators)	Explained the reasons for excluding any patients from the analysis	Describes the measures for evaluating and/or controlling confounding factors	If possible, explain how missing data was handled in the analysis	The response rate of patients and the integrity of data collection were summarized	If there is follow - up, find out the percentage of expected incomplete patient data or the follow - up results.
Min Li 2025	Yes	Yes	Yes	Yes	Yes	Yes	Yes	Yes	No	Unclear	Unclear
Wenchao Hu 2023	Yes	Yes	Yes	Yes	Yes	No	Unclear	Yes	No	Unclear	Unclear
Jiju Yang 2024	Yes	Yes	Yes	Unclear	Yes	Unclear	Yes	Yes	Yes	Yes	Unclear
JungA.Kim 2021	Yes	Yes	Yes	Unclear	Yes	Unclear	Yes	Yes	No	No	Unclear
Qinying Zhao 2024	Yes	Yes	Yes	Unclear	Unclear	No	Yes	Yes	No	No	Unclear
Ruirong Pan 2024	Yes	Yes	Yes	Unclear	Yes	Yes	Yes	Yes	Yes	Unclear	Unclear
Runtao Zhao 2025	Yes	Yes	No	No	Yes	Unclear	Unclear	Yes	Yes	Unclear	Unclear
Ruoxin Chen 2022	Yes	Yes	No	No	Yes	Yes	No	Yes	Unclear	Unclear	Unclear
Xinping Yang 2024	Yes	Yes	Yes	No	Yes	Unclear	Yes	Yes	Unclear	Unclear	Unclear
Xinyu Yu 2025	Yes	Yes	Unclear	Unclear	Unclear	Yes	No	Yes	No	No	Unclear
Xue Wei 2024	Yes	Yes	Yes	Unclear	Unclear	Yes	Yes	Yes	Yes	Yes	Unclear
Yong Chen 2024	Yes	Yes	Yes	Yes	Unclear	No	Yes	Yes	Yes	Yes	Yes
Zhenzhen Li 2024	Yes	Yes	Yes	Unclear	Unclear	No	Yes	Yes	No	Unclear	Unclear
Zihao Zhang 2024	Yes	Yes	No	Unclear	Unclear	Yes	Yes	Yes	No	Unclear	Unclear

**Notes.**

Note: Citations for (Chen et al., 2022; Chen, Liu & Hu, 2024; Hu, Ma & Xing, 2023; Kim et al., 2021; Li et al., 2025a; Li et al., 2024; Li et al., 2026; Wei & Liu, 2024; Yang et al., 2024a; Yang et al., 2024b; Yu et al., 2025; Zhang, Chen & Jiang, 2024; Zhao et al., 2024; Zhao, Ji & Wang, 2025).

### Meta-analysis results

### Association of total TyG index with sarcopenia

**Figure 2:**
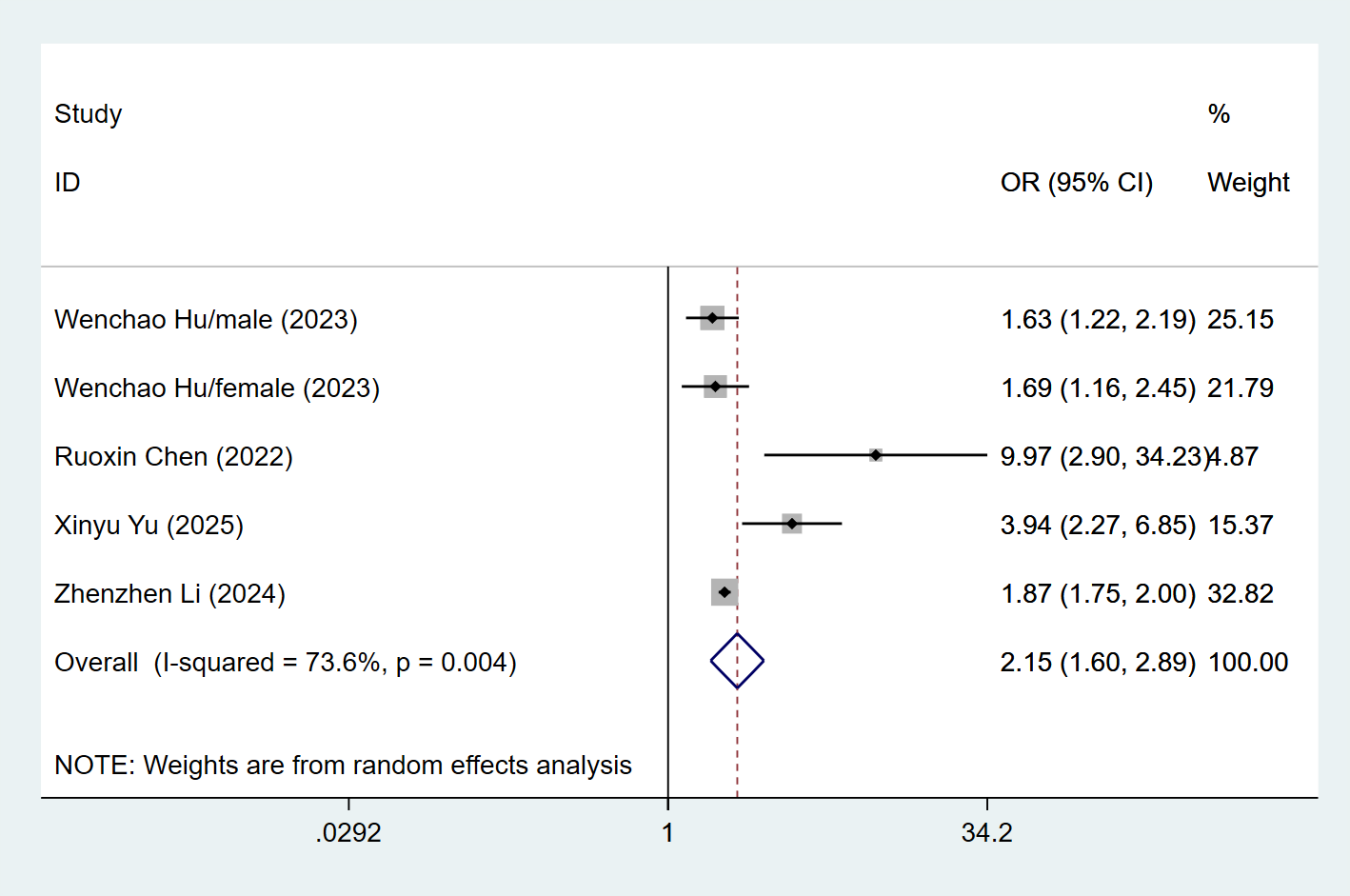
Forest plot of meta-analysis for total TyG index and sarcopenia.

The meta-analysis results indicated that four studies (Chen et al., 2022; Hu, Ma & Xing, 2023; Li et al., 2024; Yu et al., 2025) examined the relationship between sarcopenia and elevated total TyG levels. A random-effects model analysis (*I*^2^ = 73.6%, *P* < 0.01; Fig. 2) revealed a significant association between elevated TyG index levels and sarcopenia (OR = 2.15, 95% CI [1.60–2.89]; *P* = 0.004). Given the substantial heterogeneity (*I*^2^ > 50%), a sensitivity analysis was performed using the leave-one-out method. The results (Fig. S1) show that no individual study exerted a disproportionate influence on the overall association between the total TyG index and sarcopenia. Additionally, the results of Egger’s test (*p* > 0.05; Fig. S2) suggest no significant publication bias among the included studies. However, the funnel plot (Fig. S3) displayed asymmetry, indicating a potential risk of publication bias.

### Association between different dose levels and sarcopenia after stratification of the TyG index by quartiles

### Relationship between TyG-Q2 and sarcopenia

**Figure 3:**
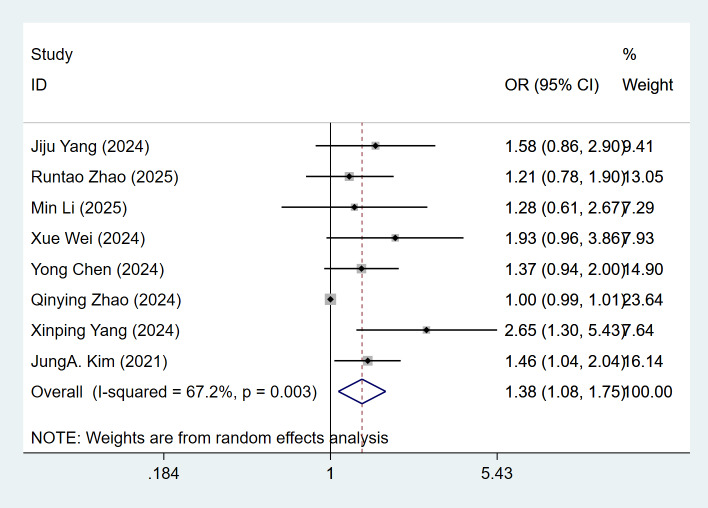
Forest plot of meta-analysis for TyG-Q2 and sarcopenia.

Eight studies (Chen, Liu & Hu, 2024; Kim et al., 2021; Li et al., 2025a; Wei & Liu, 2024; Yang et al., 2024a; Yang et al., 2024b; Zhao et al., 2024; Zhao, Ji & Wang, 2025) provided data on sarcopenia in relation to TyG-Q2 levels. A random-effects model analysis (*I*^2^ = 67.2%, *P* < 0.01) indicated a significant association between TyG-Q2 and sarcopenia (OR = 1.38, 95% CI [1.08–1.75]; *P* = 0.003) (Fig. 3). Due to substantial heterogeneity (*I*^2^ > 50%), a leave-one-out sensitivity analysis was conducted. As shown in Fig. S4, the pooled estimate remained stable within the original confidence intervals following the iterative removal of each study, indicating that no single study disproportionately influenced the results. The association remained largely consistent, with no individual study significantly affecting the robustness of the findings. Additionally, the Egger’s test (Fig. S5) yielded a *P*-value <0.05, and the funnel plot (Fig. S6) displayed right-skewed asymmetry, both suggesting the presence of publication bias.

### Relationship between TyG-Q3 and sarcopenia

**Figure 4:**
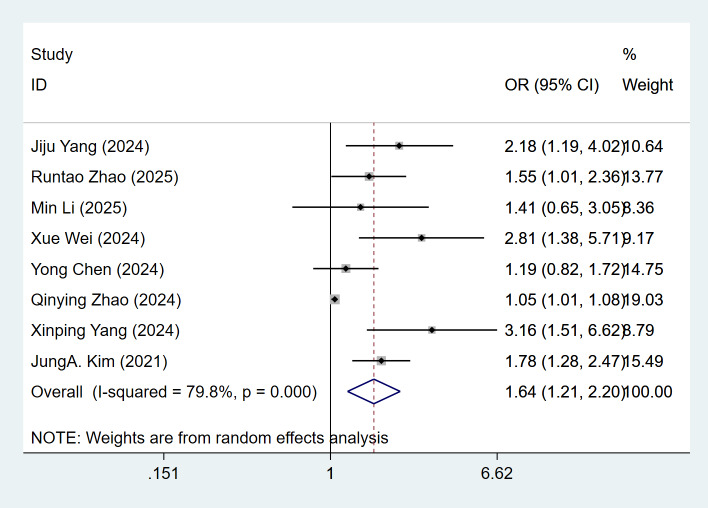
Forest plot of meta-analysis for TyG-Q3 and sarcopenia.

A meta-analysis using a random-effects model (*I*^2^ = 79.8%, *P* < 0.01) revealed a significant association between elevated TyG-Q3 levels and sarcopenia (OR = 1.64, 95% CI [1.21–2.20]; *P* < 0.001) (Fig. 4). Because the *I*^2^ statistic exceeded 50%, a sensitivity analysis was performed by sequentially excluding each study. The findings (Fig. S7) indicate that the association between TyG-Q3 and sarcopenia is robust, with minimal influence from any single study. Moreover, the Egger’s test (*P* < 0.05; Fig. S8) and visual inspection of the funnel plot (Fig. S9) both indicate significant publication bias.

### Relationship between TyG-Q4 and sarcopenia

**Figure 5:**
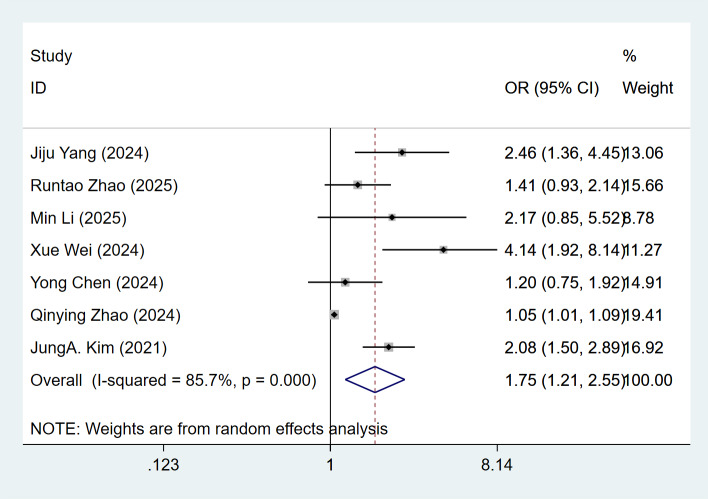
Forest plot of meta-analysis for TyG-Q4 and sarcopenia.

A random-effects model was employed to evaluate the association between the TyG-Q4 and sarcopenia (*I*^2^ = 85.7%, *P* < 0.01). The analysis demonstrated a significant relationship (OR = 1.75, 95% CI [1.21–2.55]; *P* < 0.001) (Fig. 5). Due to the high heterogeneity (*I*^2^ = 85.7%), a sensitivity analysis was performed to assess the stability of the pooled estimates. As shown in Fig. S10, the pooled effect size remained within the original confidence interval after sequentially excluding each study, confirming the robustness of the association against the influence of any single study. Both the Egger’s test (Fig. S11, *P* < 0.05) and the funnel plot (Fig. S12) indicated significant publication bias among the included studies.

### Association between different dose levels of triglyceride-glucose body mass index stratified by quartiles and sarcopenia

### Relationship between TyG-BMI-Q2 and sarcopenia

**Figure 6:**
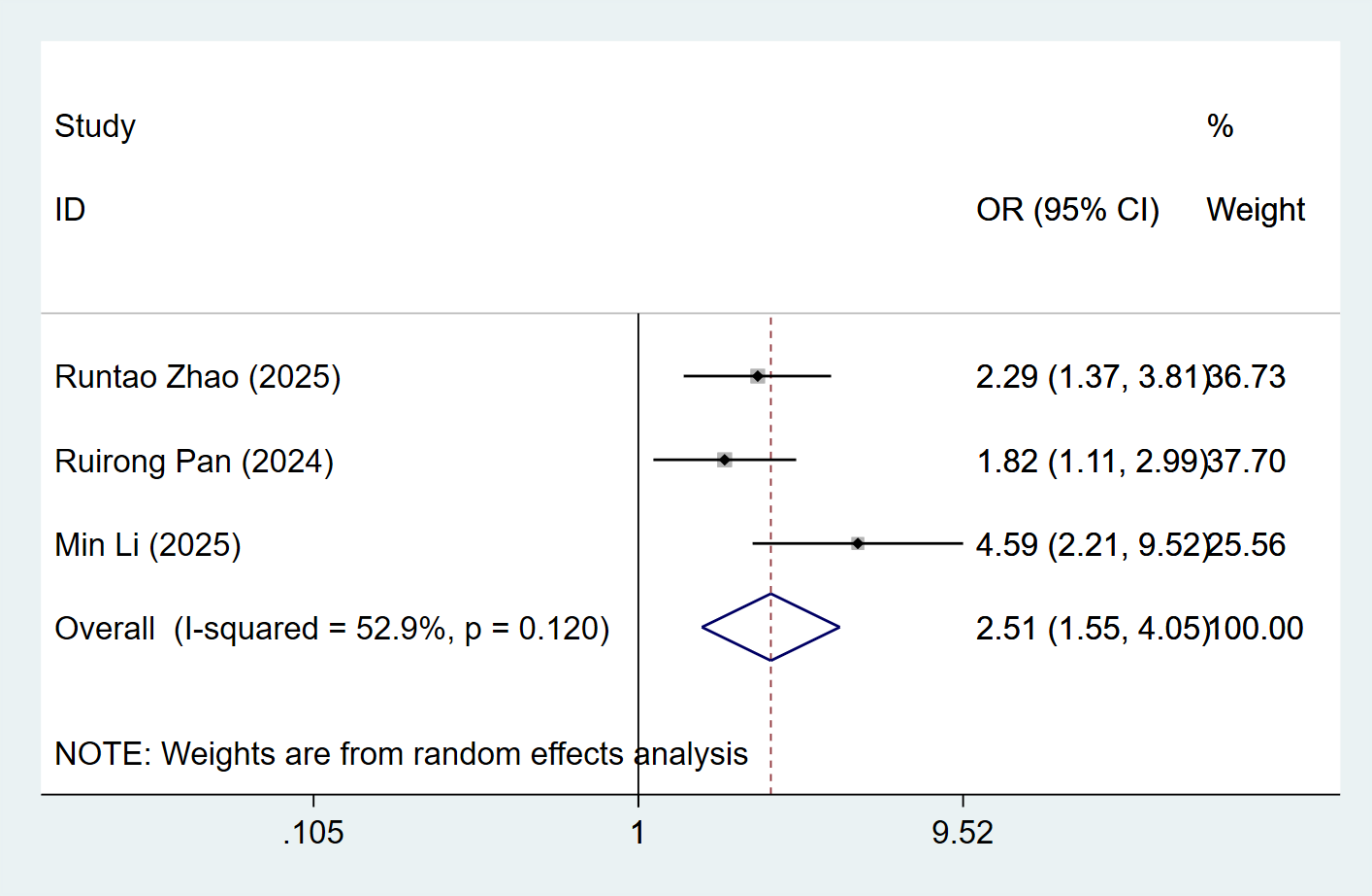
Forest plot of meta-analysis for TyG-BMI-Q2 and sarcopenia.

Three studies (Li et al., 2025a; Pan et al., 2024; Zhao, Ji & Wang, 2025) investigated the association between TyG-BMI-Q2 and sarcopenia. The meta-analysis results (Fig. 6) revealed a significant association between elevated TyG-BMI-Q2 levels and sarcopenia (OR = 2.51, 95% CI [1.55–4.05]). Since the *I*^2^ statistic was greater than 50%, sensitivity analyses excluding individual studies (Fig. S13) showed that the pooled effect estimates remained within the original confidence intervals, indicating stability and that the results were not influenced by any single study. The Egger’s test (*P* > 0.05, Fig. S14) and the funnel plot (Fig. S15) revealed no significant publication bias.

### Relationship between TyG-BMI-Q3 and sarcopenia

**Figure 7:**
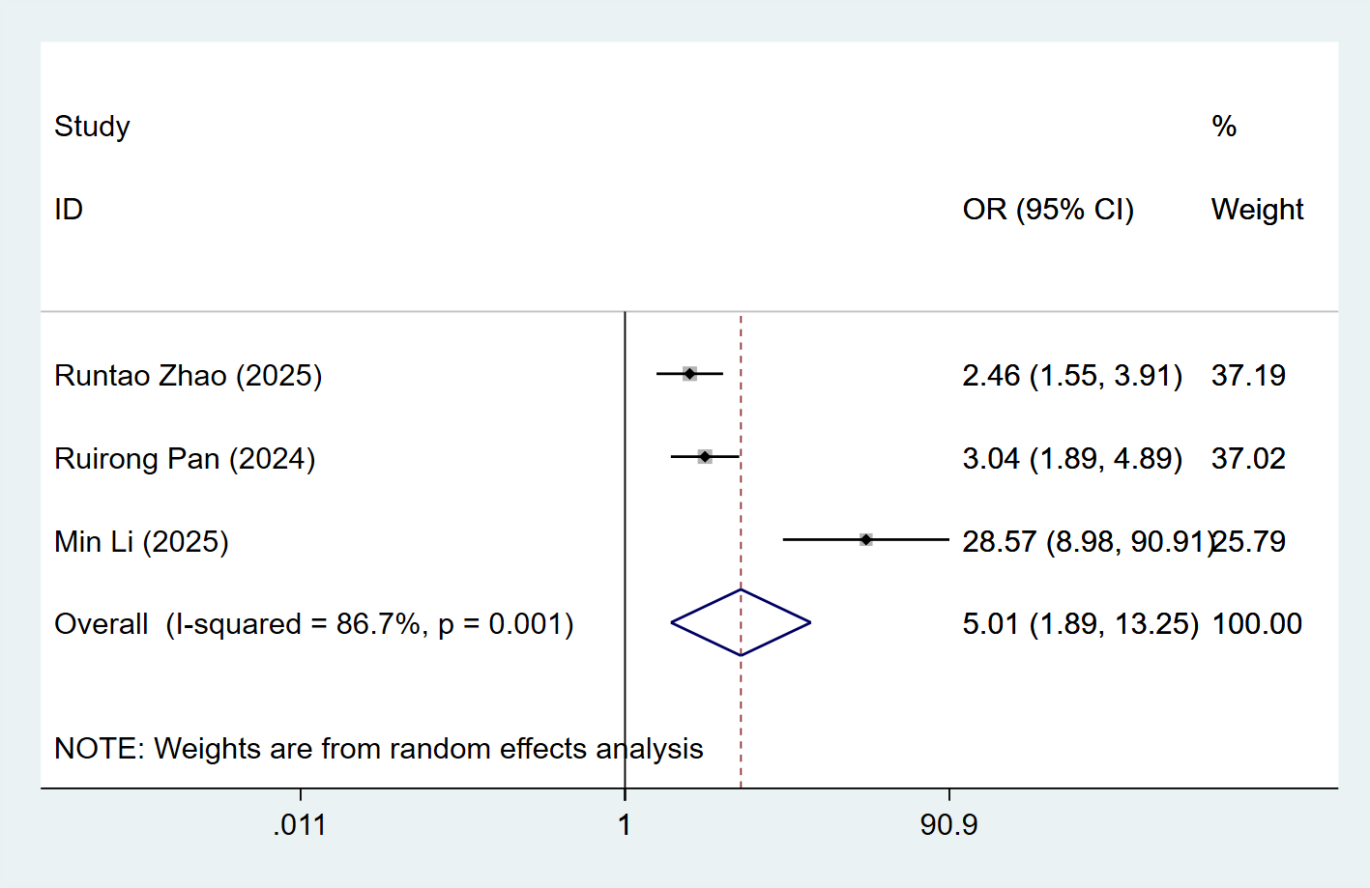
Forest plot of meta-analysis for TyG-BMI-Q3 and sarcopenia.

The forest plot results (Fig. 7) indicate that, using a random-effects model (*I*^2^ = 86.7%, *P* < 0.01), higher TyG-BMI-Q3 levels were significantly associated with sarcopenia (OR = 5.01, 95% CI [1.89–13.25]; *P* = 0.001). Because *I*^2^ exceeded 50%, a sensitivity analysis was conducted by sequentially removing individual studies to determine whether the pooled effect size remained within the original confidence interval. The results (Fig. S16) showed that excluding the studies by Zhao, Ji & Wang (2025) and Pan et al. (2024) caused the effect size to fall outside the original confidence interval (95% CI [12.50–26.82]), indicating that these studies affected the robustness of the overall association. The Egger’s test (Fig. S17, *P* > 0.05) and the funnel plot (Fig. S18) suggested no significant publication bias among the included studies.

### Relationship between TyG-BMI-Q4 and sarcopenia

**Figure 8:**
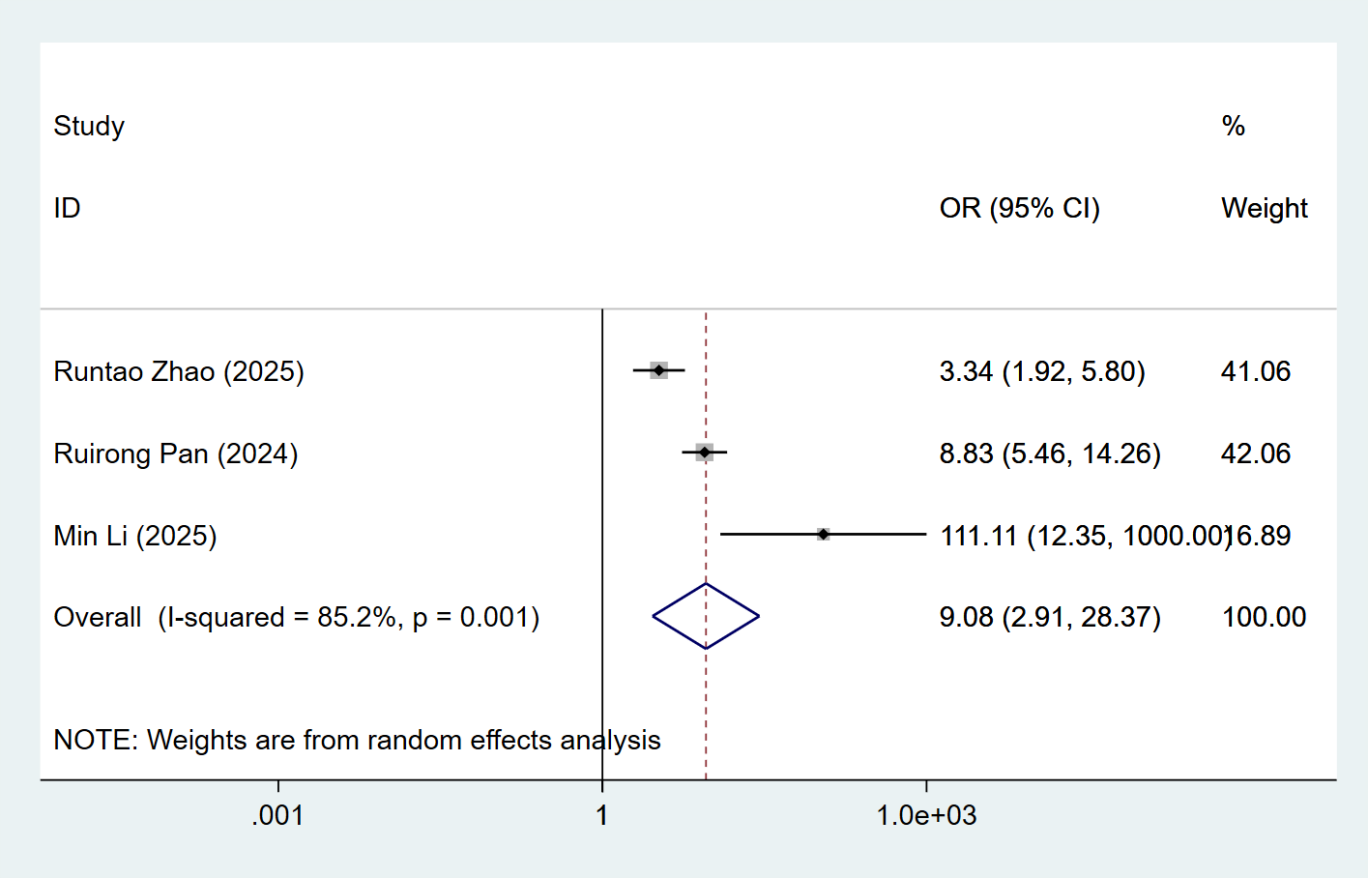
Forest plot of meta-analysis for TyG-BMI-Q4 and sarcopenia.

According to the meta-analysis results (Fig. 8), when evaluated using a random-effects model (*I*^2^ = 85.2%, *P* < 0.01), a higher TyG-BMI-Q4 index level was significantly associated with sarcopenia (OR = 9.08, 95% CI [2.91–28.37]; *P* = 0.001). Since the *I*^2^ statistic exceeded 50%, a sensitivity analysis was conducted to assess the robustness of the findings across the included studies. The results (Fig. S19) showed that, after sequentially excluding individual studies, the pooled effect estimates remained within the original confidence intervals, indicating that no single study had an undue influence on the overall findings. However, in the TyG-BMI-Q4 analysis, the leave-one-out sensitivity analysis revealed notable fluctuations in the pooled effect sizes, suggesting potential statistical instability. This instability may be due to the small number of included studies, so these results should be interpreted with caution. The Egger’s test (Fig. S20, *P* > 0.05) and the funnel plot (Fig. S21) indicated no significant publication bias among the three included studies. The Egger’s test (Fig. S20) (*P* > 0.05) and the funnel plot (Fig. S21) outcomes suggested no significant publication bias among the three included studies.

### Subgroup analysis

To investigate potential sources of heterogeneity, we conducted subgroup analyses based on several key factors, including study population, sarcopenia diagnostic criteria, comorbidities, and TyG index measurement methods. These analyses aimed to determine how these factors influence the relationship between the TyG index and sarcopenia. The subgroup analysis results for sarcopenia diagnostic criteria (Fig. S22) indicated that sarcopenia diagnosed using the AWGS 2019 criteria was significantly associated with the TyG index (OR = 5.28, 95% CI [2.27–12.3]). Sarcopenia, defined using the SMI criteria, was significantly associated with the TyG index (OR = 1.65, 95% CI [1.32–2.08]). Subgroup analysis of middle-aged individuals (Fig. S23) showed that sarcopenia in this population was significantly linked to the TyG index (OR = 1.87, 95% CI [1.46–2.42], *P* = 0.043). In the subgroup with comorbidities (Fig. S24), sarcopenia was significantly associated with TyG index levels (OR = 1.94, 95% CI [1.54–2.44], *P* = 0.044). In the subgroup with different TyG index measurement methods (Fig. S25), the TyG index, calculated as the product of fasting triglycerides and fasting blood glucose, was also significantly associated with sarcopenia (OR = 2.14, 95% CI [1.24–3.71], *P* = 0.019).

### Meta-regression analysis

To further assess the impact of potential clinical and methodological factors on between-study heterogeneity, univariate meta-regression analyses were conducted using the following covariates: study population (middle-aged *vs.* older adults), sarcopenia diagnostic criteria (AWGS 2019 *vs.* other criteria), comorbidities (presence *vs.* absence), and TyG index measurement methods (varied calculation formulas). The meta-regression analysis showed that only the sarcopenia diagnostic criteria significantly increased the effect size. Specifically, the use of the AWGS 2019 criteria demonstrated a stronger association between the TyG index and sarcopenia. None of the other moderators—such as study population, comorbidities, or TyG index measurement methods—were statistically significant. For further details, please refer to the Supplementary Materials and Table 3.

**Table 3:** Meta-regression results for the association between the TyG index and sarcopenia.

Variables	Coefficient (exp(b))	95% Confidence interval	Std. Err.	*P* value
study population1(middle-aged people)	0.47	(0.11, 2.09)	0.22	0.207
study population2(older people)	2.12	(0.48, 9.39)	0.99	0.207
comorbidities1(no)	5.04	(0.37, 67.85)	4.12	0.142
comorbidities2(yes)	0.2	(0.01, 2.67)	0.16	0.142
TyG measurement method 1	2.12	(0.48, 9.39)	0.99	0.207
TyG measurement method 2	0.68	(0.05, 8.36)	0.53	0.656
TyG measurement method 3	0.93	(0.99, 8.69)	0.65	0.921
diagnostic criteria1(ASMI)	0.68	(0.05, 8.36)	0.53	0.656
diagnostic criteria2(AWGS2019)	2.49	(1.09, 5.67)	0.64	0.039
diagnostic criteria3(SMI)	0.51	(0.10, 2.70)	0.27	0.290

## Discussion

### Summary of main results

This meta-analysis included 14 eligible cross-sectional studies comprising a total of 82,798 participants. The results indicate that both the TyG index and the TyG-BMI index are significantly associated with sarcopenia. This association was confirmed through multiple subgroup analyses stratified by study population, sarcopenia diagnostic criteria, comorbidities, and TyG measurement methods. To our knowledge, this study represents one of the earliest comprehensive meta-analyses to investigate the potential relationship between sarcopenia and the TyG index.

### Mechanisms linking the TyG index, insulin resistance, and sarcopenia

Our meta-analysis demonstrated a significant association between elevated TyG index levels and sarcopenia, with a progressively increasing trend across TyG quartiles. This graded pattern suggests a potential biological gradient between insulin resistance and muscle loss. Insulin resistance, indicated by an elevated TyG index, impairs the PI3K/Akt/mTOR signaling pathway, which is crucial for muscle protein synthesis (Shi & Chen, 2025). This finding directly indicates that individuals in higher TyG quartiles show progressively stronger associations with sarcopenia. Moreover, pro-inflammatory mediators elevated in insulin-resistant states—such as TNF-*α*, IL-6, and CRP—activate pathways that inhibit protein synthesis and promote protein degradation (Tu et al., 2025). This inflammatory mechanism underpins the strong association observed across TyG quartiles. In summary, the association between the TyG index and sarcopenia is biologically plausible, mediated by impaired protein synthesis, enhanced proteolysis, and metabolic dysfunction. These interconnected mechanisms collectively explain the consistent relationship observed in this meta-analysis.

### Discussion of key results

This meta-analysis revealed three key findings. First, a significant association between the TyG index and sarcopenia was identified. Second, a progressively stronger association was observed across TyG quartiles. Third, the TyG-BMI index exhibited a notably stronger association, particularly at higher levels. These findings are consistent with previous studies by Xiao et al. (2025) and Zhao et al. (2025a), both of which reported positive associations between the TyG index and sarcopenia. Sensitivity analysis and publication bias assessment indicate that, despite some publication bias across studies, the association between the TyG index and sarcopenia remains stable and robust. The significantly stronger association between TyG-BMI warrants special attention. This finding may reflect the combined contribution of insulin resistance and adiposity to the development of sarcopenia. One study has proposed the concept of the “obesity paradox”, suggesting that a higher body mass index (BMI) may, in fact, be a risk factor for sarcopenia. The study emphasizes the importance of considering obesity when comparing definitions of sarcopenia (Liu et al., 2023). Since BMI is a known risk factor for sarcopenia, the association between TyG-BMI and sarcopenia appears even stronger. However, sensitivity analysis indicated that this estimate was unstable, with significant fluctuations observed during leave-one-out analysis. This instability suggests that the true effect size may be smaller and that the results could be influenced by the limited number of studies included in this analysis. Therefore, while TyG-BMI shows promise, these findings should be regarded as preliminary and necessitate further validation in larger studies. The association between the TyG index and sarcopenia observed in this meta-analysis aligns with emerging evidence linking insulin resistance to sarcopenic obesity (SO) (Kim et al., 2023; Xu et al., 2024). Zhao et al. (2025b) reported a progressive increase in the incidence of SO with higher TyG index values, suggesting that the metabolic dysfunction reflected by the TyG index contributes to both muscle loss and adiposity. However, one study reported findings that were inconsistent with our meta-analysis, showing an inverse association between the sarcopenia index and the TyG index in hospitalized older adults (mean age 85 years) (Ren et al., 2022). This discrepancy likely arises from differences in study populations and methodologies. In that study, sarcopenia was diagnosed solely based on calf circumference and grip strength, with the sarcopenia index used as a proxy for muscle mass. In this elderly cohort, significant muscle loss and reduced metabolic demands may have resulted in lower glucose and triglyceride utilization, leading to decreased TyG index values. Consequently, a higher sarcopenia index was inversely correlated with TyG levels. These findings emphasize the impact of variations in study populations and diagnostic criteria, which can lead to significantly different research outcomes.

Subgroup analyses further clarified how key factors modulate the relationship between TyG and sarcopenia. In the subgroup meta-analyses using three different diagnostic criteria, both the SMI and AWGS 2019 criteria demonstrated significant associations with sarcopenia. However, the association between the TyG index and sarcopenia appeared more pronounced under the AWGS 2019 criteria compared to the SMI criteria. Consistent with the subgroup findings, the meta-regression analysis indicated that the sarcopenia diagnostic criteria were a significant source of between-study heterogeneity. Adopting the AWGS 2019 criteria resulted in a stronger association between the TyG index and sarcopenia. This can be attributed to the AWGS 2019 criteria, which require the concurrent loss of muscle mass, decline in muscle strength, and functional impairment for the diagnosis of sarcopenia. In contrast, the SMI criteria rely solely on muscle mass calculations. A cross-sectional study by Yuan & Larsson (2023) showed that, before the introduction of the 2010 EWGSOP diagnostic criteria, many studies defined sarcopenia solely based on low muscle mass—a unidimensional approach that was later deemed insufficient for accurately reflecting muscle function. A 2024 meta-analysis by Weng et al. (2025) recommended that current diagnostic criteria adopt a more comprehensive assessment framework, including not only low muscle mass but also measures of muscle strength and function. Our subgroup analysis further indicated that elevated TyG index levels in middle-aged individuals were significantly associated with sarcopenia. A recent cross-sectional study (Zhou et al., 2025) suggests that muscle loss may begin in young adulthood, with a more pronounced trend in both middle-aged and younger adults. Furthermore, multiple studies estimate that approximately 30% of middle-aged adults may experience accelerated muscle decline due to factors such as prolonged physical inactivity, inadequate nutrition, and chronic inflammation. Consequently, the prevalence of sarcopenia progressively increases as adults reach middle age (Feng et al., 2024; Jung, Jung & Hwang, 2023; Lee et al., 2024; Silva & Mulder, 2021). A substantial body of evidence indicates that middle-aged and older adults with chronic conditions, including diabetes, cardiovascular disease, and respiratory disorders, are more vulnerable to developing sarcopenia. In 2020, a meta-analysis by Pacifico et al. (2020) found that, even after accounting for differences in diagnostic criteria, individuals with comorbidities such as dementia, diabetes, and respiratory diseases had a significantly higher incidence of sarcopenia than those without these conditions. An additional meta-analysis (Qiao et al., 2021) confirmed that higher glycated hemoglobin levels, prediabetes or diabetes, and related complications are associated with a significantly increased incidence of sarcopenia. In conclusion, these findings suggest that middle-aged and older adults with chronic diseases are more vulnerable to developing sarcopenia.

This meta-analysis underscores the clinical relevance of the TyG index in assessing sarcopenia. Its associated indicators demonstrate promising potential for the early identification of low muscle mass. As a simple and reliable biochemical marker, the TyG index is valuable for initial sarcopenia screening. Prospective studies are warranted to further investigate the mechanistic links between TyG levels and sarcopenia.

### Strengths and limitations

This meta-analysis has three key methodological strengths. First, it provides the first comprehensive evaluation of the association between the TyG index and sarcopenia and extends this assessment to include its derived indicator, the TyG-BMI index. Second, using subgroup and meta-regression analyses, we examined potential confounding factors that may influence the relationship between the TyG index and sarcopenia, thereby offering new directions for future research. Furthermore, this study examines the association between sarcopenia and both the TyG index and the TyG-BMI index, categorized by quartiles. This multi-level, multi-indicator analytical approach more clearly illustrates the trends in sarcopenia across different exposure levels of the TyG and TyG-BMI indices, providing quantitative evidence to inform clinical practice. However, several limitations should be acknowledged. First, because all included studies are cross-sectional, the possibility of reverse causality cannot be ruled out. Therefore, caution is advised when inferring causal relationships between the TyG index and sarcopenia. Second, all the cross-sectional studies included in this review were conducted in Asian populations, with no representation from regions such as the Americas or Europe. This limitation restricts the generalizability of the findings. Third, we conducted meta-regression analyses. However, due to the limited number of studies available for each covariate, these analyses may lack statistical power. As a result, the meta-regression findings should be interpreted with caution. Fourth, the quartile cutoffs for the TyG and TyG-BMI indices varied across the included studies, reflecting differences in the underlying study populations. No standardization of these cutoffs was performed; instead, we used the quartile definitions specific to each study as reported in the original articles. This inherent variability in cutoffs may contribute to methodological heterogeneity. Thus, the findings should be interpreted with caution. Furthermore, the TyG-BMI Q4 analysis revealed an unusually high odds ratio with a wide confidence interval, suggesting potential small-study effects and statistical instability due to the weak association between the TyG index and sarcopenia. All included studies were cross-sectional, limiting the ability to fully account for the confounding effect of BMI. Therefore, the findings of this study should be interpreted with caution. In conclusion, the findings highlight the need for further rigorously designed, standardized prospective studies to better understand the sources of heterogeneity. Given the publication bias, considerable heterogeneity, and the cross-sectional design of the analyzed studies, future research should prioritize large-scale, multi-ethnic prospective cohort studies across diverse geographic regions to explore the causal relationship and underlying mechanisms between TyG index levels and sarcopenia.

## Conclusion

This meta-analysis confirms a significant association between the TyG index and sarcopenia. These findings highlight the potential utility of the TyG index in assessing sarcopenia. However, due to publication bias, substantial heterogeneity, and the cross-sectional design of the analyzed studies, future research should focus on large-scale, multi-ethnic prospective cohort studies conducted across diverse geographic regions to clarify the causal relationship and underlying mechanisms between the TyG index and sarcopenia.

## Supplemental Information

10.7717/peerj.21424/supp-1 Supplemental Information 1 Supplementary- Materials

10.7717/peerj.21424/supp-2 Supplemental Information 2 Total TyG Index and Sarcopenia sensitivity analysis

10.7717/peerj.21424/supp-3 Supplemental Information 3 TyG-Q2 and Sarcopenia sensitivity analysis

10.7717/peerj.21424/supp-4 Supplemental Information 4 TyG-Q3 Index and Sarcopenia sensitivity analysis

10.7717/peerj.21424/supp-5 Supplemental Information 5 TyG-Q4 Index and Sarcopenia sensitivity analysis

10.7717/peerj.21424/supp-6 Supplemental Information 6 TyG-BMI-Q2 and sarcopenia sensitivity analysis

10.7717/peerj.21424/supp-7 Supplemental Information 7 TyG-BMI-Q3 and sarcopenia sensitivity analysis

10.7717/peerj.21424/supp-8 Supplemental Information 8 TyG-BMI-Q4 and sarcopenia sensitivity analysis

10.7717/peerj.21424/supp-9 Supplemental Information 9 Subgroup analysis by TyG measurement method

10.7717/peerj.21424/supp-10 Supplemental Information 10 The trim and fill funnel plot of Total TyG Index and Sarcopenia

10.7717/peerj.21424/supp-11 Supplemental Information 11 The trim and fill funnel plot of TyG-Q2 and Sarcopenia

10.7717/peerj.21424/supp-12 Supplemental Information 12 The trim and fill funnel plot of TyG-Q3 and Sarcopenia

10.7717/peerj.21424/supp-13 Supplemental Information 13 The trim and fill funnel plot of TyG-Q4 and Sarcopenia

10.7717/peerj.21424/supp-14 Supplemental Information 14 The trim and fill funnel plot of TyG-BMI-Q2 and Sarcopenia

10.7717/peerj.21424/supp-15 Supplemental Information 15 The trim and fill funnel plot of TyG-BMI-Q3 and Sarcopenia

10.7717/peerj.21424/supp-16 Supplemental Information 16 The trim and fill funnel plot of TyG-BMI-Q4 and Sarcopenia

10.7717/peerj.21424/supp-17 Supplemental Information 17 PRISMA checklist

## List of abbreviations

TyG: triglyceride-glucose index
CLIP: chronic low-grade inflammation
IR: insulin resistance
MeSH: Medical Subject Headings
SO: sarcopenic obesity
AWGS: Asian Working Group for Sarcopenia
SMI: Skeletal Muscle Mass Index
EWGSOP: European Working Group on Sarcopenia in Older People
TyG-BMI: Triglyceride-glucose Body Mass Index
FNIH: Foundation for the National Institutes of Health

## Additional Information and Declarations

### Data Availability

The following information was supplied regarding data availability: This is a Systematic Review/Meta-analysis.
