# Supplementary material for: Association between triglyceride-glucose index and sarcopenia: a meta-analysis
Source: PeerJ. 2026 Jul 23;14:e21424. doi: 10.7717/peerj.21424 (PMC13401845; doi:10.7717/peerj.21424)
Supplement: Supplemental Information 1 [file peerj-14-21424-s001.docx]

Table S1 search strategy

((sarcopenia[MeSH Terms]) OR ((sarcopenia[Title/Abstract]) OR (sarcopenias[Title/Abstract]))) AND (((((TyG index[Title/Abstract]) OR (triglyceride-glucose index[Title/Abstract])) OR ("triglyceride and glucose index"[Title/Abstract])) OR (triglyceride glucose index[Title/Abstract])) OR (triacylglycerol glucose index[Title/Abstract]))


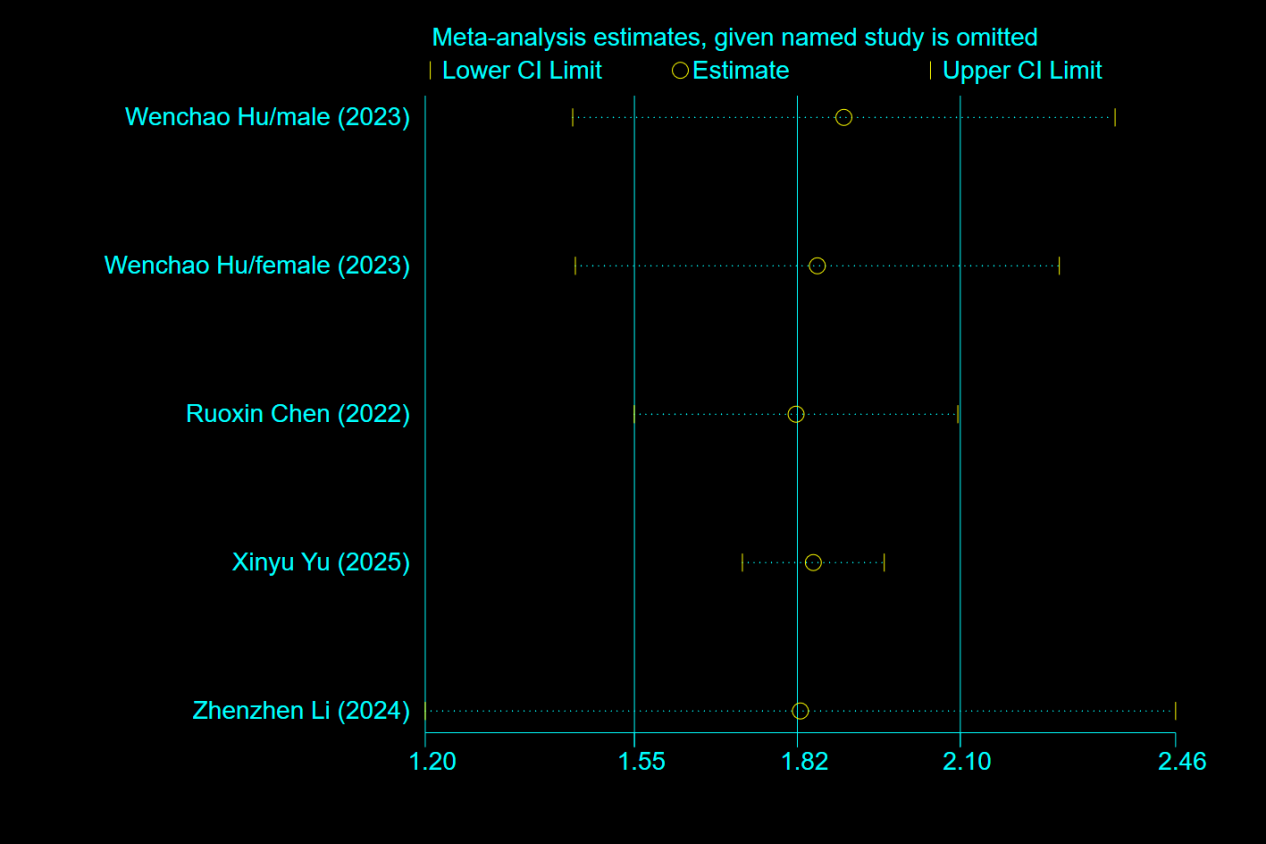


Figure S1 Total TyG Index and Sarcopenia sensitivity analysis


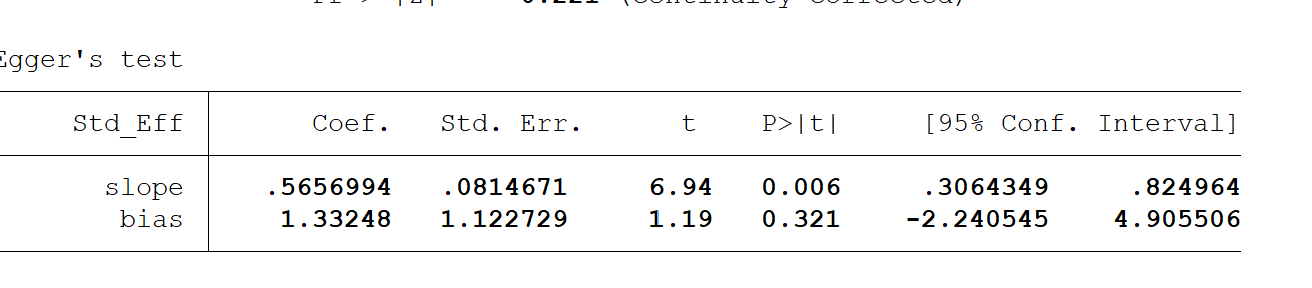


Figure S2egger’s test of Total TyG Index and Sarcopenia


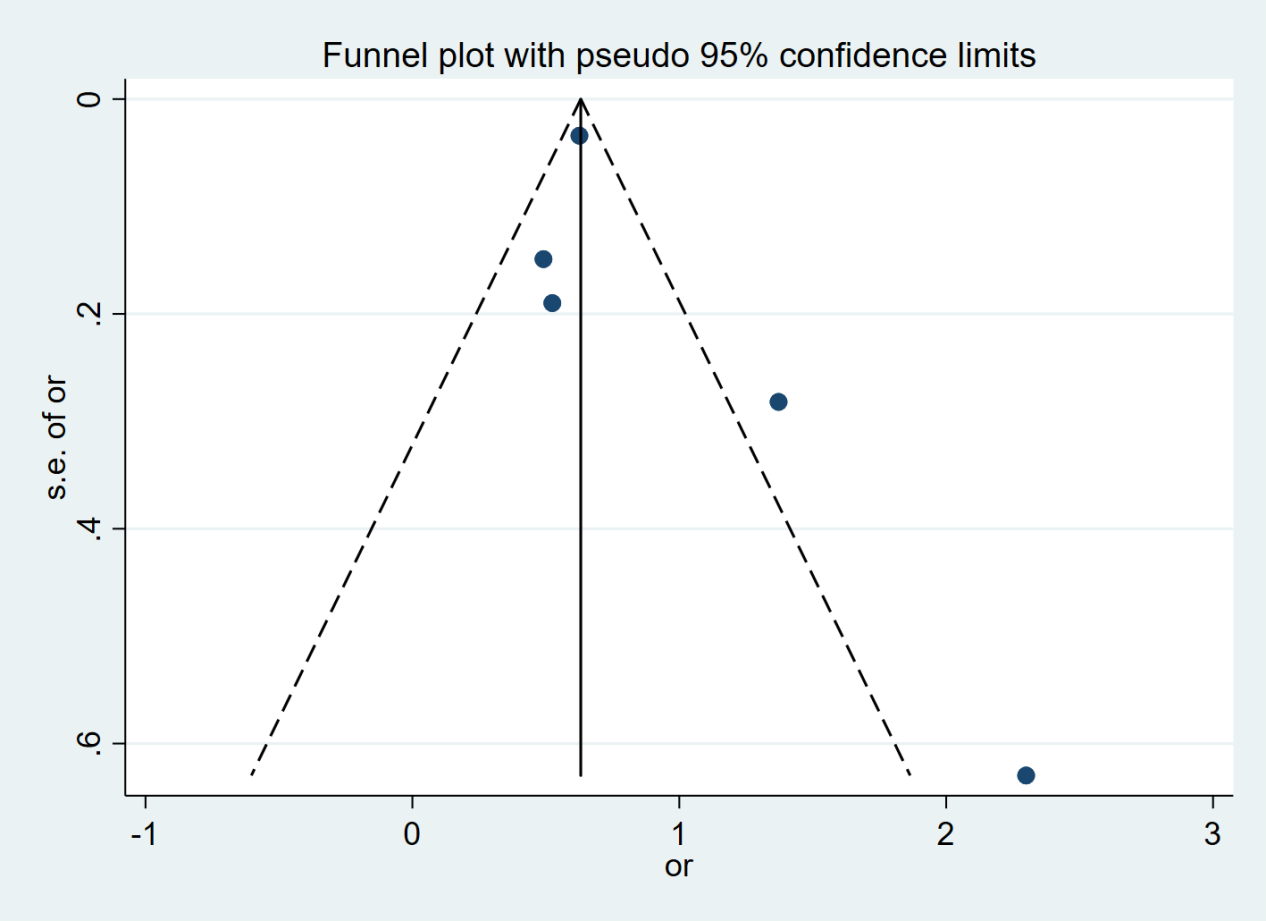


Figure S3 Meta-analysis funnel plot of Total TyG Index and Sarcopenia


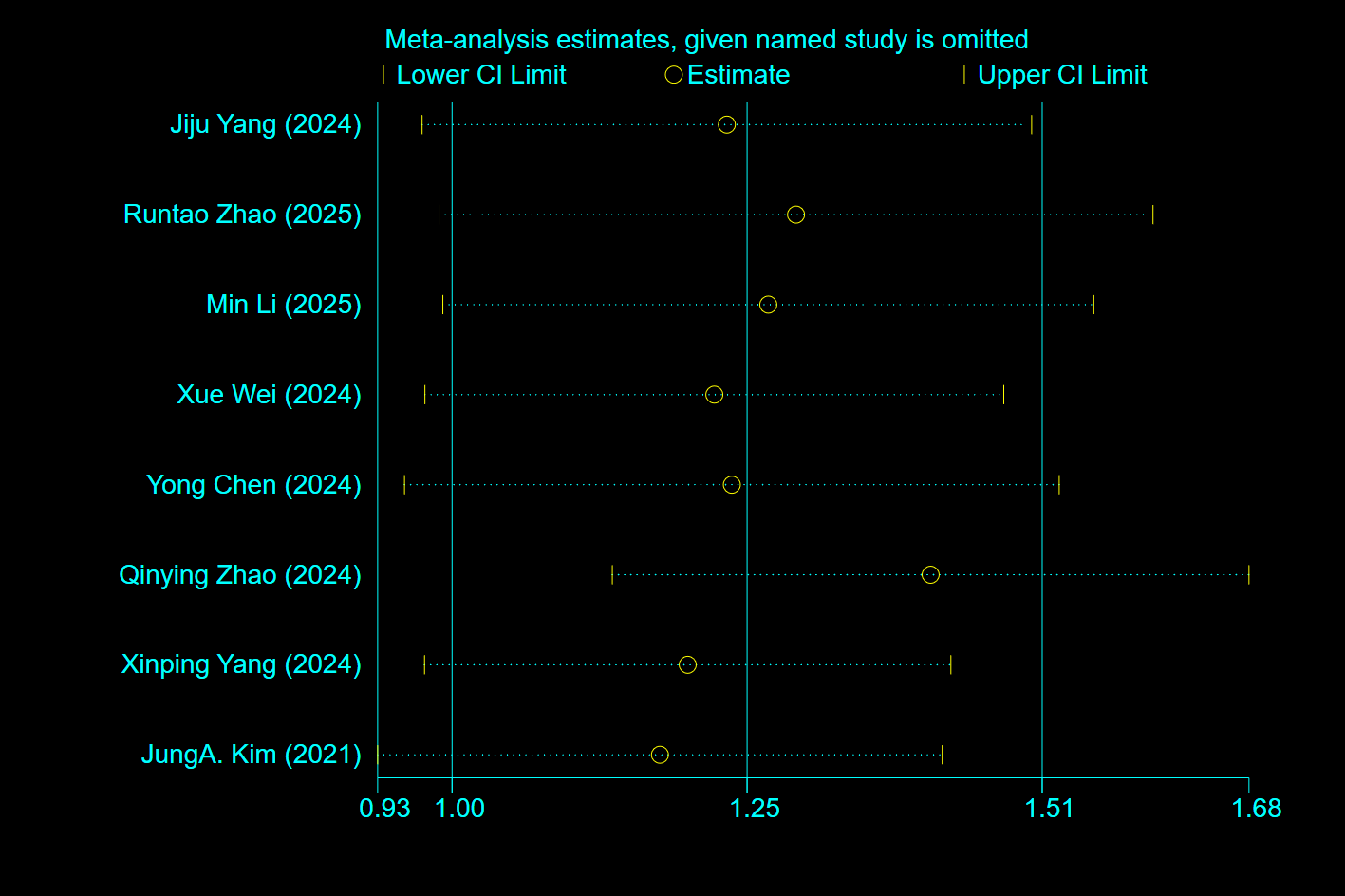


Figure S4 TyG-Q2 and Sarcopenia sensitivity analysis


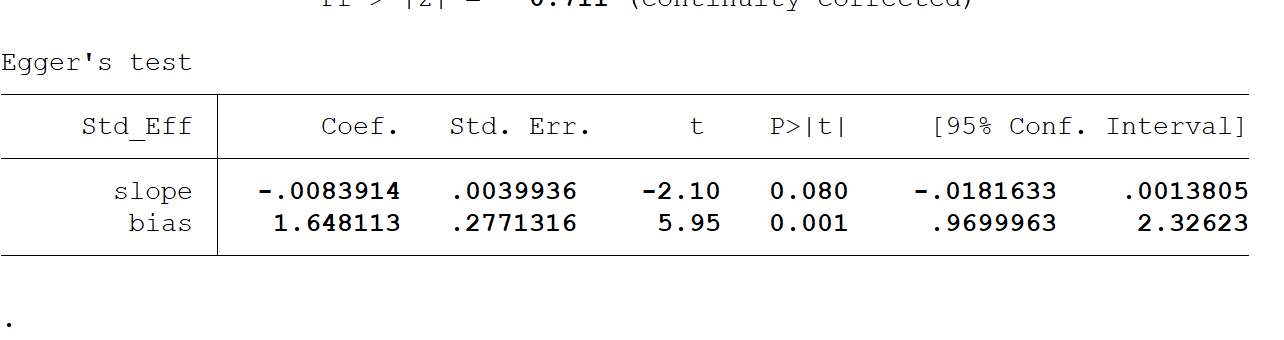


Figure S5 egger’s test of TyG-Q2 Index and Sarcopenia


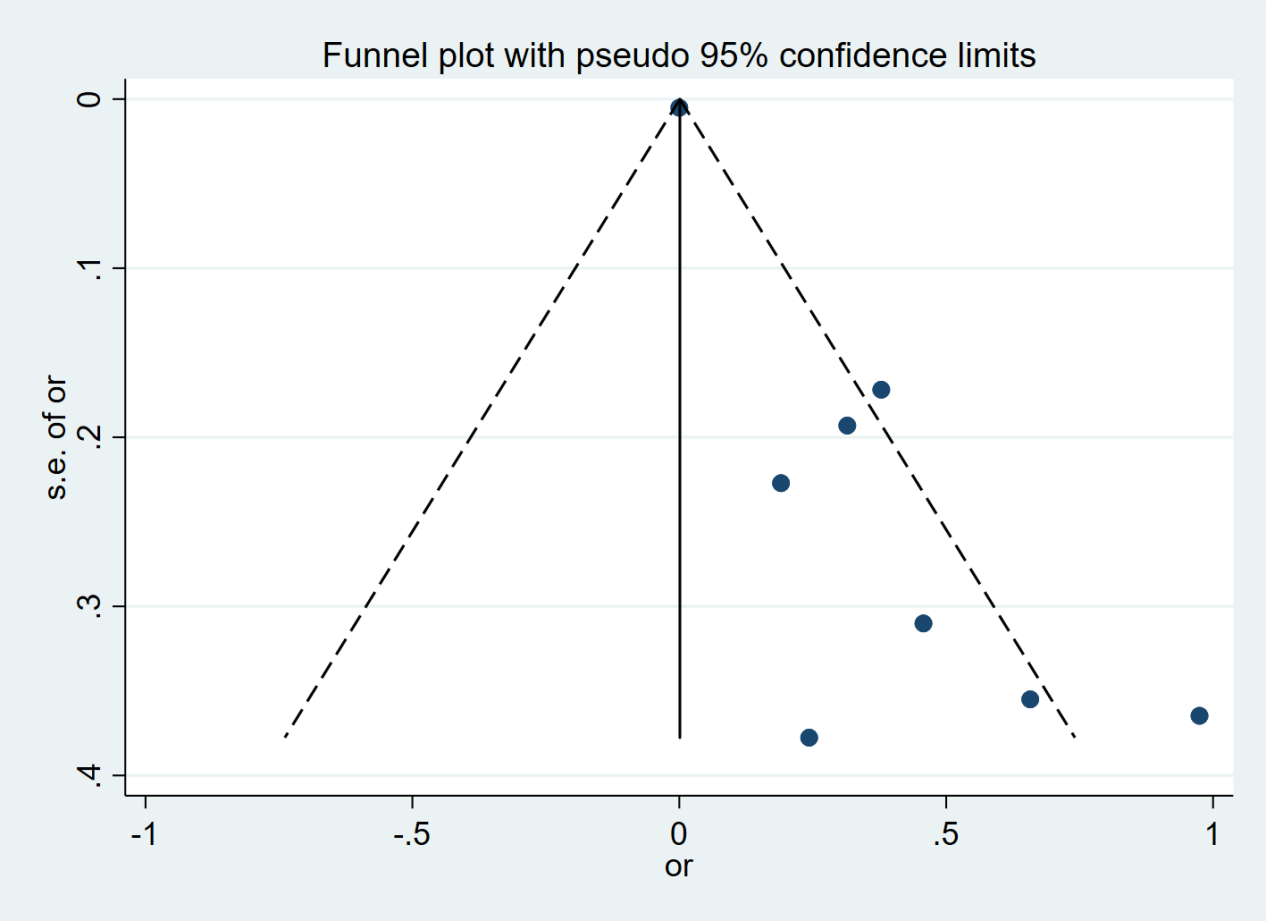


Figure S6 Meta-analysis funnel plot of TyG-Q2 Index and Sarcopenia


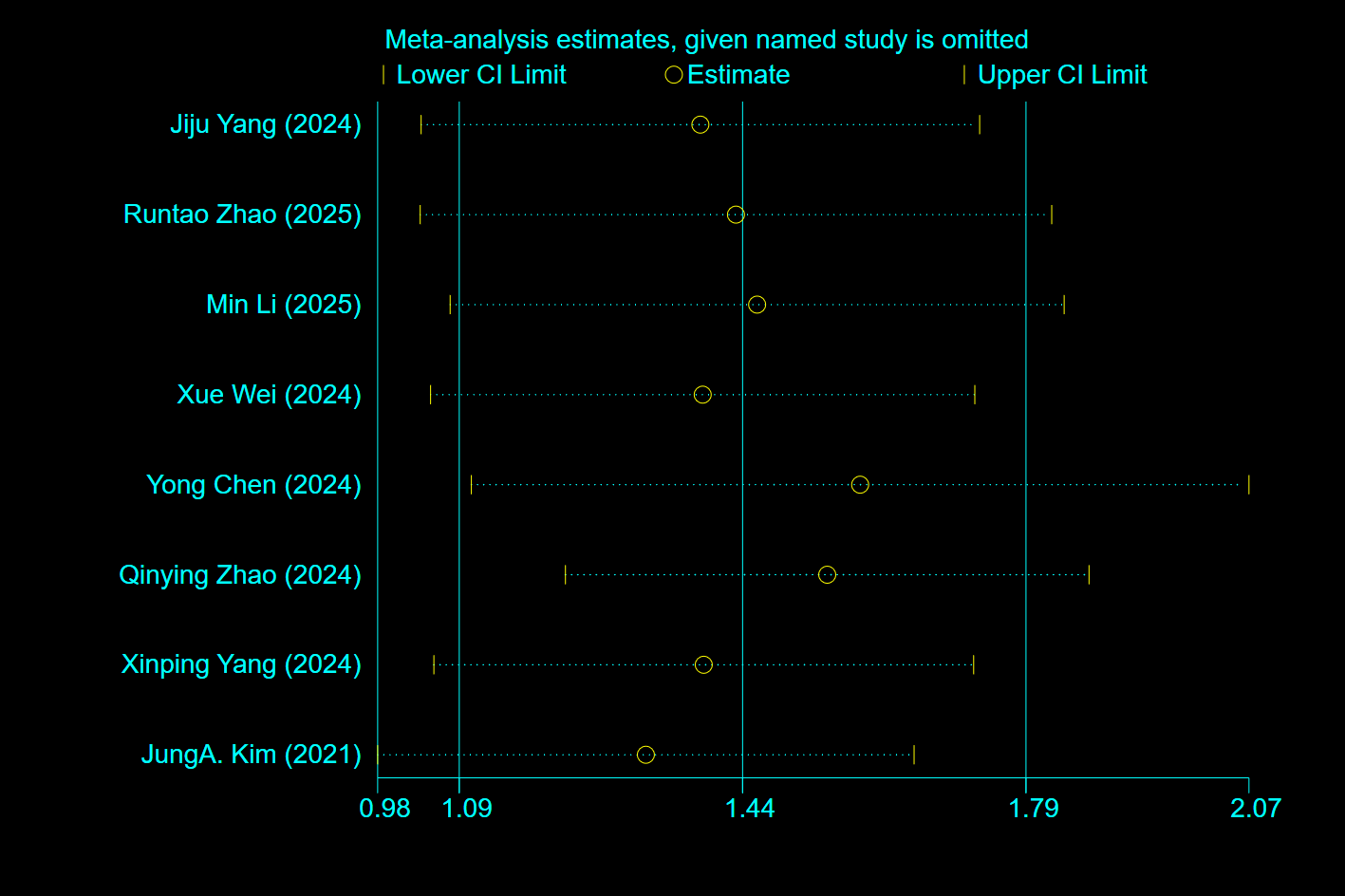
Figure S7 TyG-Q3 Index and Sarcopenia sensitivity analysis


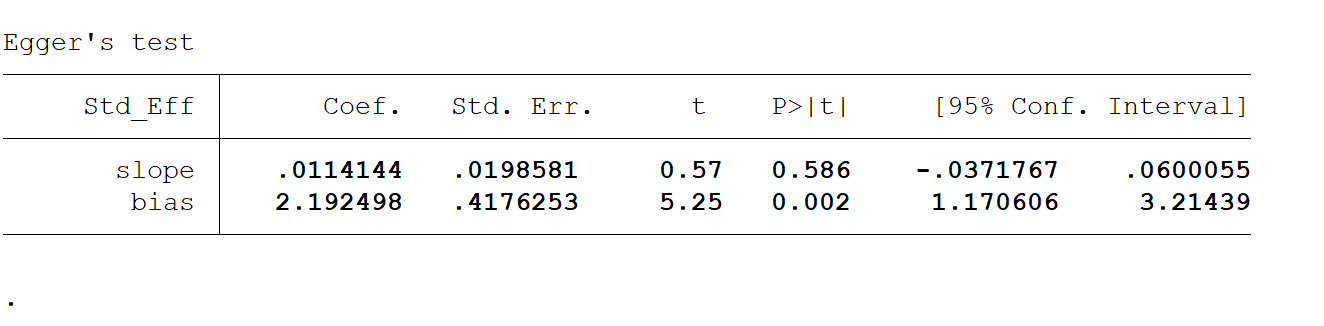
Figure S8 egger’s test of TyG-Q3 Index and Sarcopenia


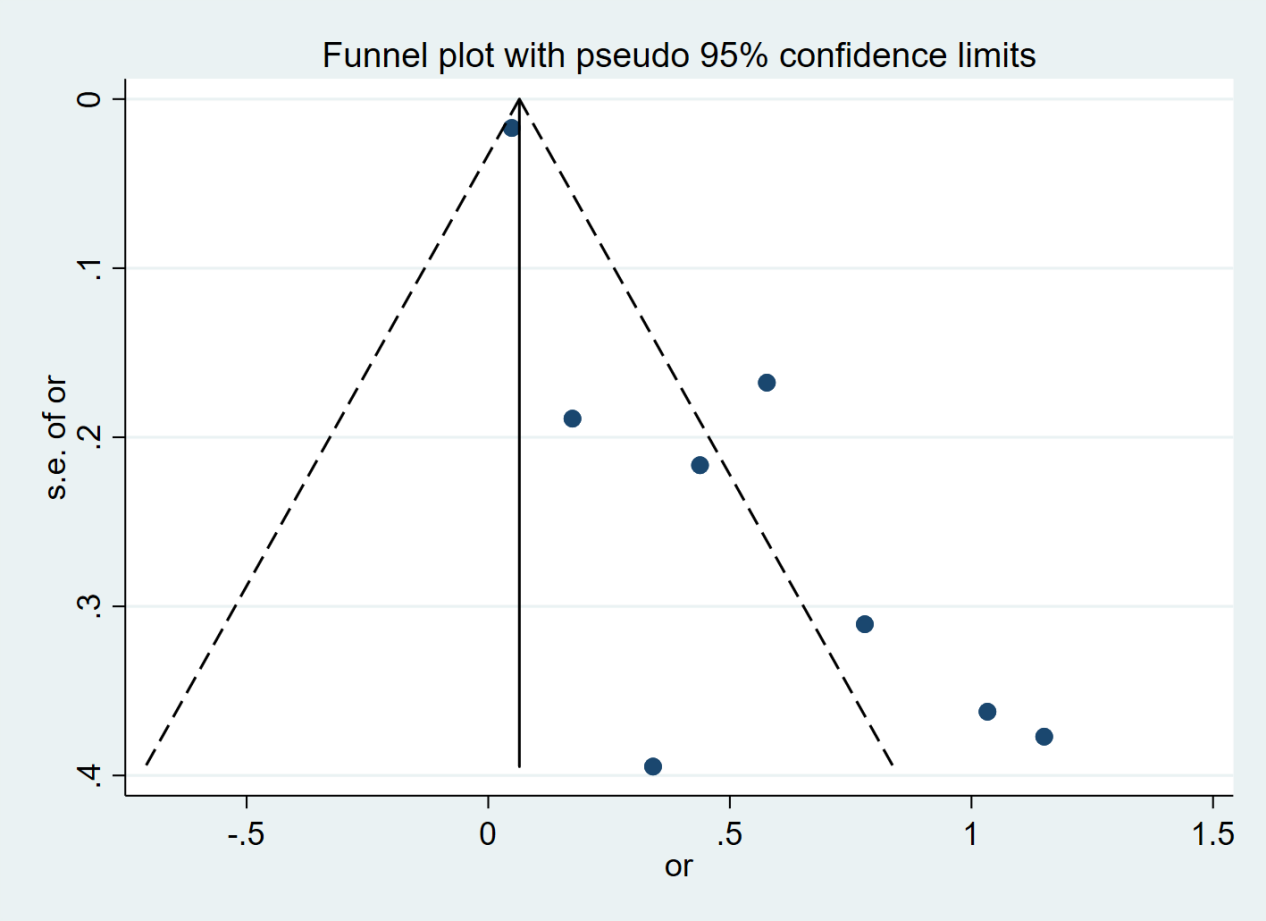
Figure S9 Meta-analysis funnel plot of TyG-Q3 Index and Sarcopenia


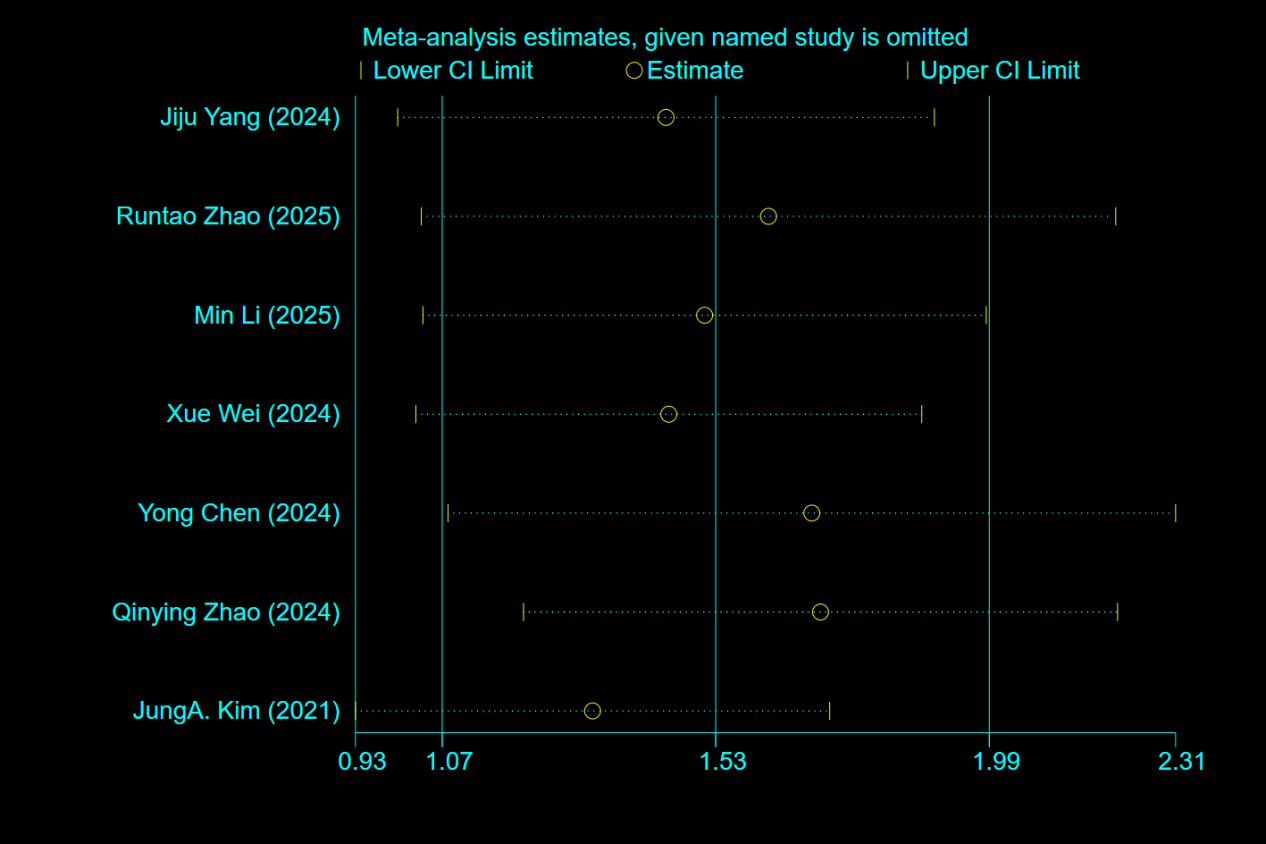


Figure S10 TyG-Q4 Index and Sarcopenia sensitivity analysis


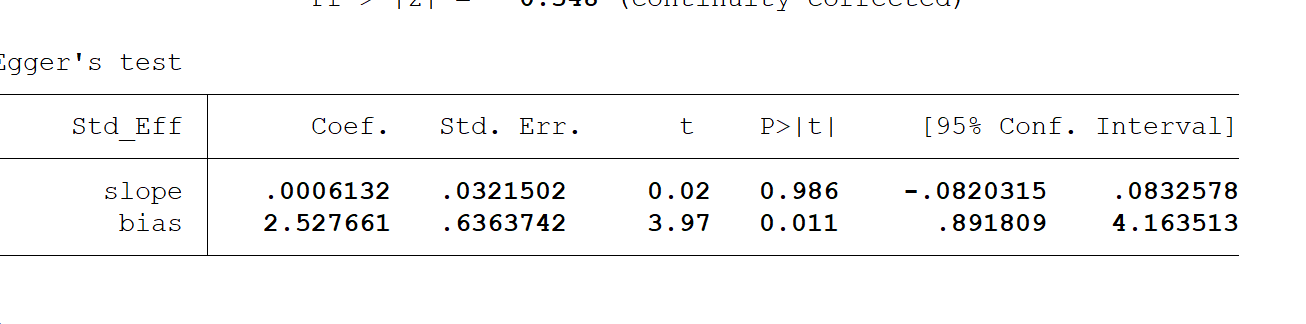


Figure S11 egger’s test of TyG-Q4 Index and Sarcopenia


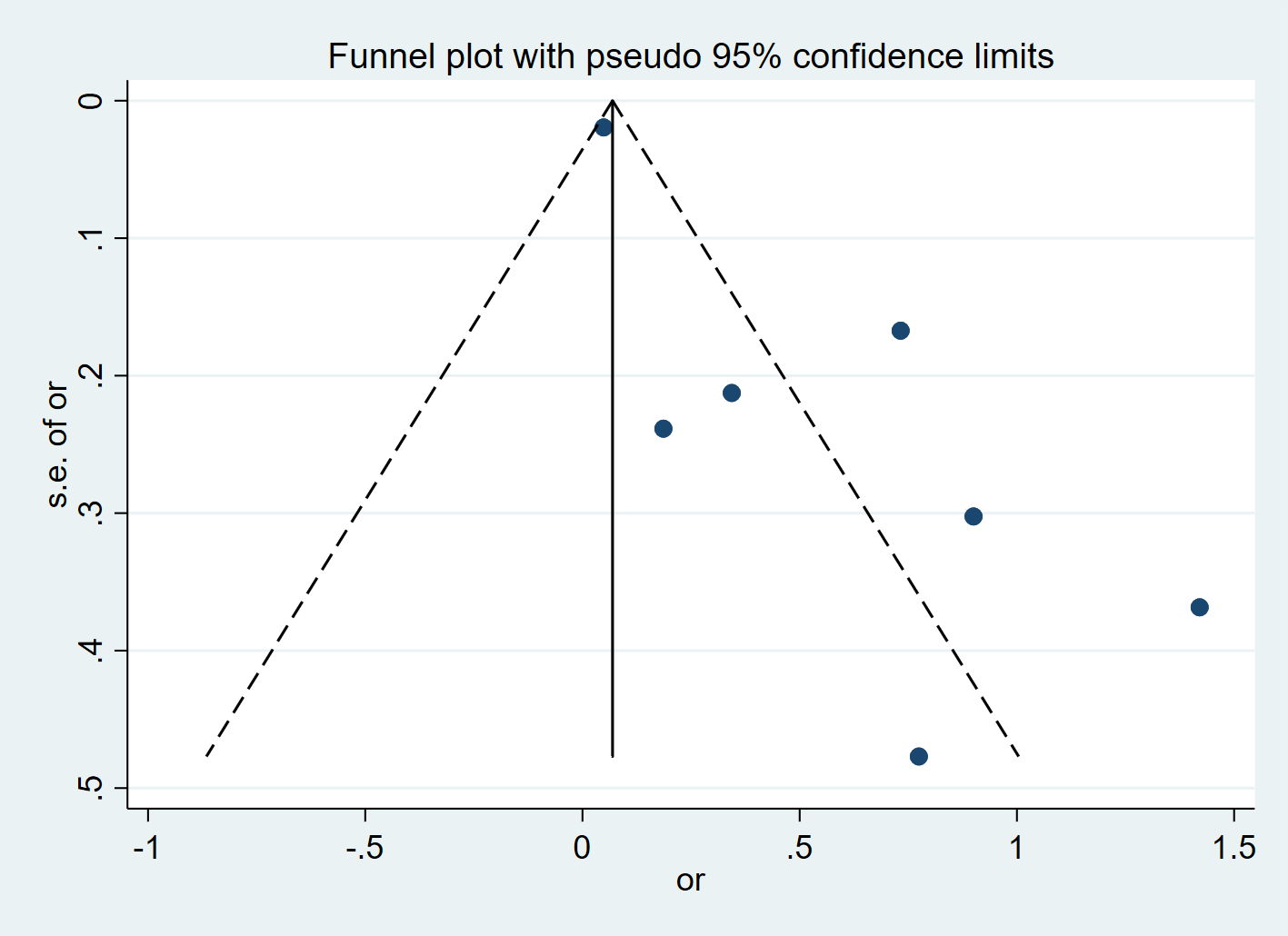


Figure S12 Meta-analysis funnel plot of TyG-Q4 Index and Sarcopenia


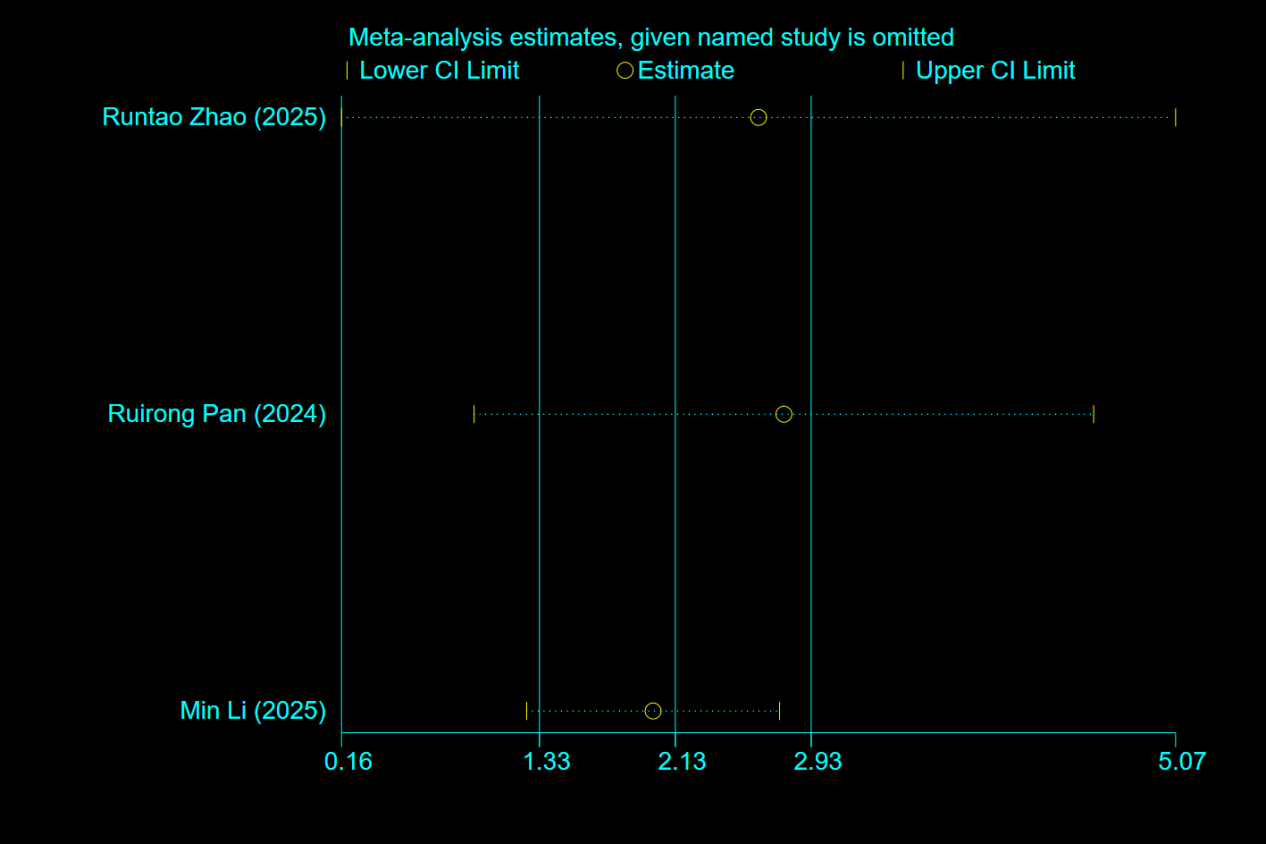


Figure S13 TyG-BMI-Q2 and sarcopenia sensitivity analysis


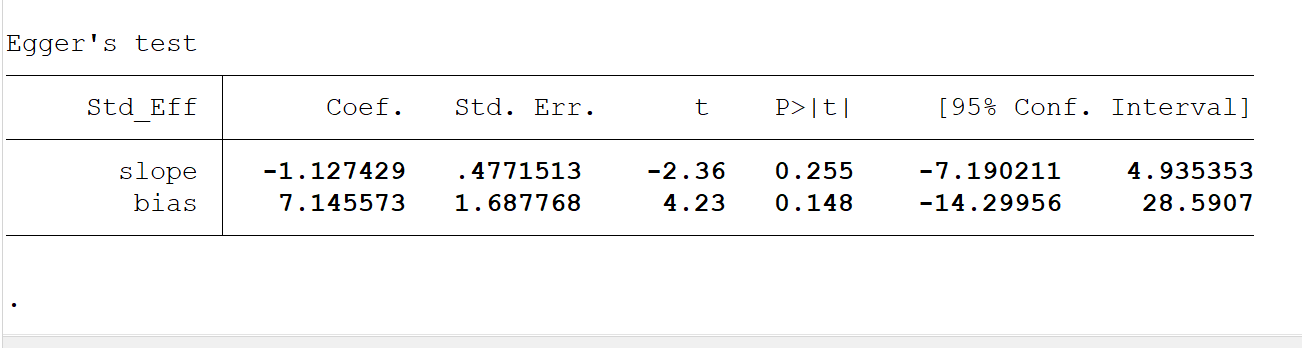


Figure S14 egger’s test of TyG-BMI-Q2 Index and Sarcopenia


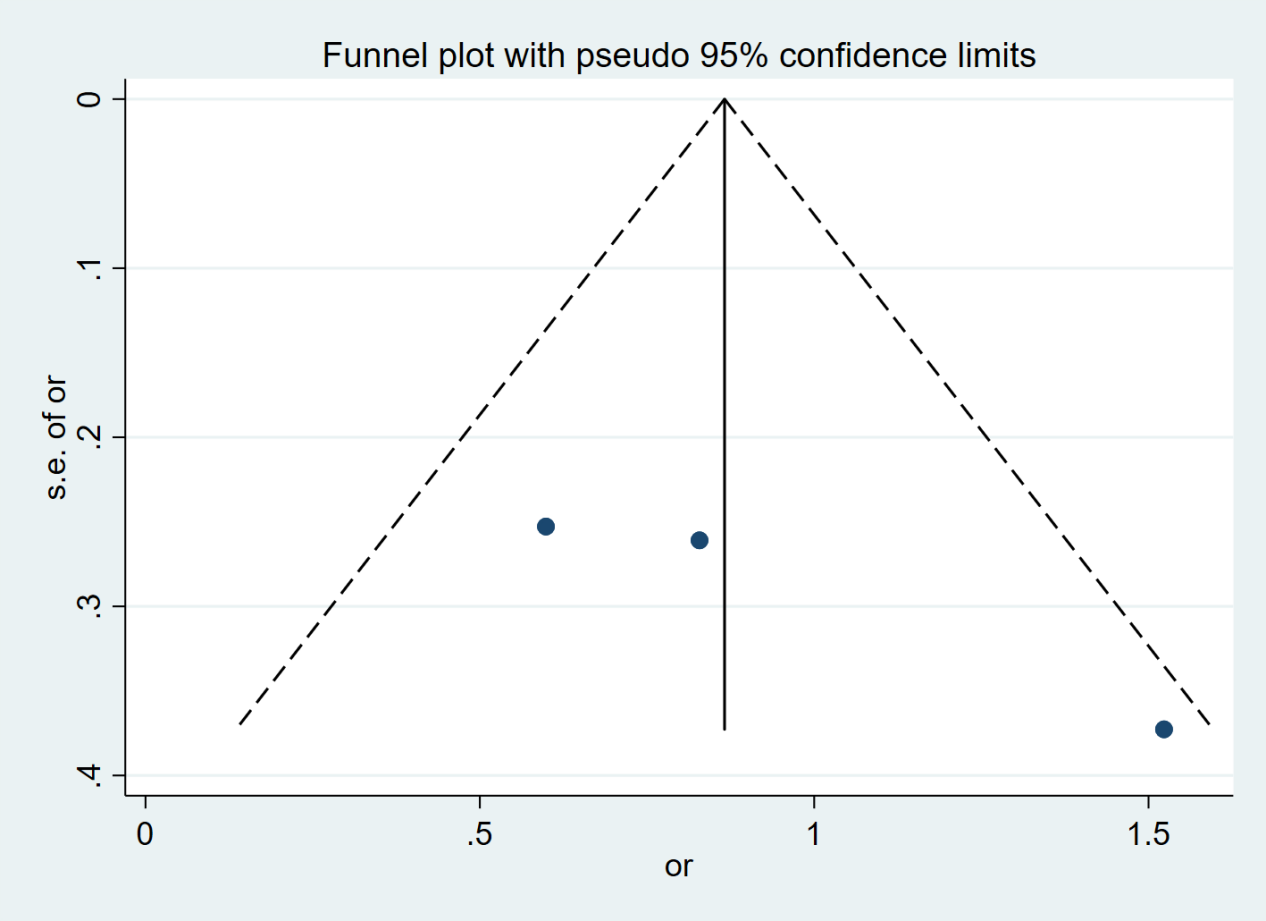


Figure S15 Meta-analysis funnel plot of TyG-BMI-Q2 Index and Sarcopenia


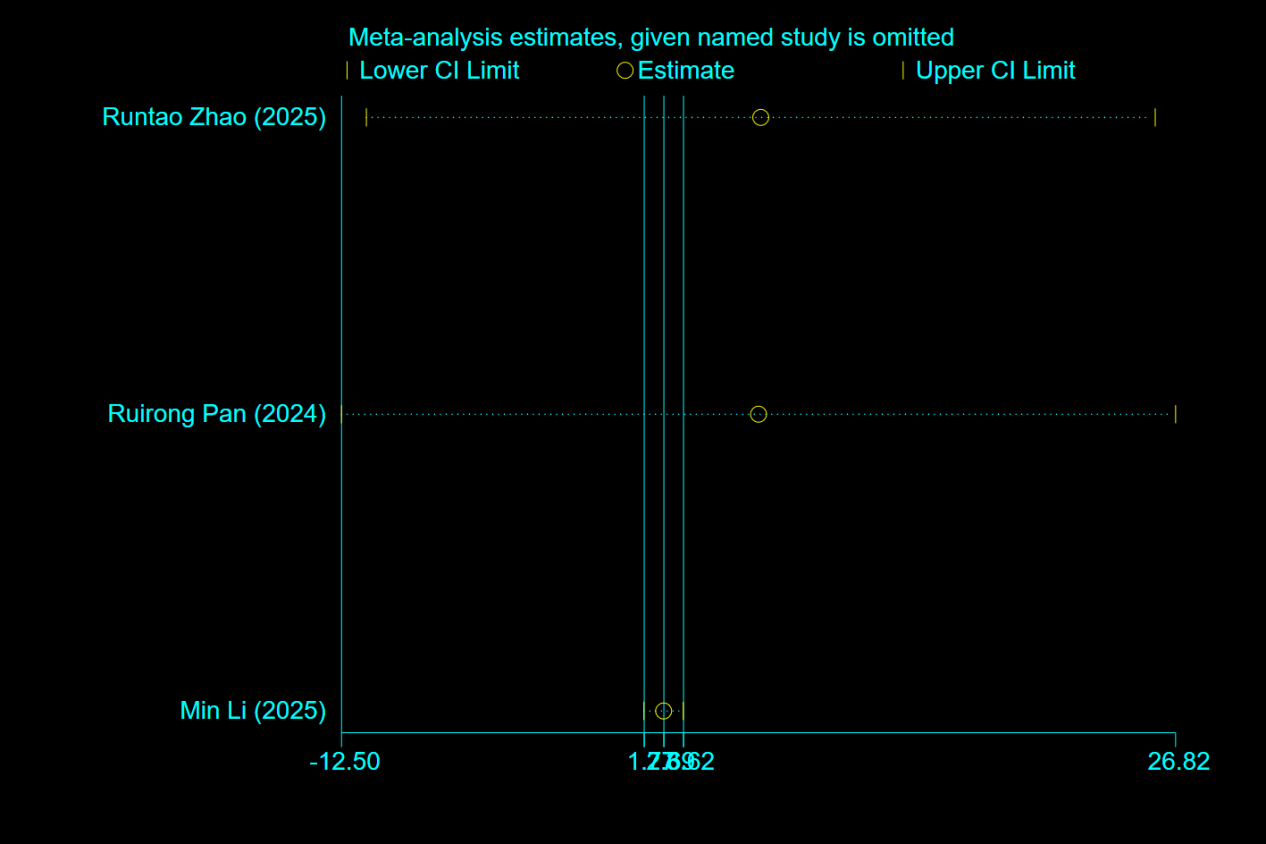


Figure S16 TyG-BMI-Q3 and sarcopenia sensitivity analysis


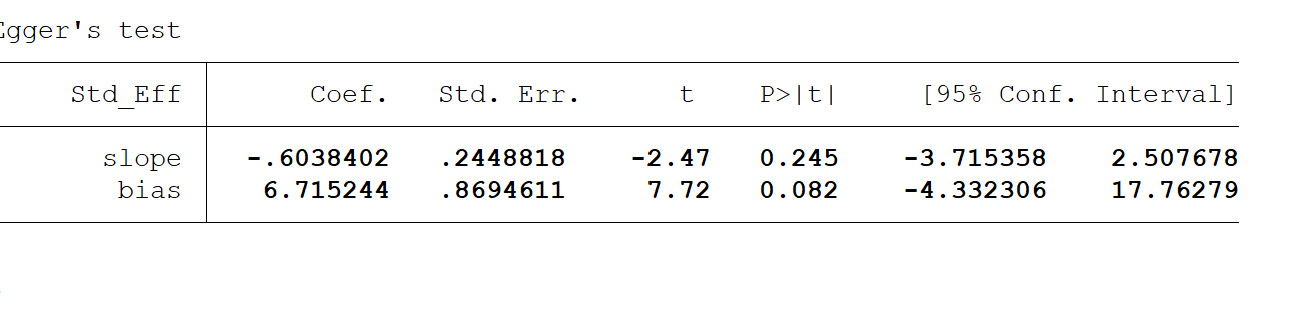


Figure S17 egger’s test of TyG-BMI-Q3 Index and Sarcopenia


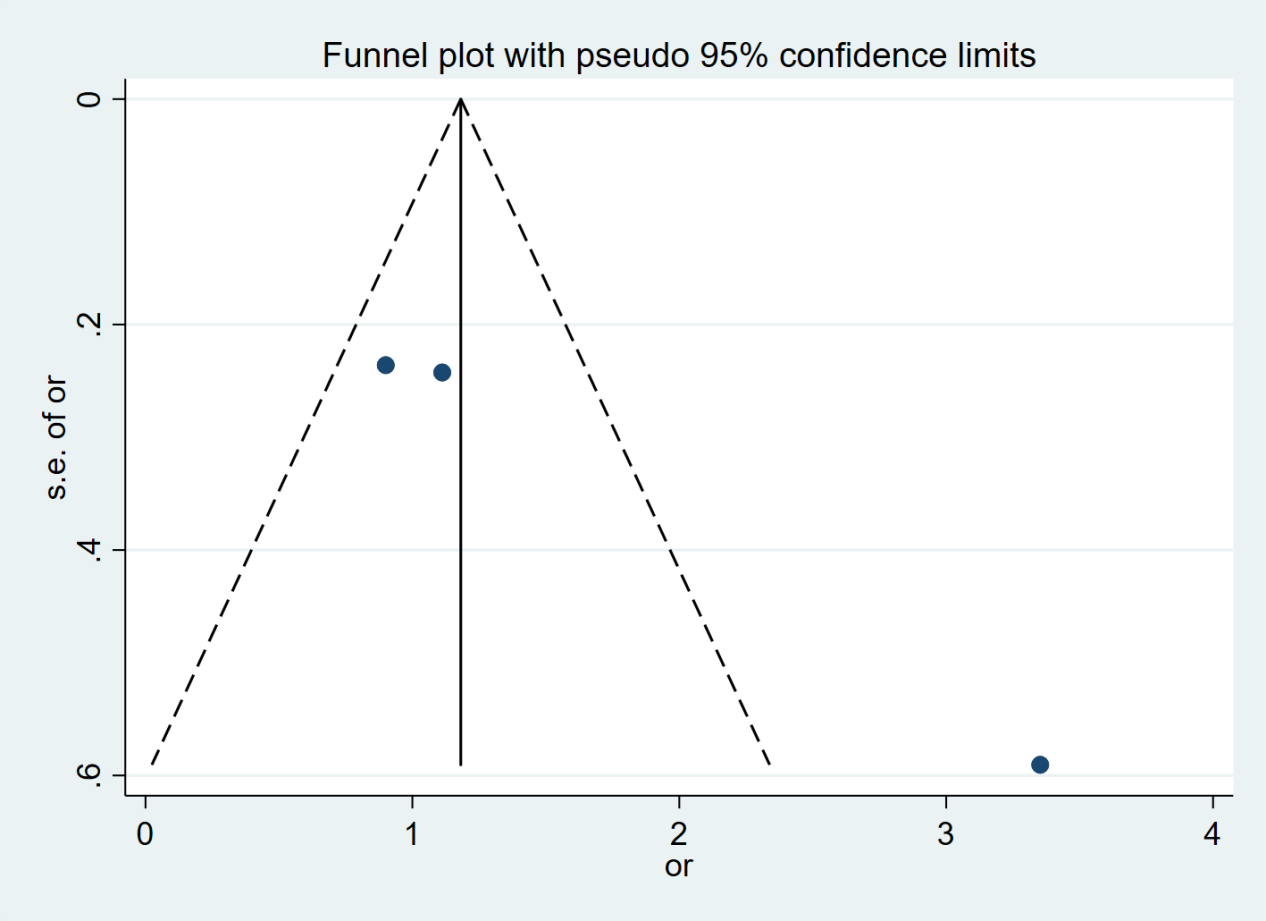


Figure S18 Meta-analysis funnel plot of TyG-BMI-Q3 Index and Sarcopenia


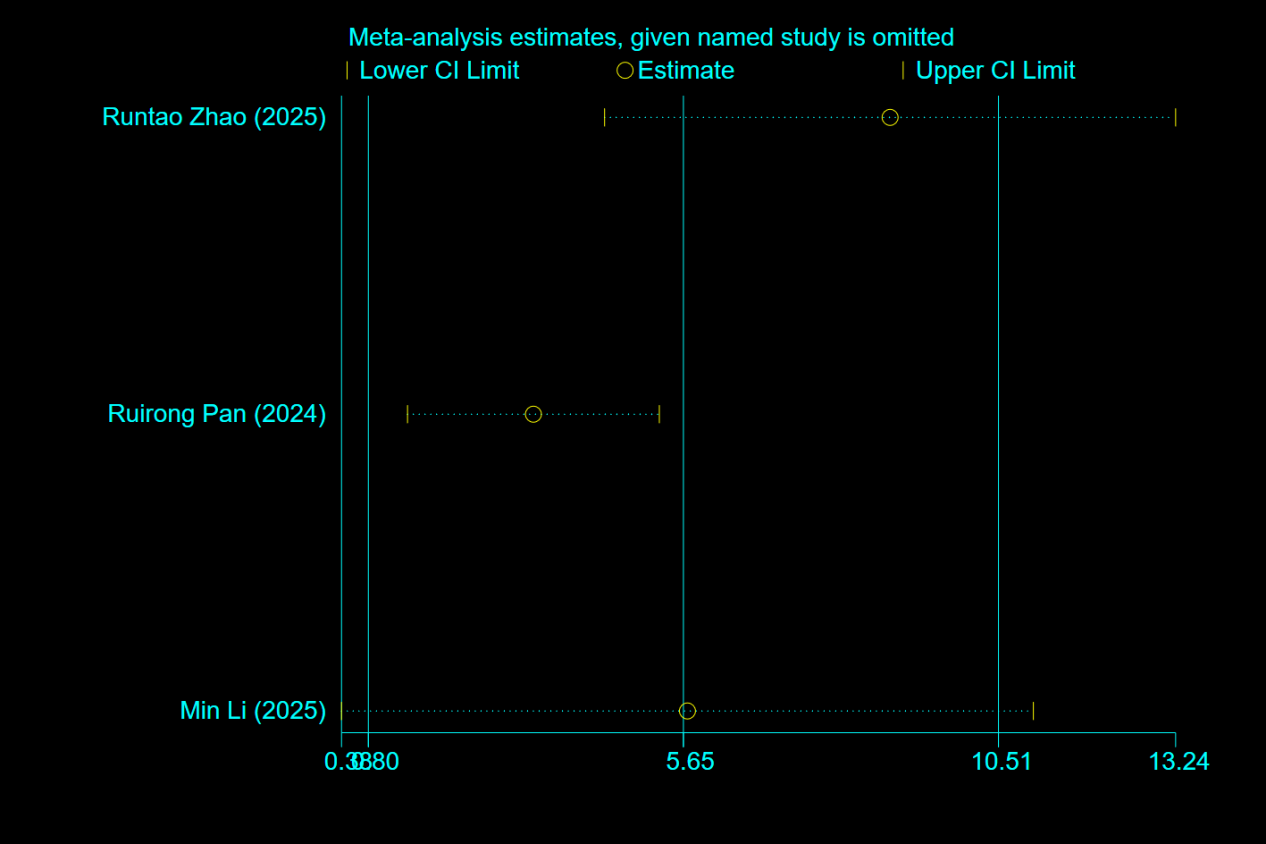


Figure S19 TyG-BMI-Q4 and sarcopenia sensitivity analysis


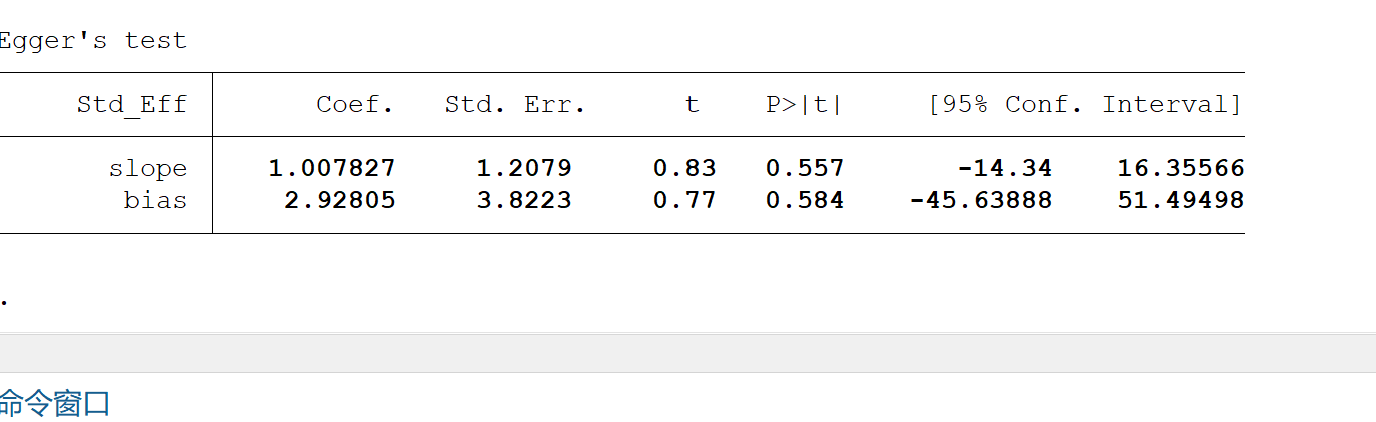


Figure S20 egger’s test of TyG-BMI-Q4 Index and Sarcopenia


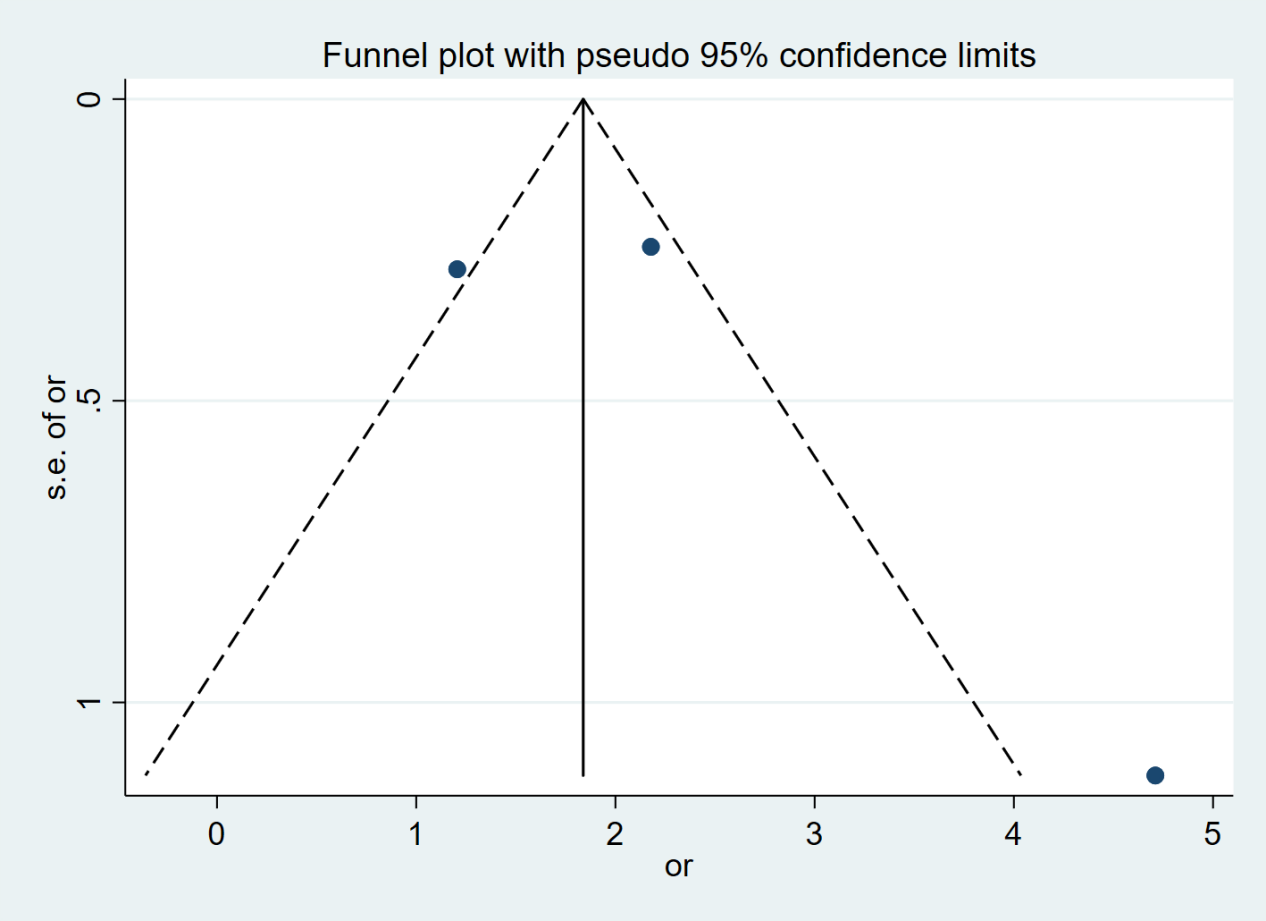


Figure S21 Meta-analysis funnel plot of TyG-BMI-Q4 Index and Sarcopenia


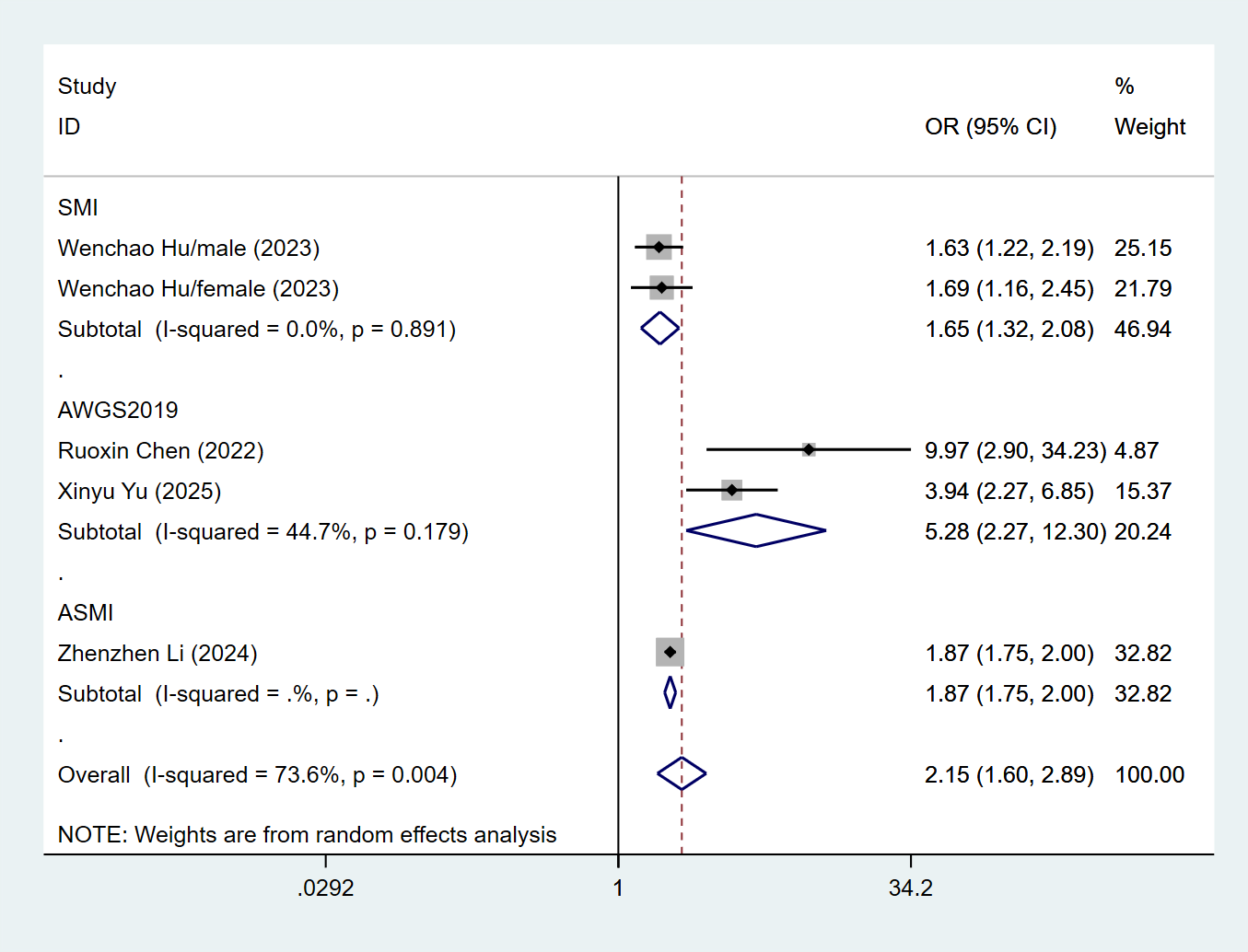


Figure S22 Subgroup analysis by sarcopenia diagnostic approach


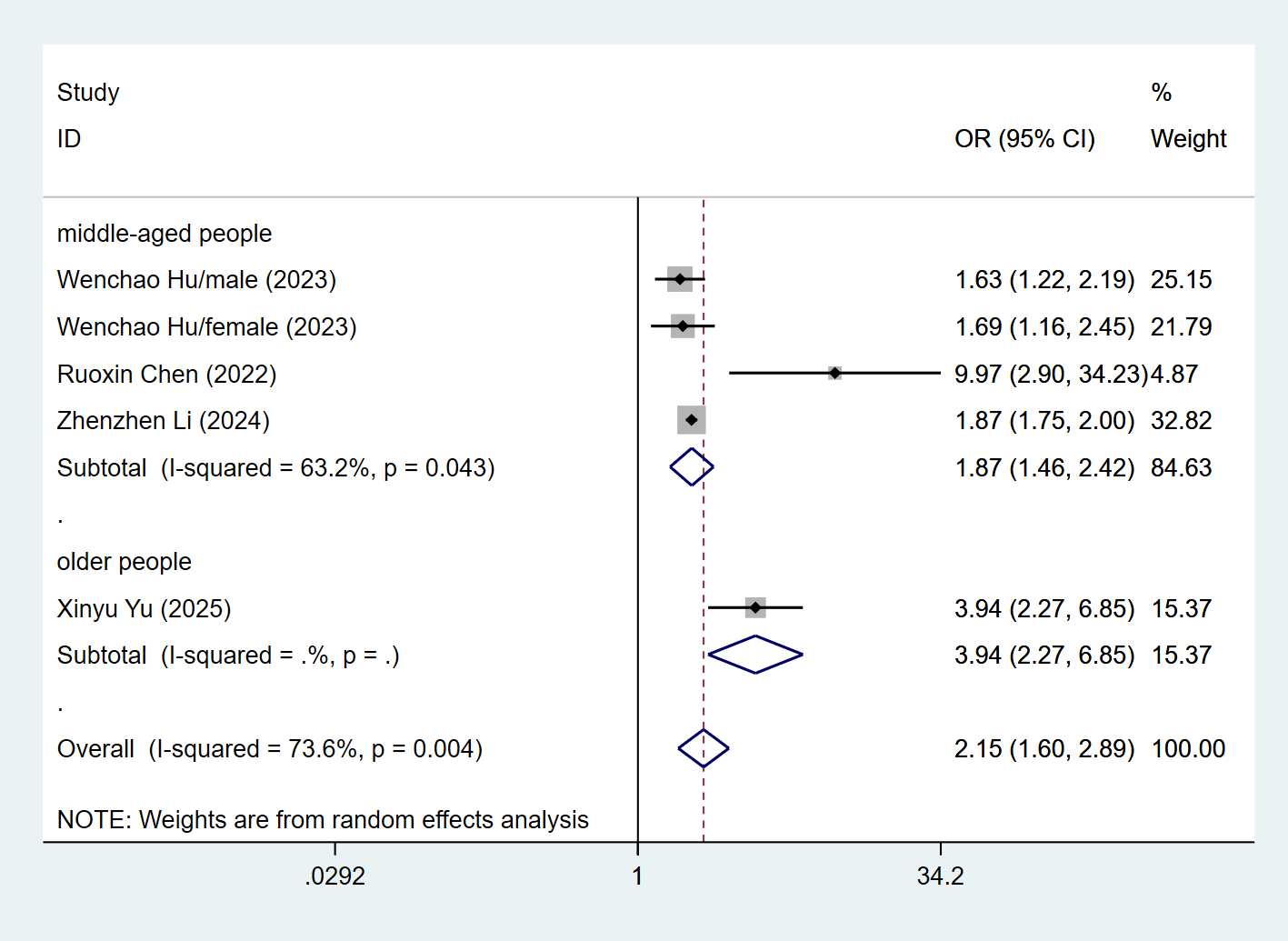


Figure S23 Subgroup analysis bySubgroup analysis through population studies


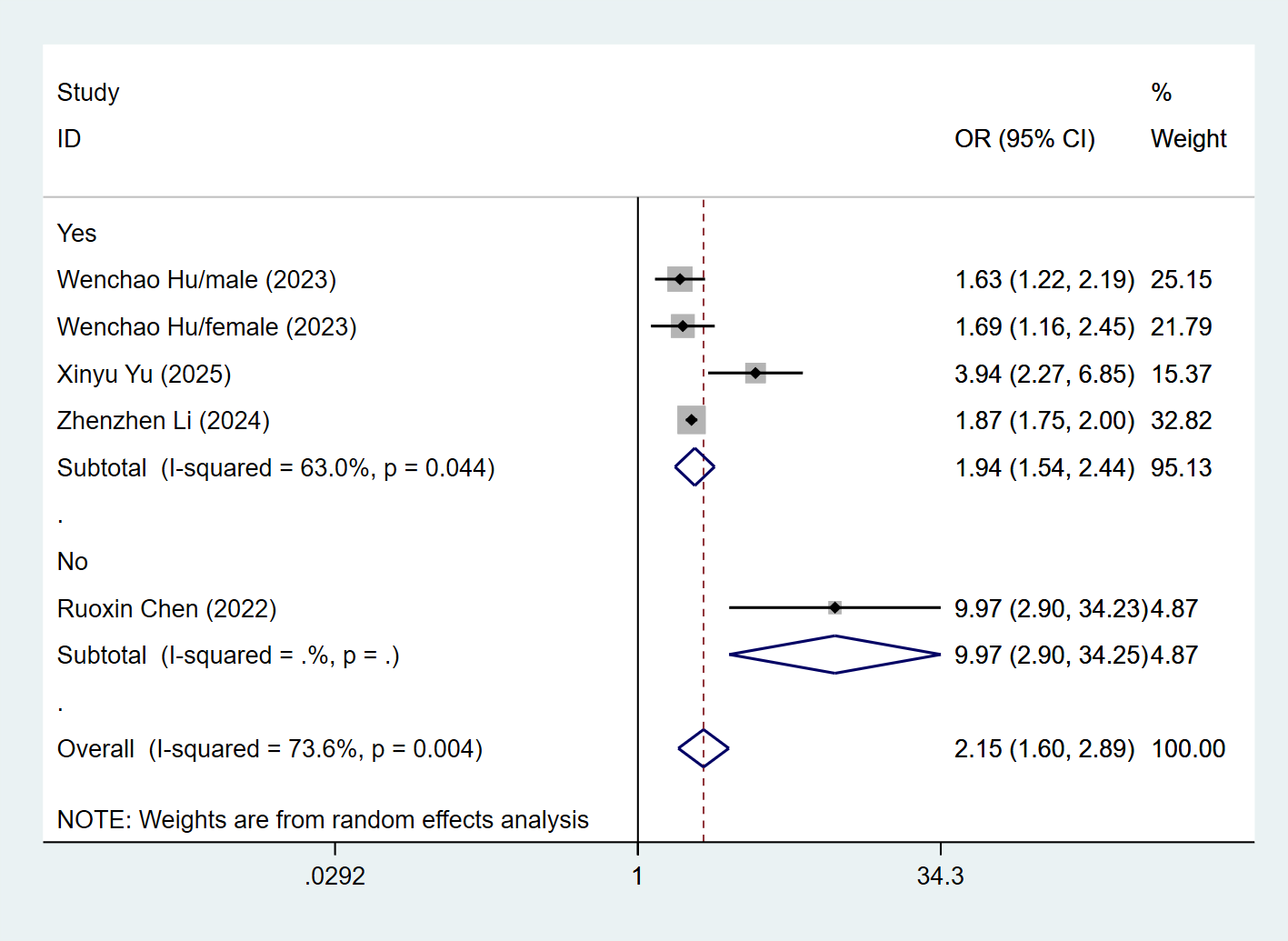


Figure S24 Subgroup analysis by comorbidities


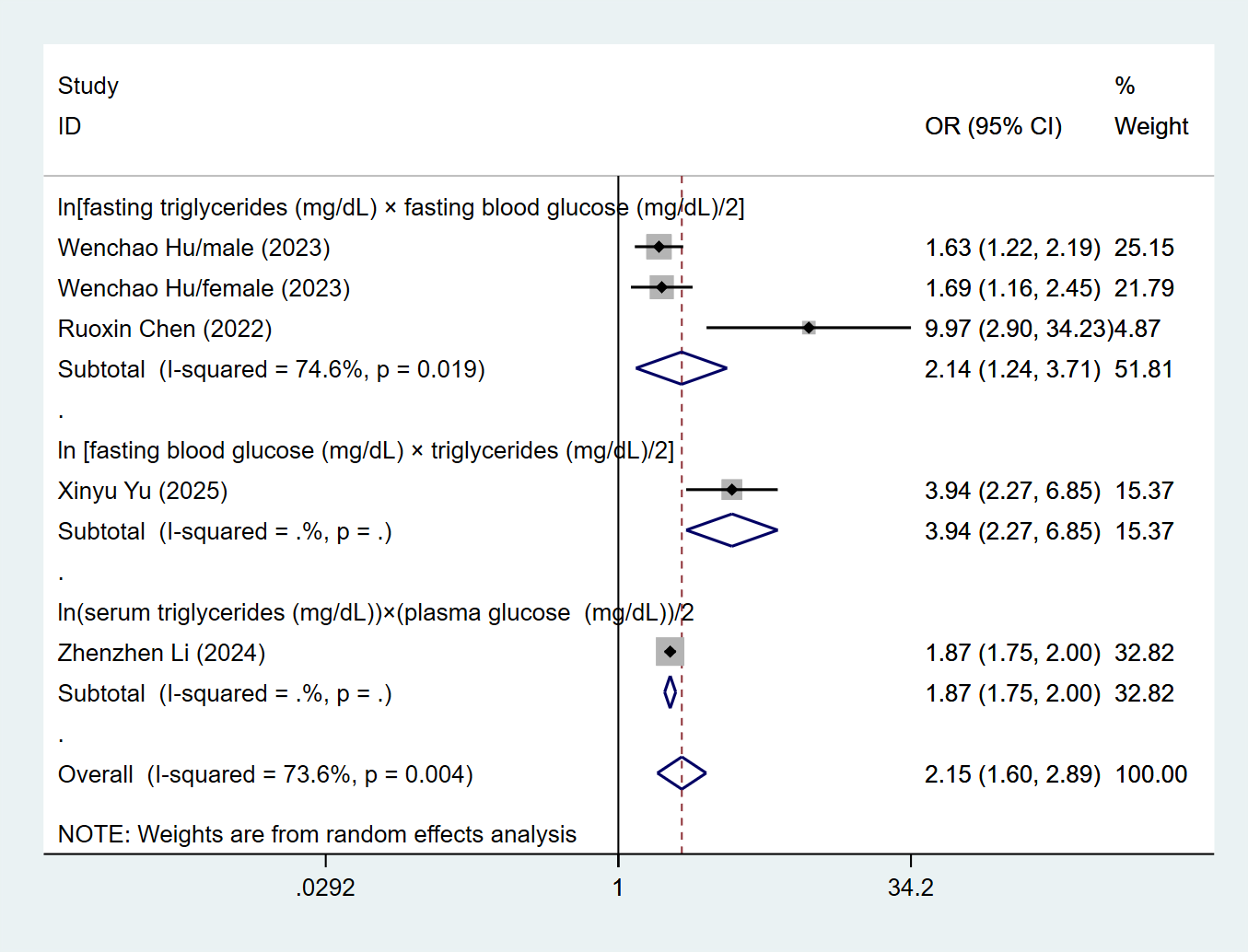


Figure S25 Subgroup analysis by TyG measurement method


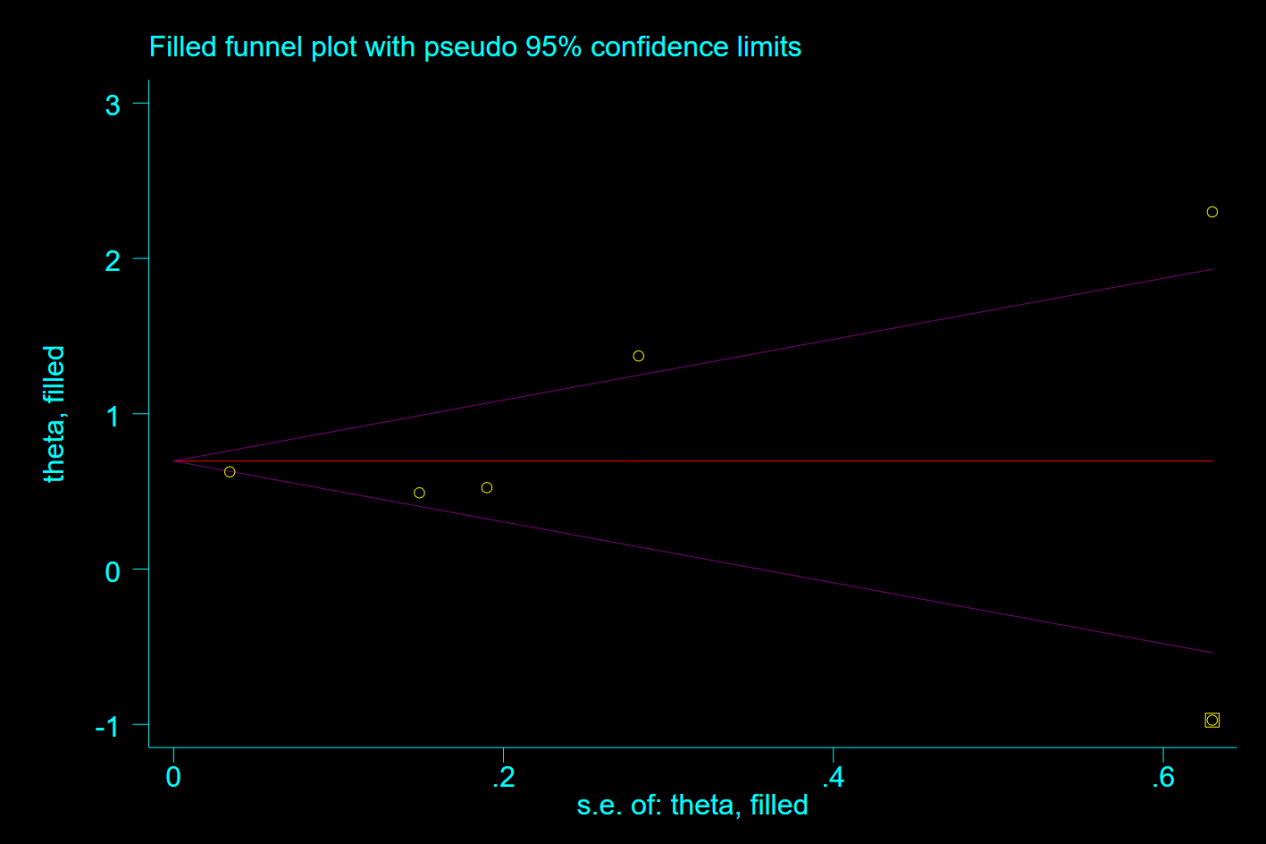


Figure S26 The trim and fill funnel plot of Total TyG Index and Sarcopenia


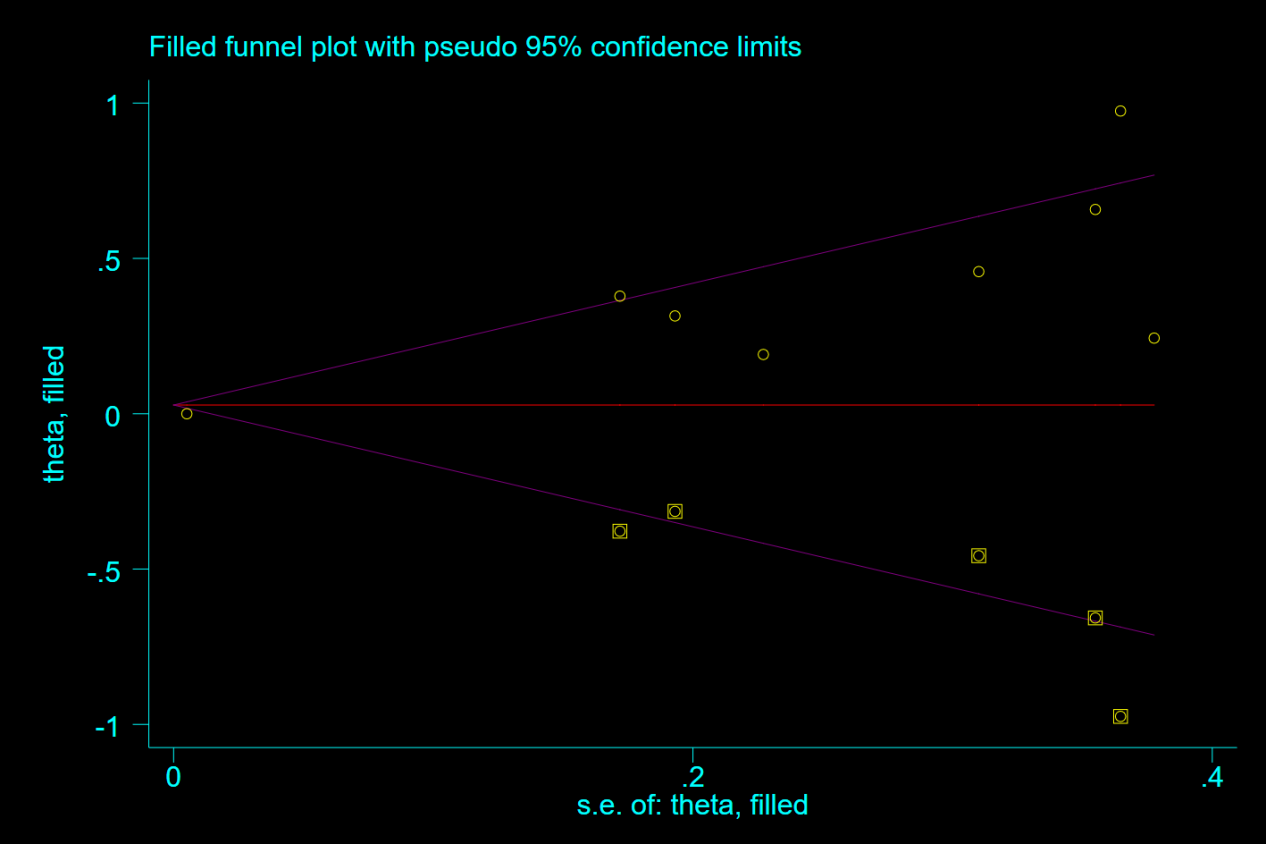


Figure S27 The trim and fill funnel plot of TyG-Q2 and Sarcopenia


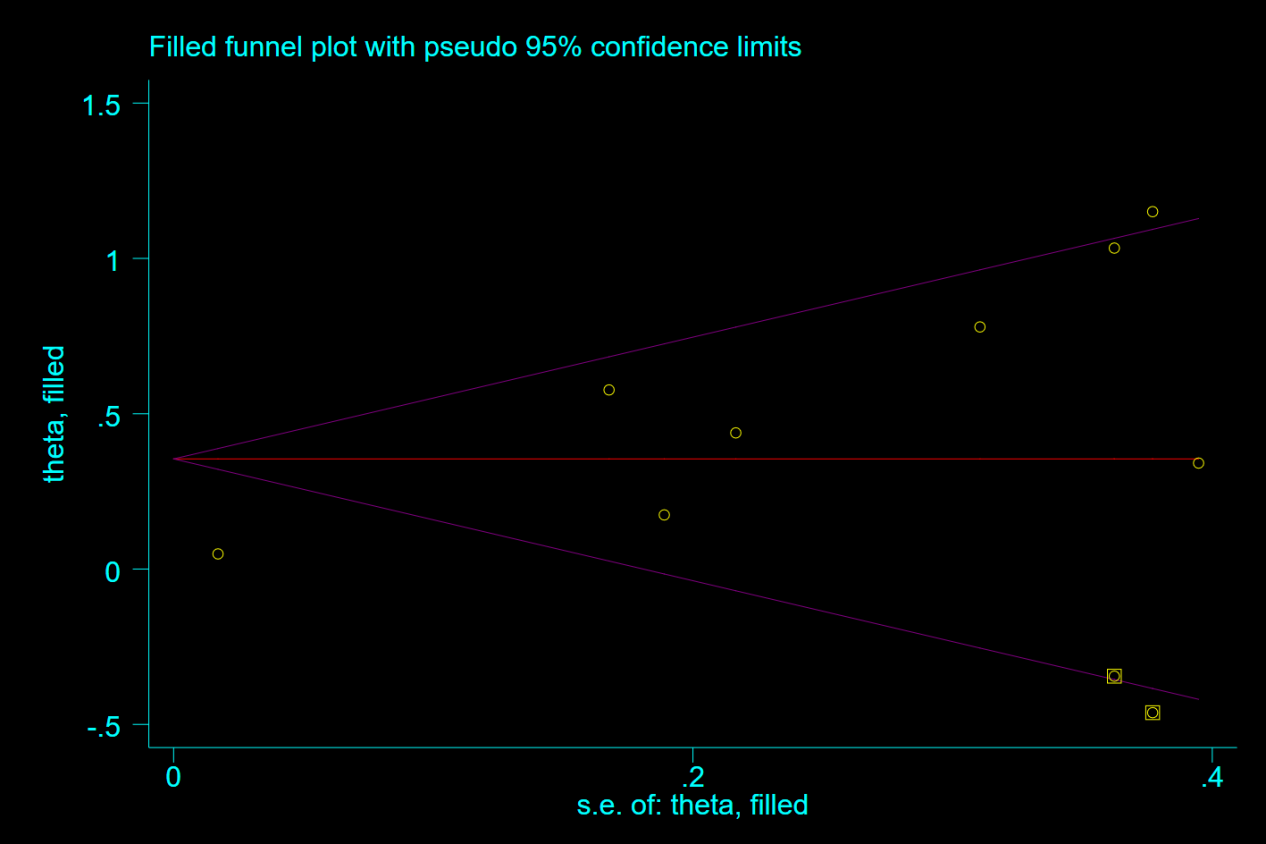


Figure S28 The trim and fill funnel plot of TyG-Q3 and Sarcopenia


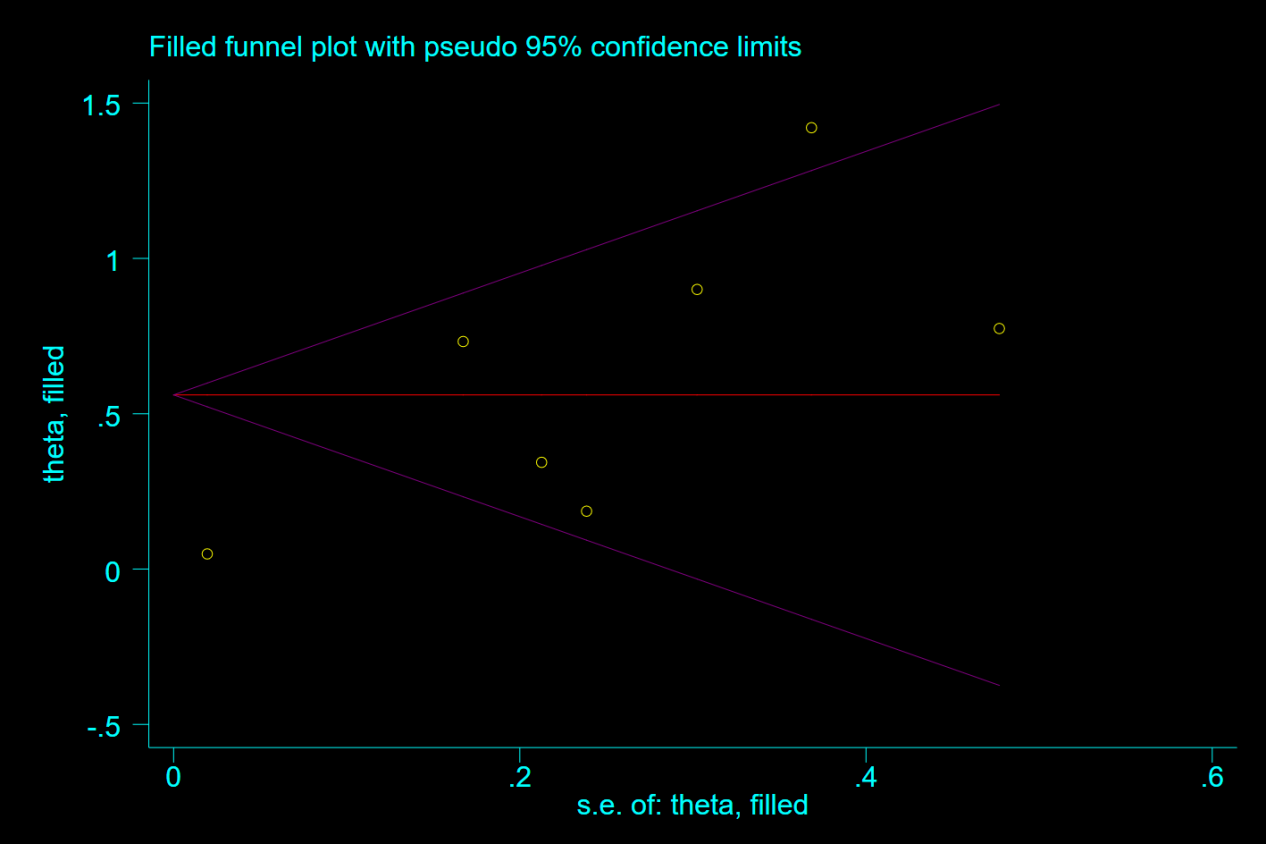


Figure S29 The trim and fill funnel plot of TyG-Q4 and Sarcopenia


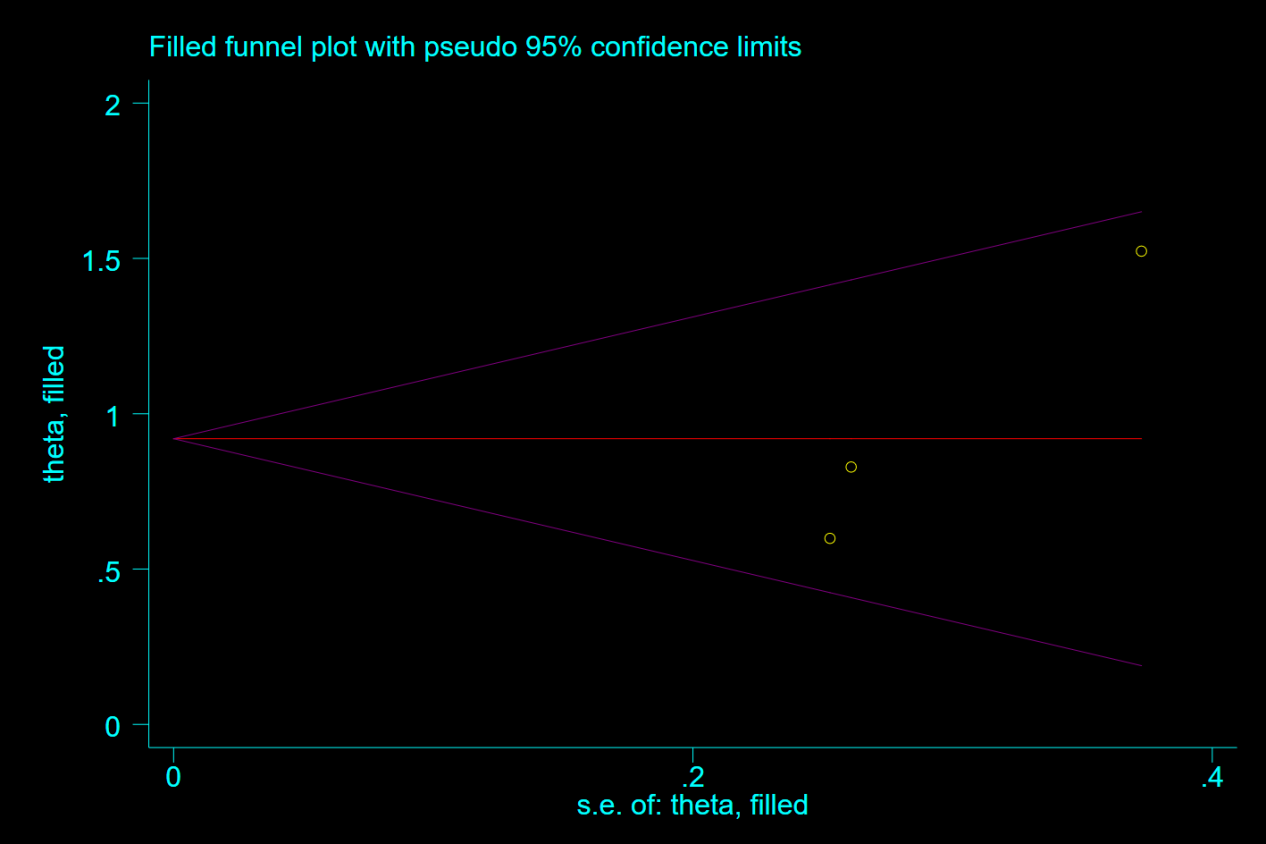


Figure S30 The trim and fill funnel plot of TyG-BMI-Q2 and Sarcopenia


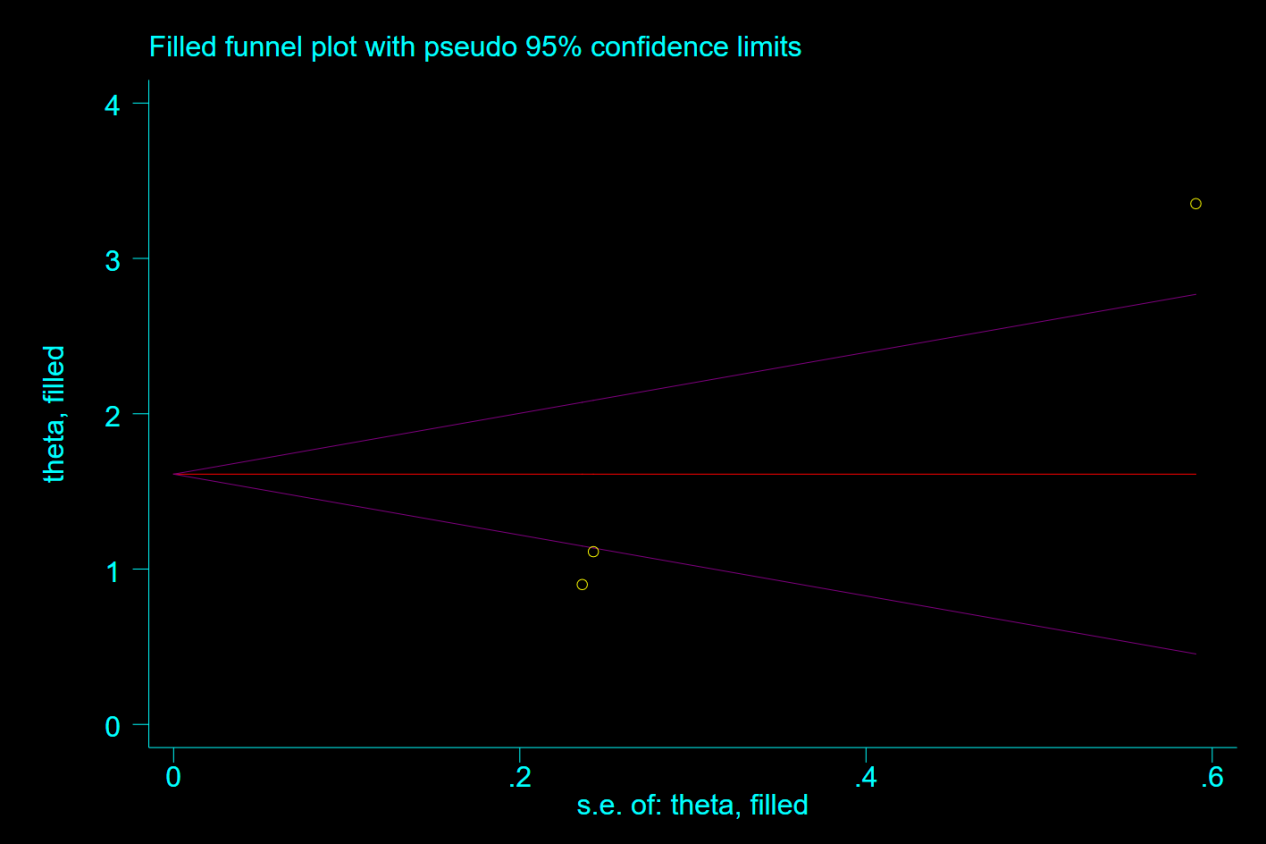


Figure S31 The trim and fill funnel plot of TyG-BMI-Q3 and Sarcopenia


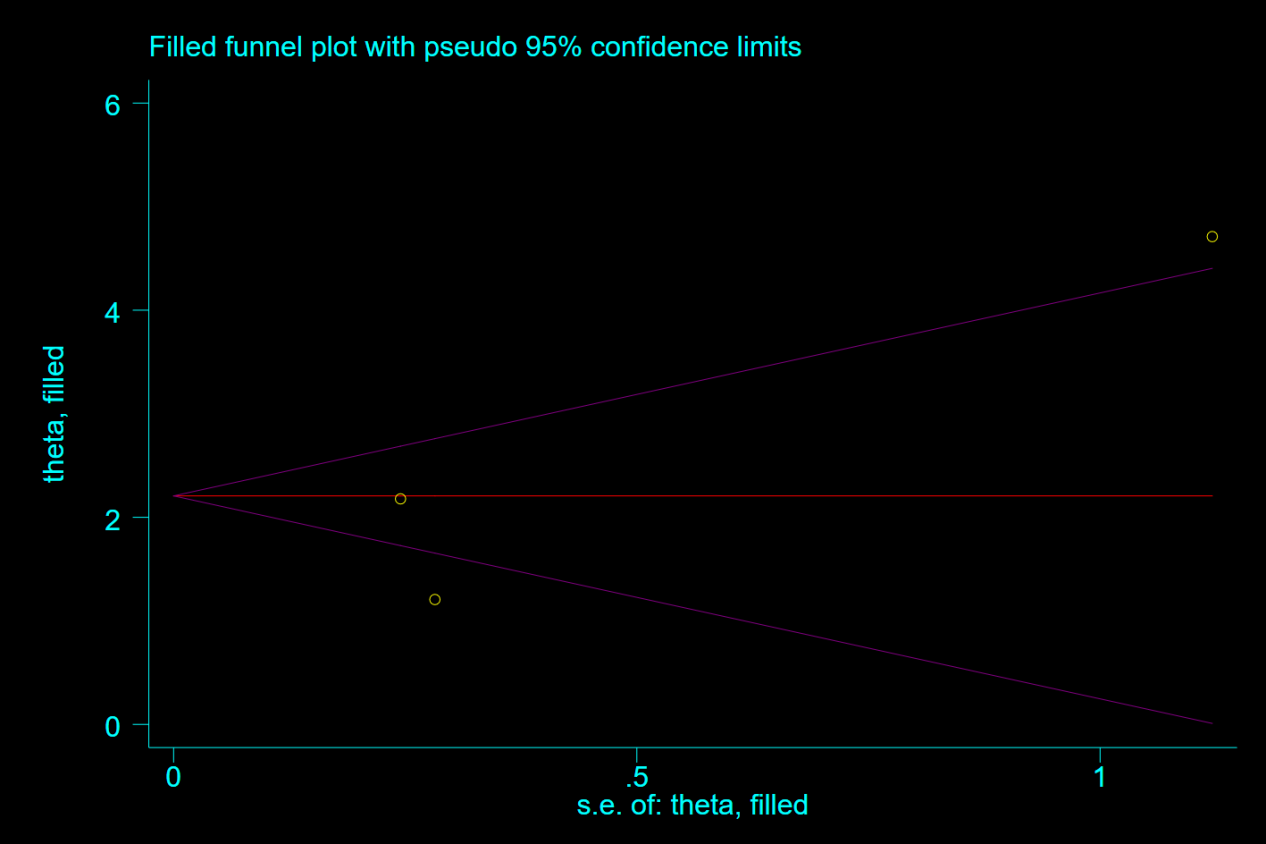


Figure S32 The trim and fill funnel plot of TyG-BMI-Q4 and Sarcopenia


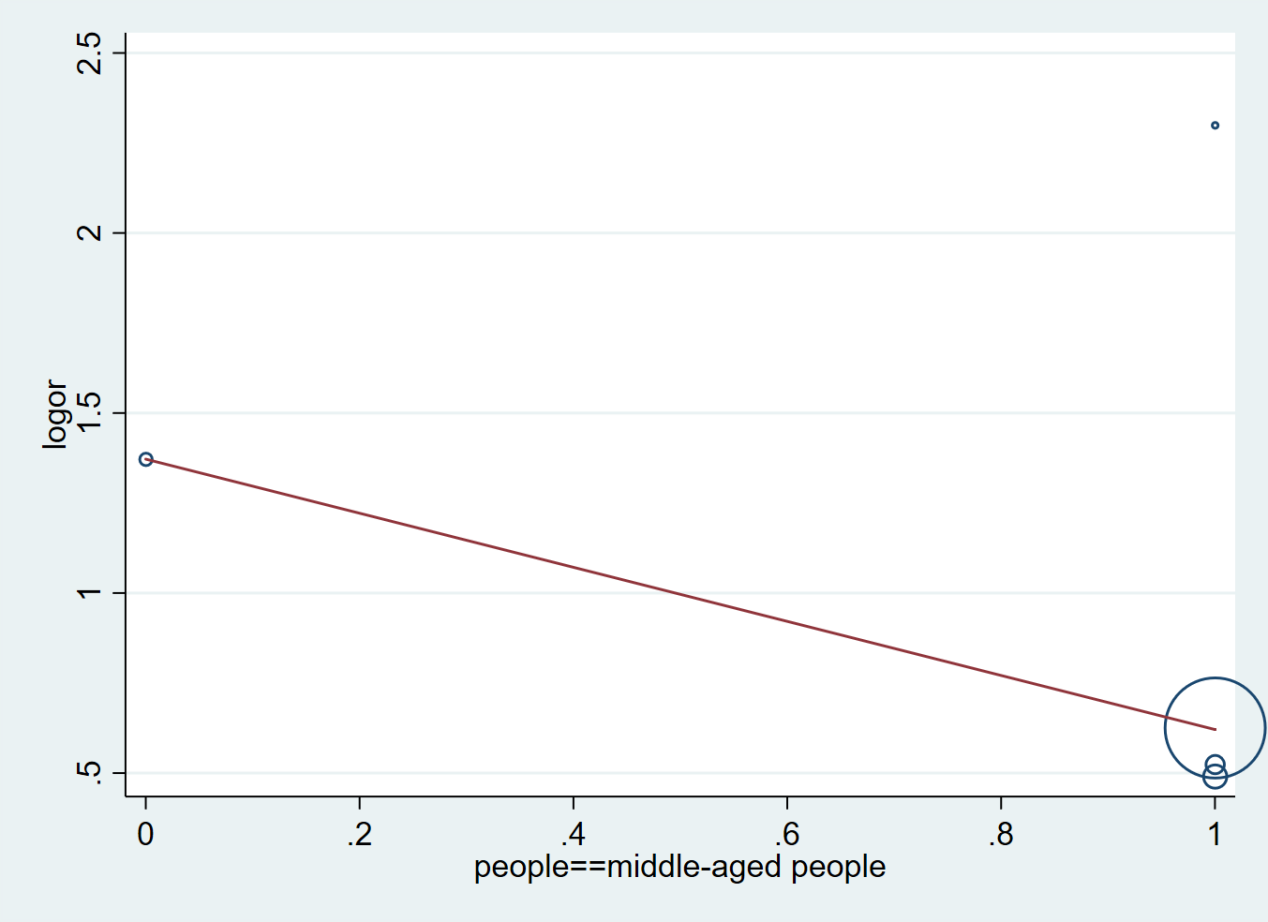


Figure S33 Meta-regression analysis based on the study population(middle-aged people)


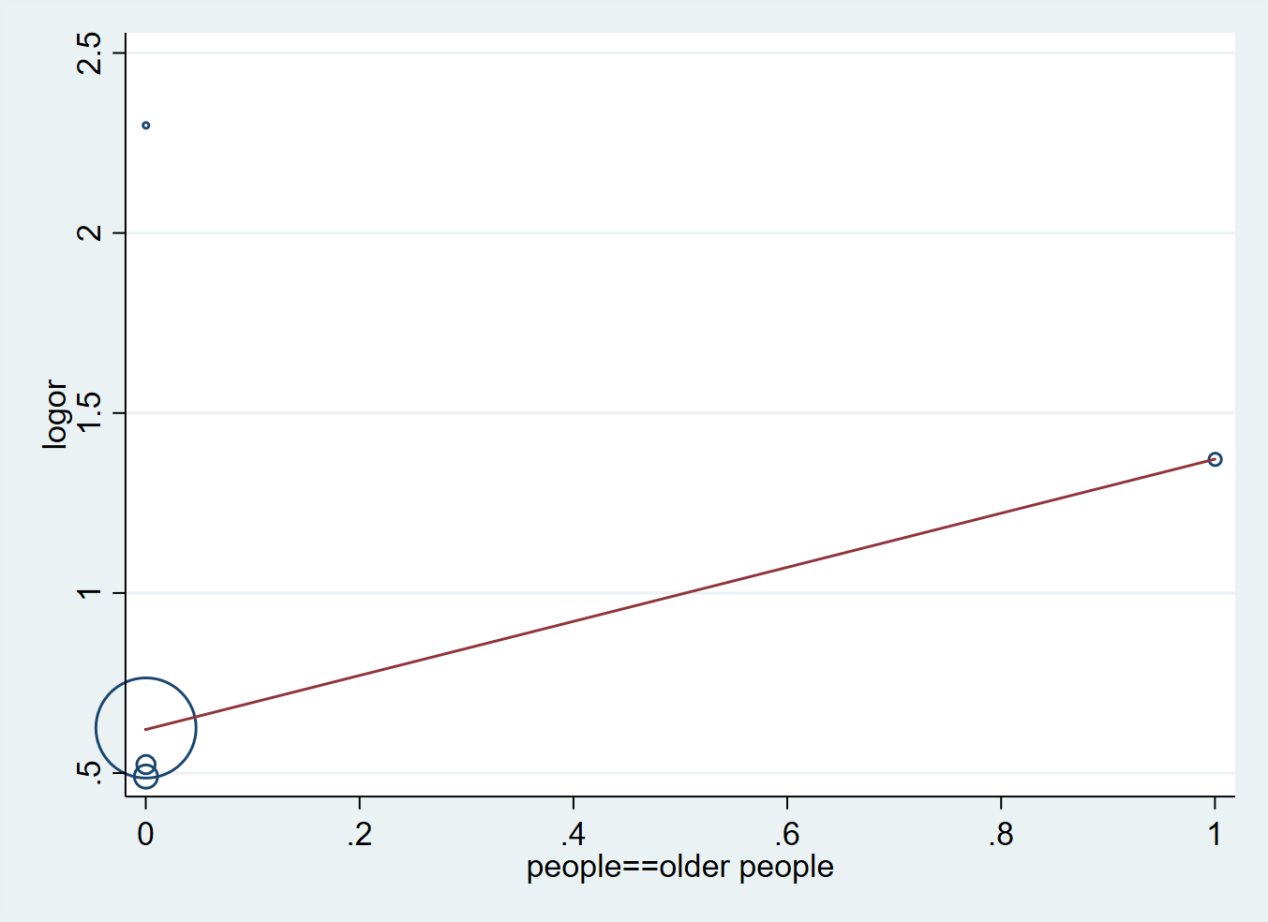


Figure S34 Meta-regression analysis based on the study population(older people)


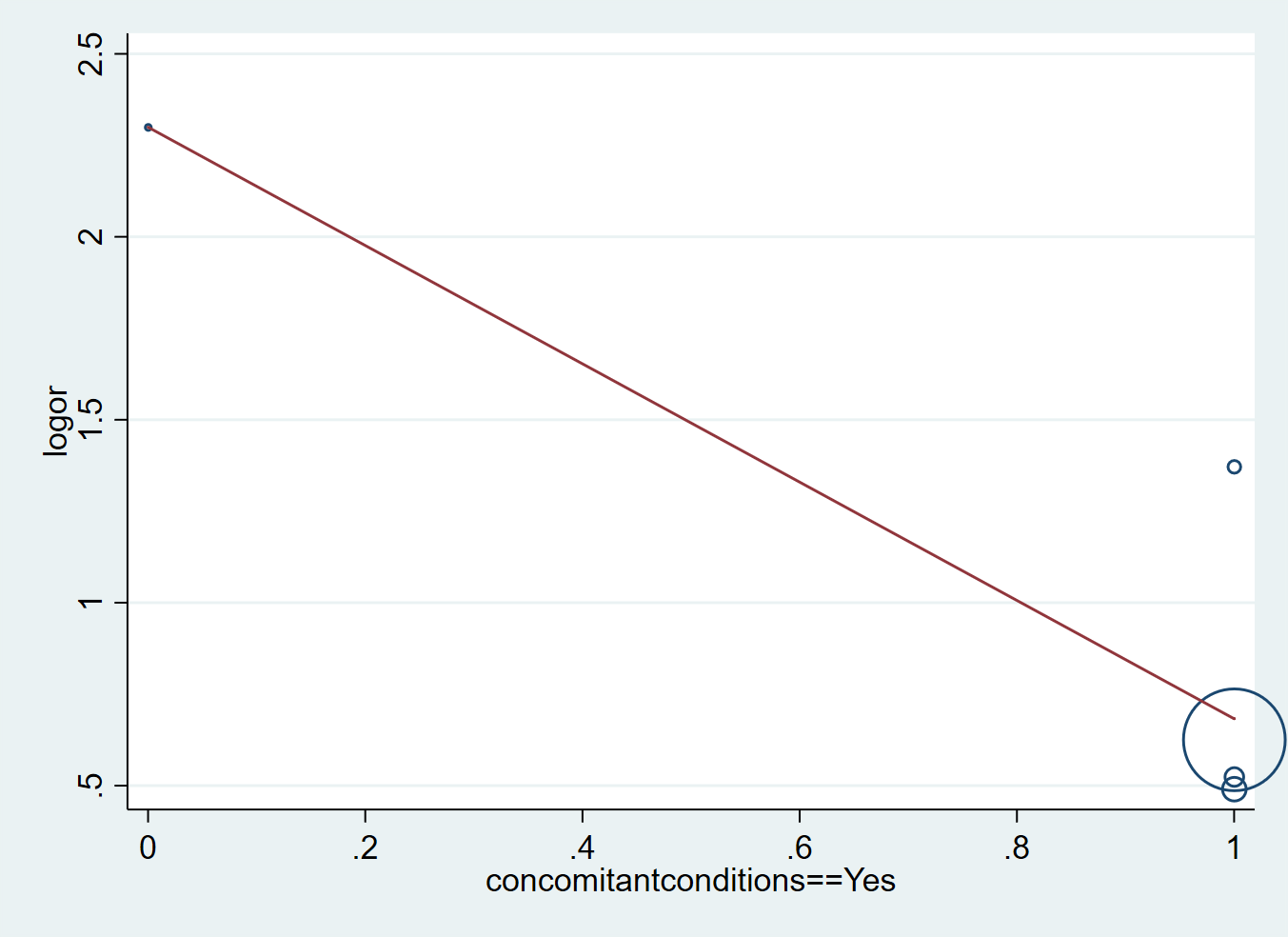


Figure S35 Meta-regression analysis based on comorbidities(yes)


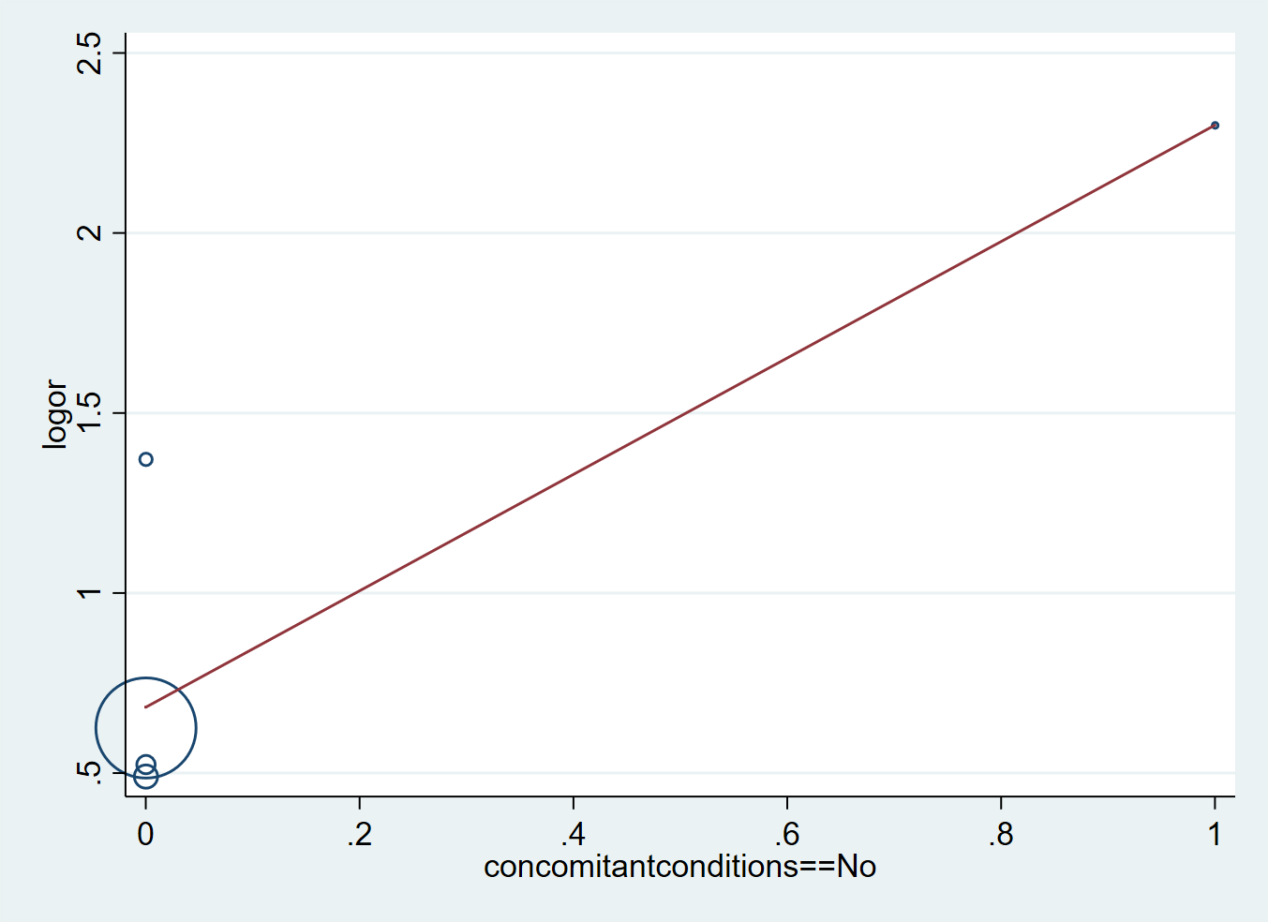


Figure S36 Meta-regression analysis based on comorbidities(No)


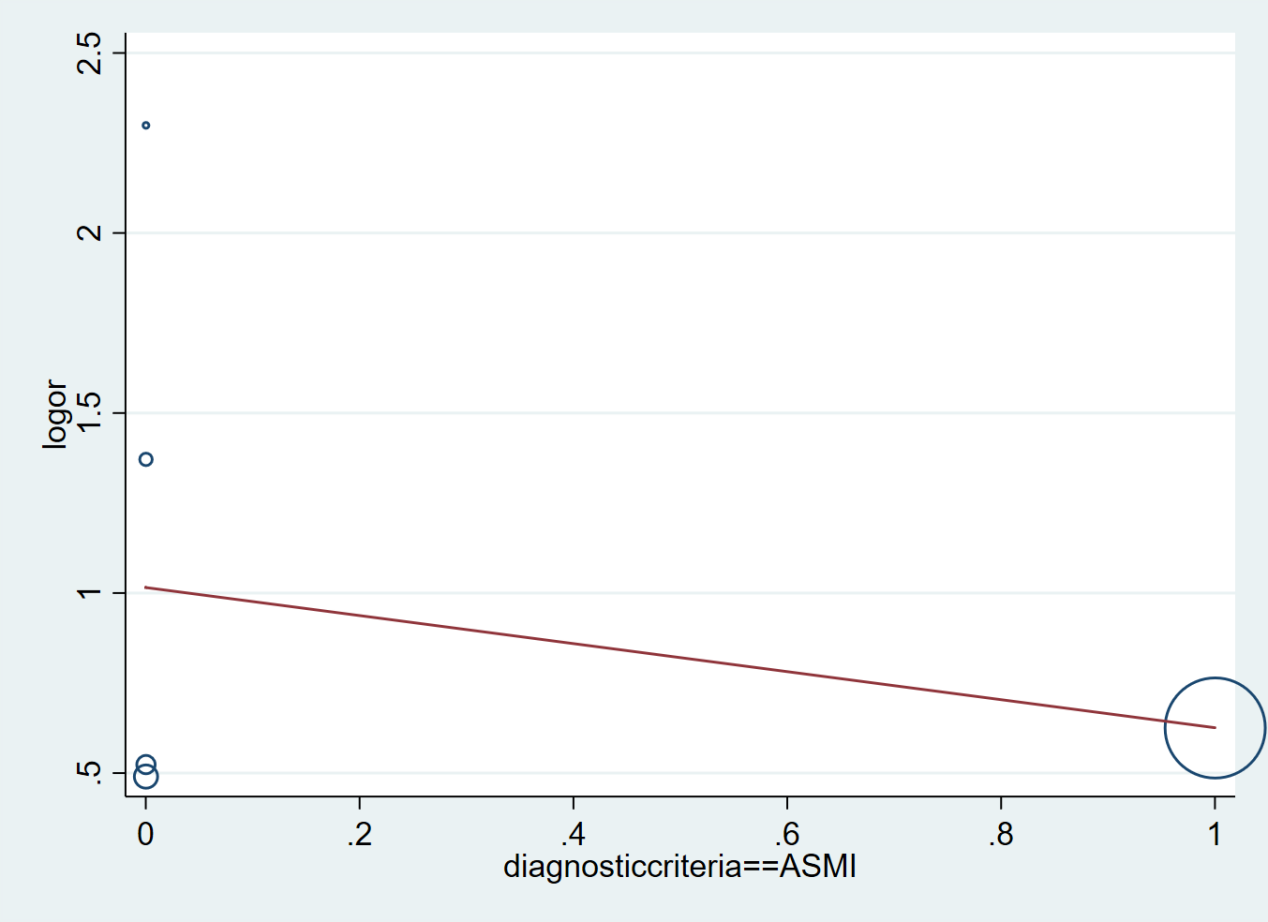


FigureS37 Meta-regression analysis based on diagnostic criteria(ASMI)


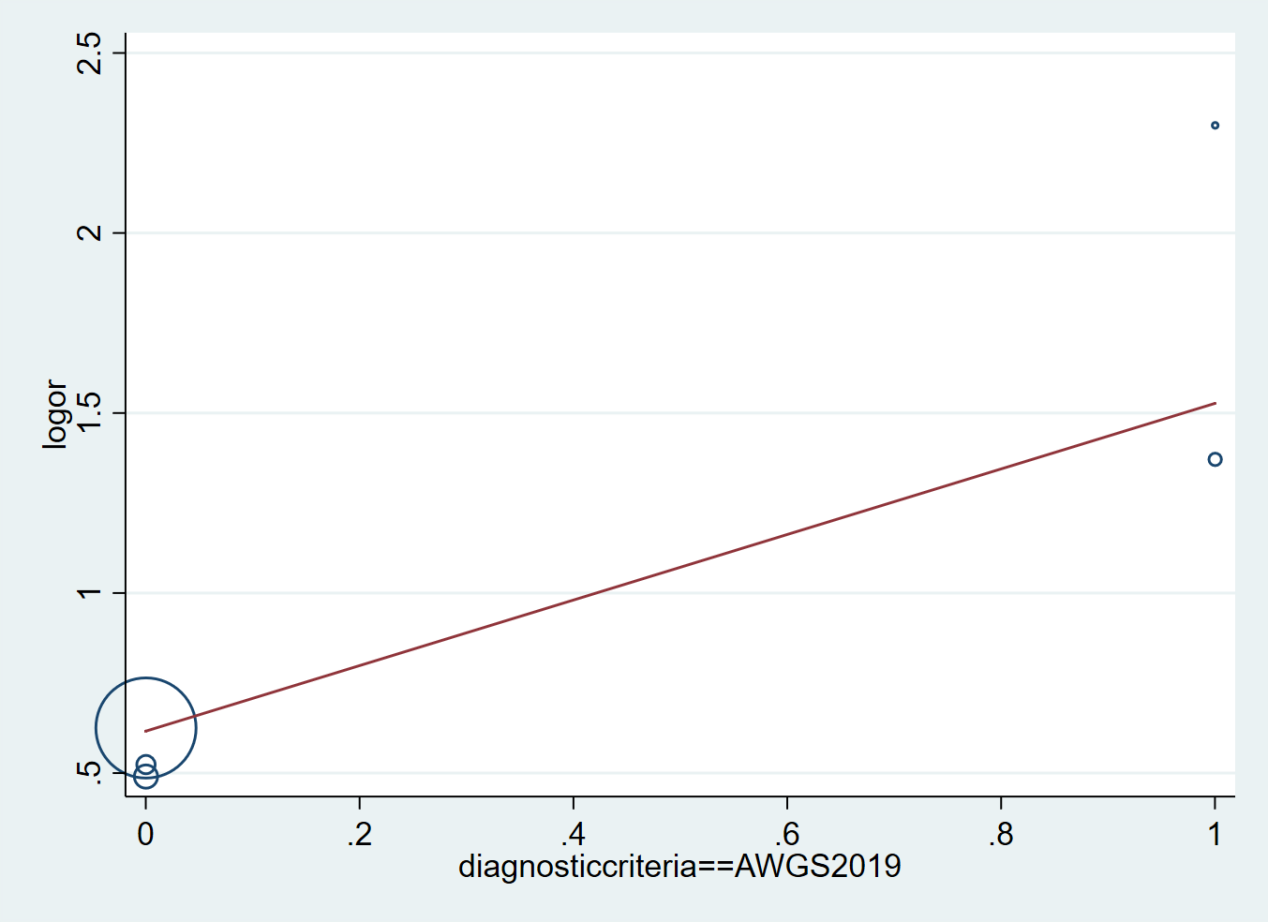


FigureS37 Meta-regression analysis based on diagnostic criteria(AWGS2019)


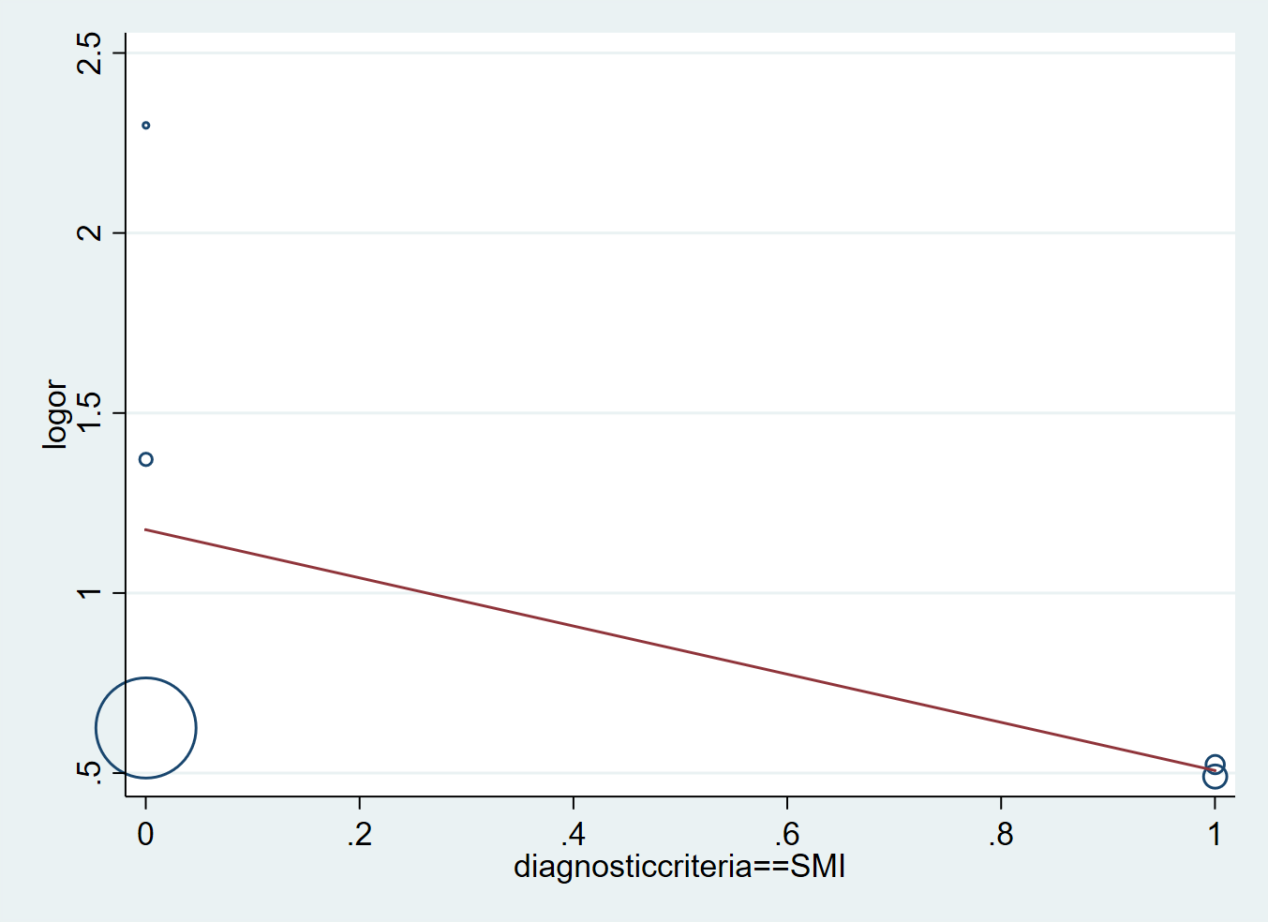


Figure S37 Meta-regression analysis based on diagnostic criteria(SMI)


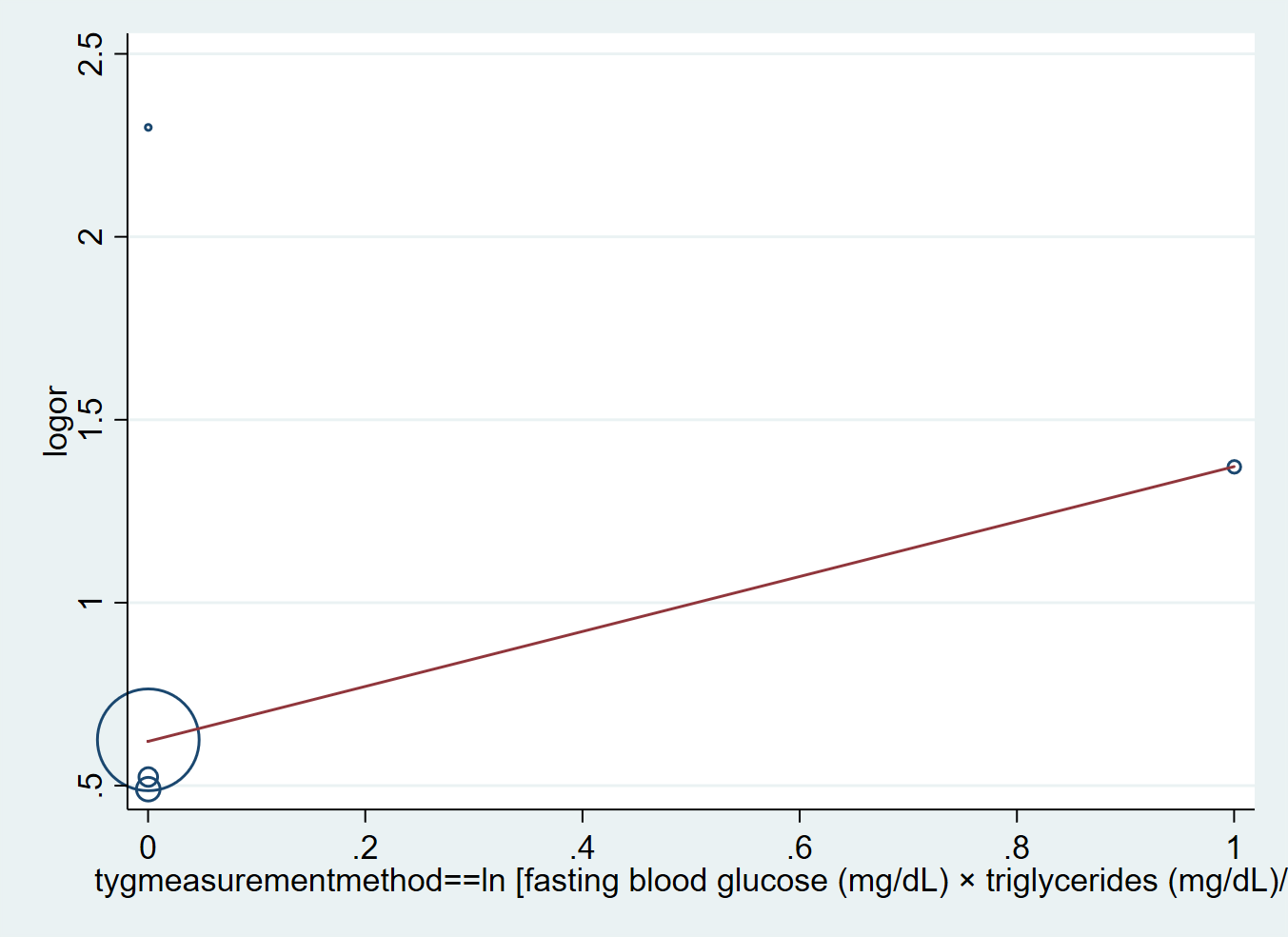


Figure S38 Meta-regression analysis based on the TyG measurement method(ln [fasting blood glucose (mg/dL) × triglycerides (mg/dL)/2])


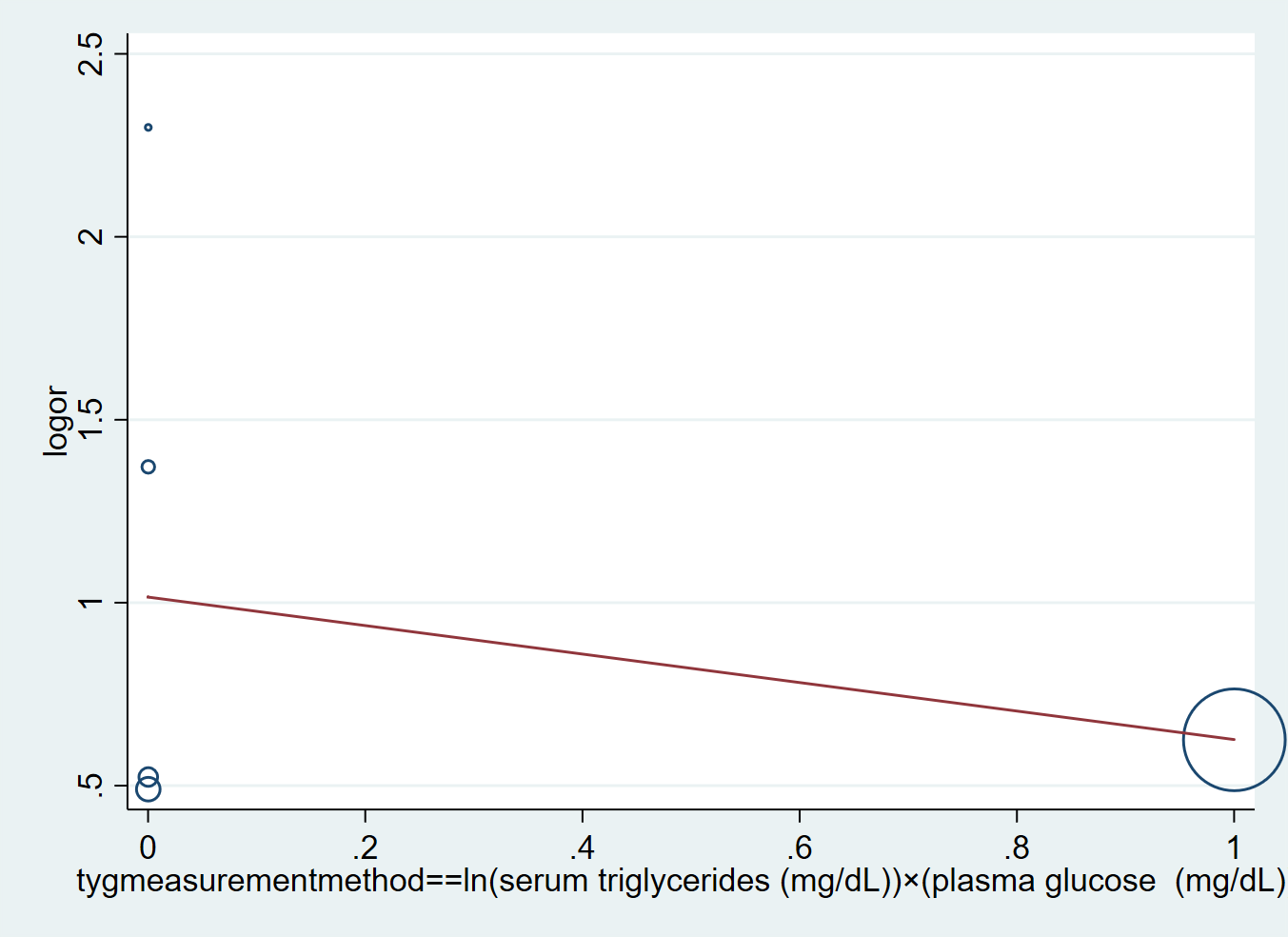


Figure S39 Meta-regression analysis based on the TyG measurement method(ln(serum triglycerides (mg/dL))×(plasma glucose (mg/dL))/2)


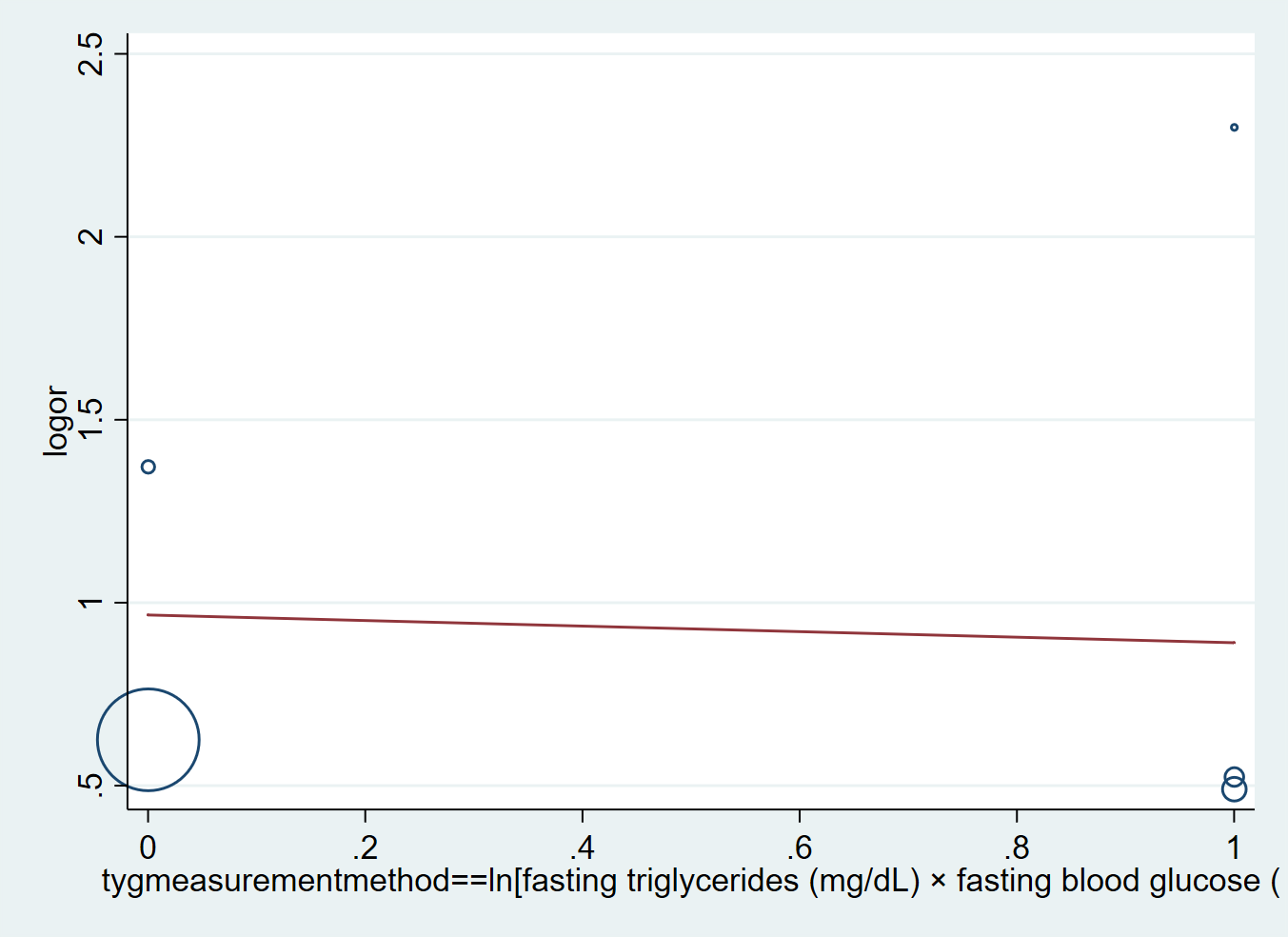


Figure S40 Meta-regression analysis based on the TyG measurement method(ln[fasting triglycerides (mg/dL) × fasting blood glucose (mg/dL)/2])
