# Supplementary figures and images for: Association between triglyceride-glucose index and sarcopenia: a meta-analysis
Source: PeerJ. 2026 Jul 23;14:e21424. doi: 10.7717/peerj.21424 (PMC13401845; doi:10.7717/peerj.21424)

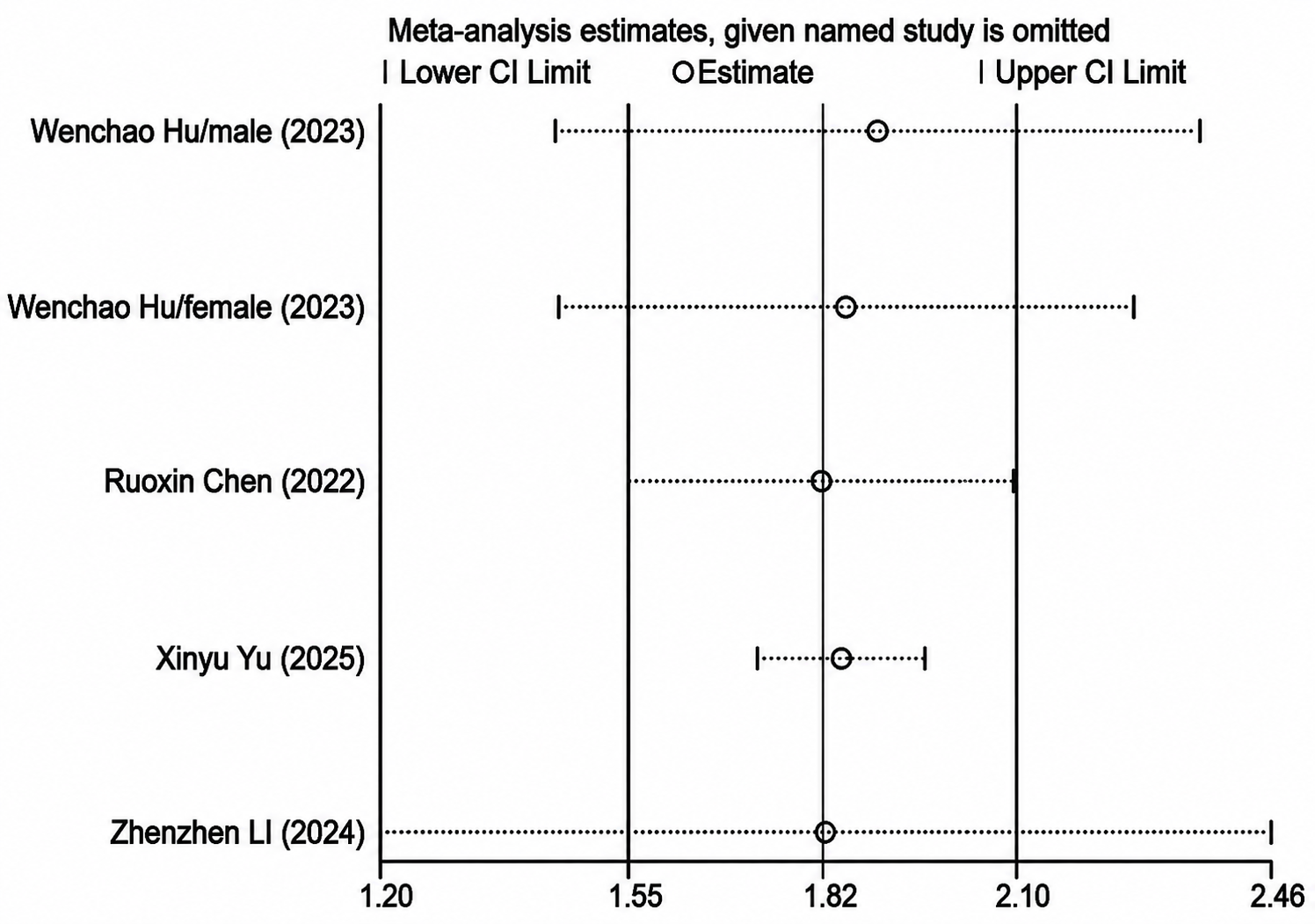

Supplement: Supplemental Information 2 [file peerj-14-21424-s002.png]

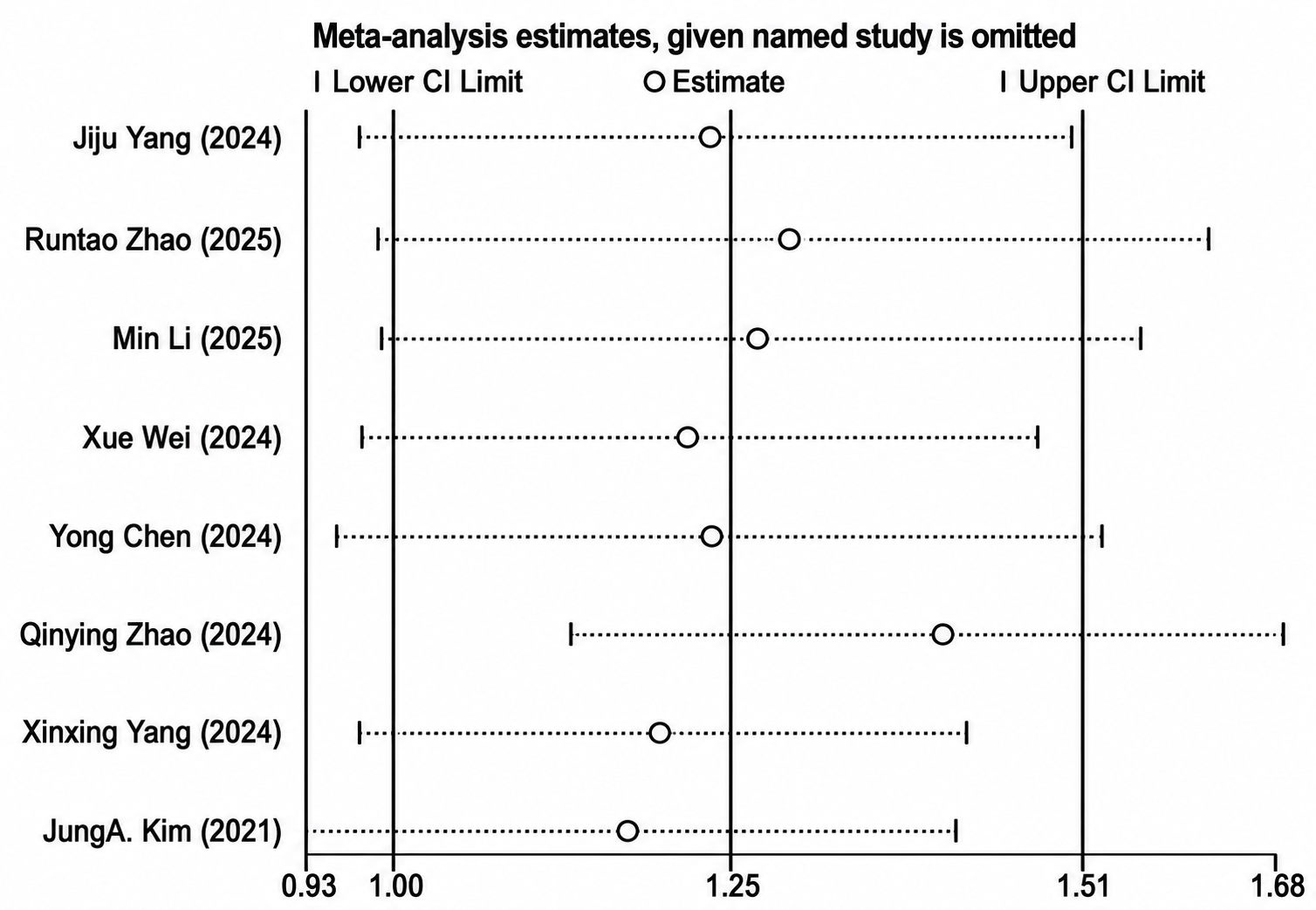

Supplement: Supplemental Information 3 [file peerj-14-21424-s003.png]

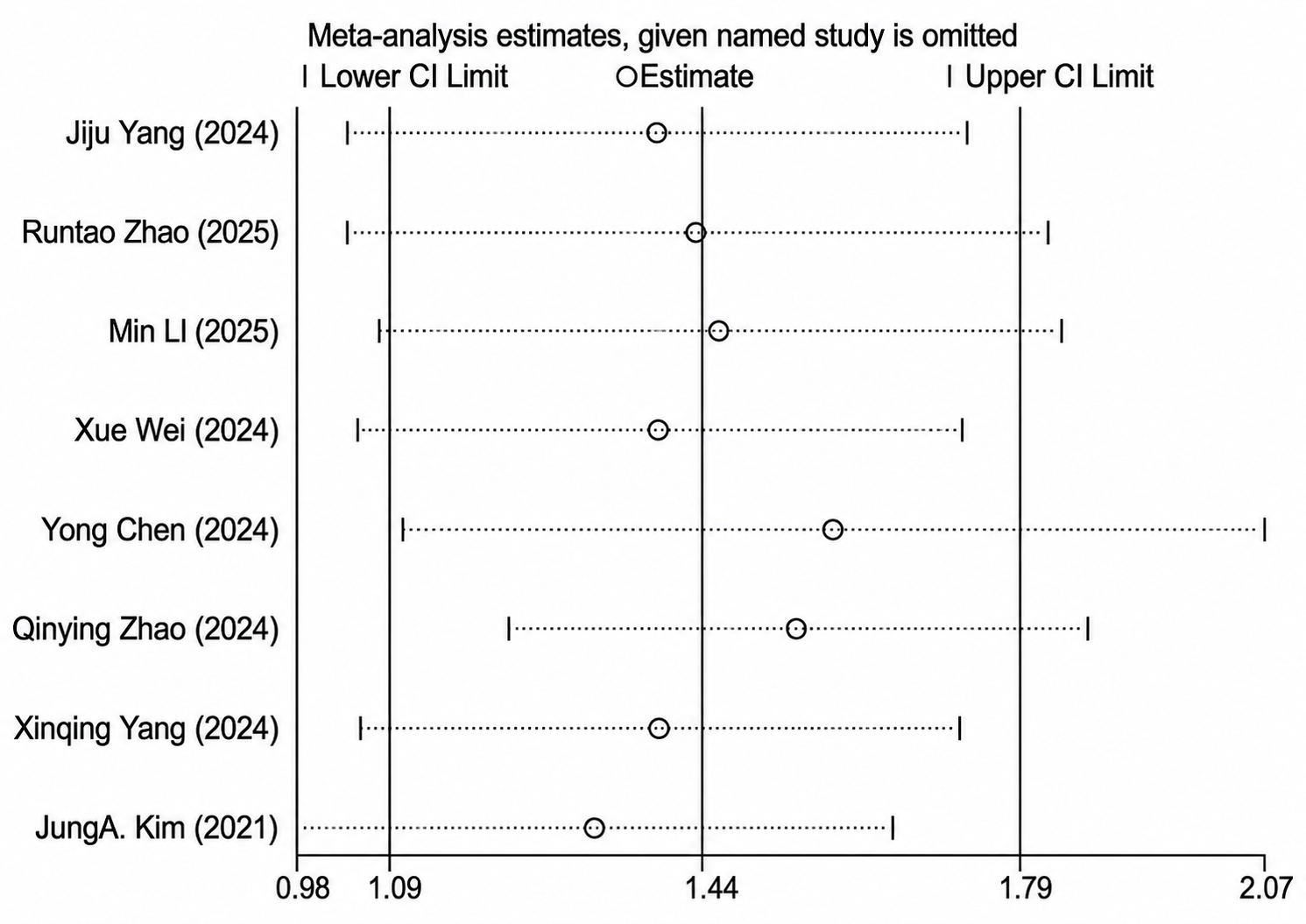

Supplement: Supplemental Information 4 [file peerj-14-21424-s004.png]

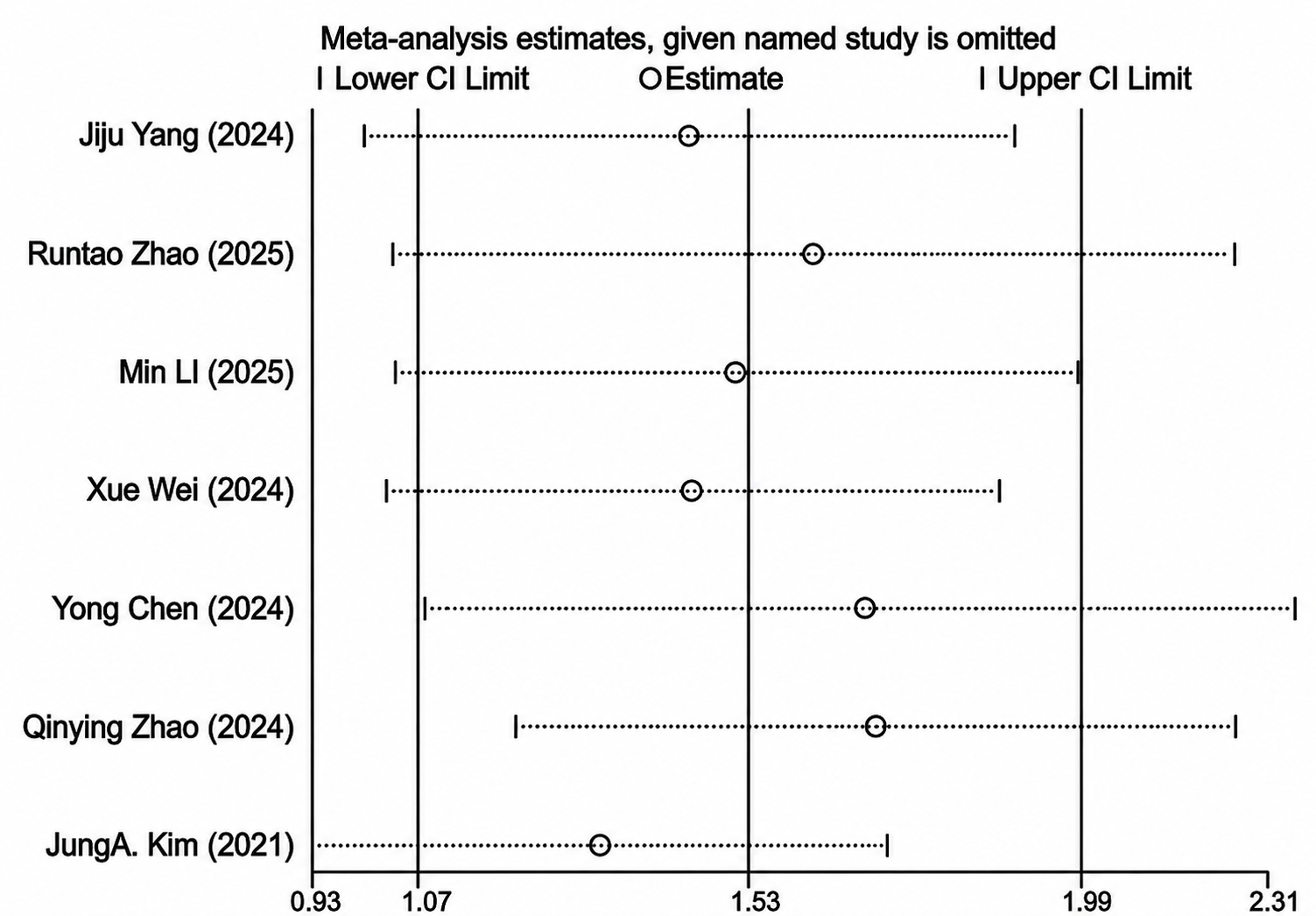

Supplement: Supplemental Information 5 [file peerj-14-21424-s005.png]

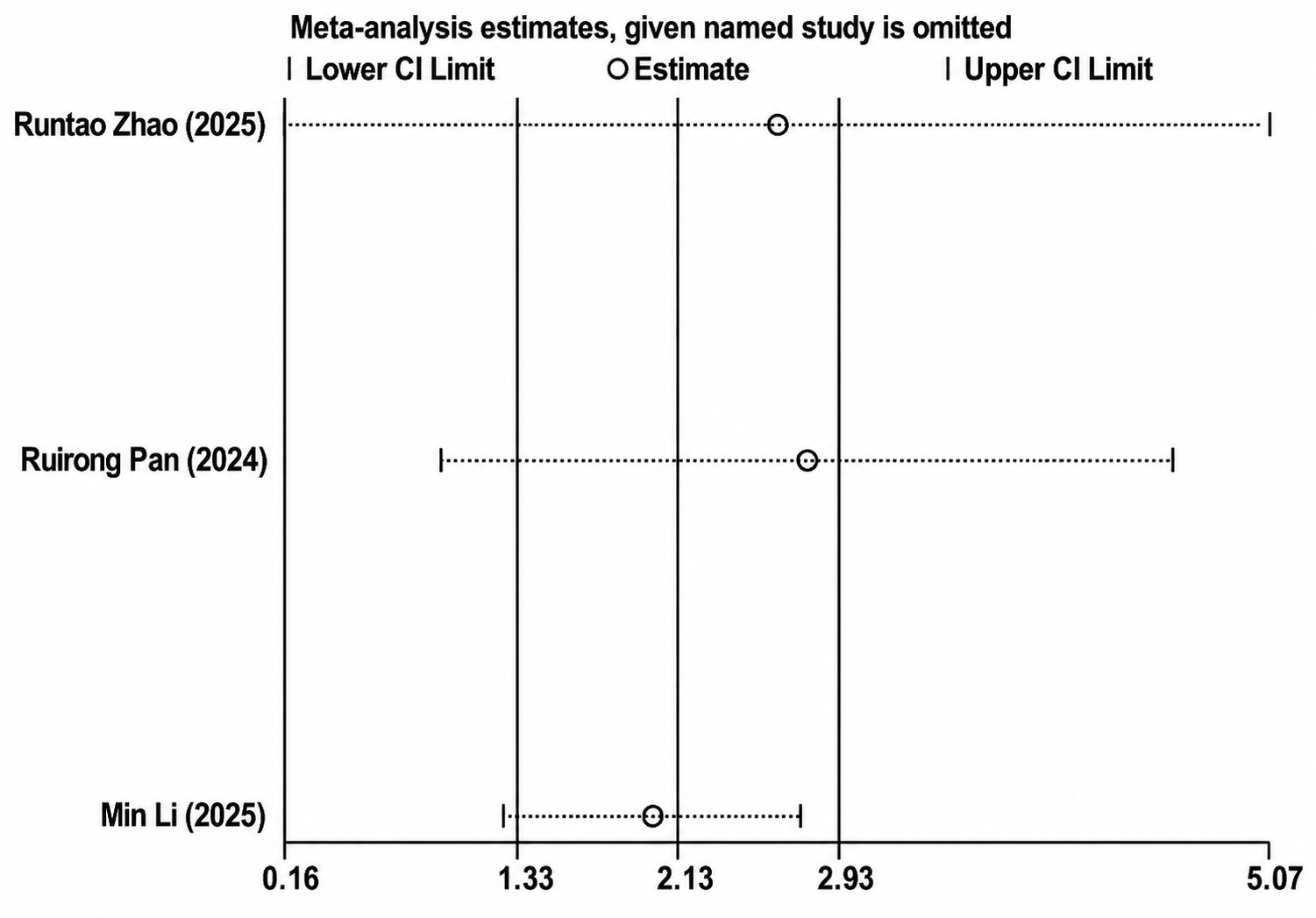

Supplement: Supplemental Information 6 [file peerj-14-21424-s006.png]

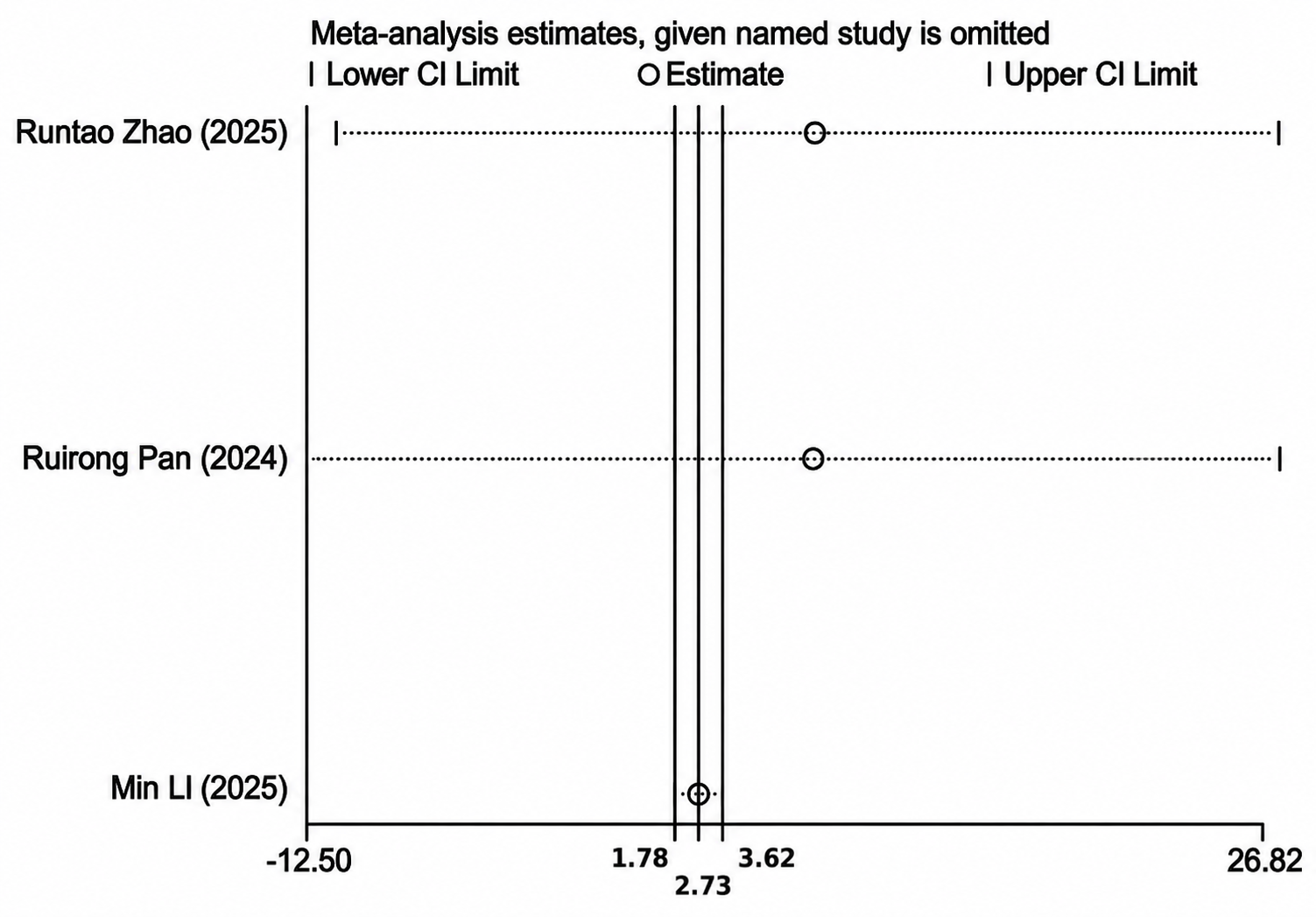

Supplement: Supplemental Information 7 [file peerj-14-21424-s007.png]

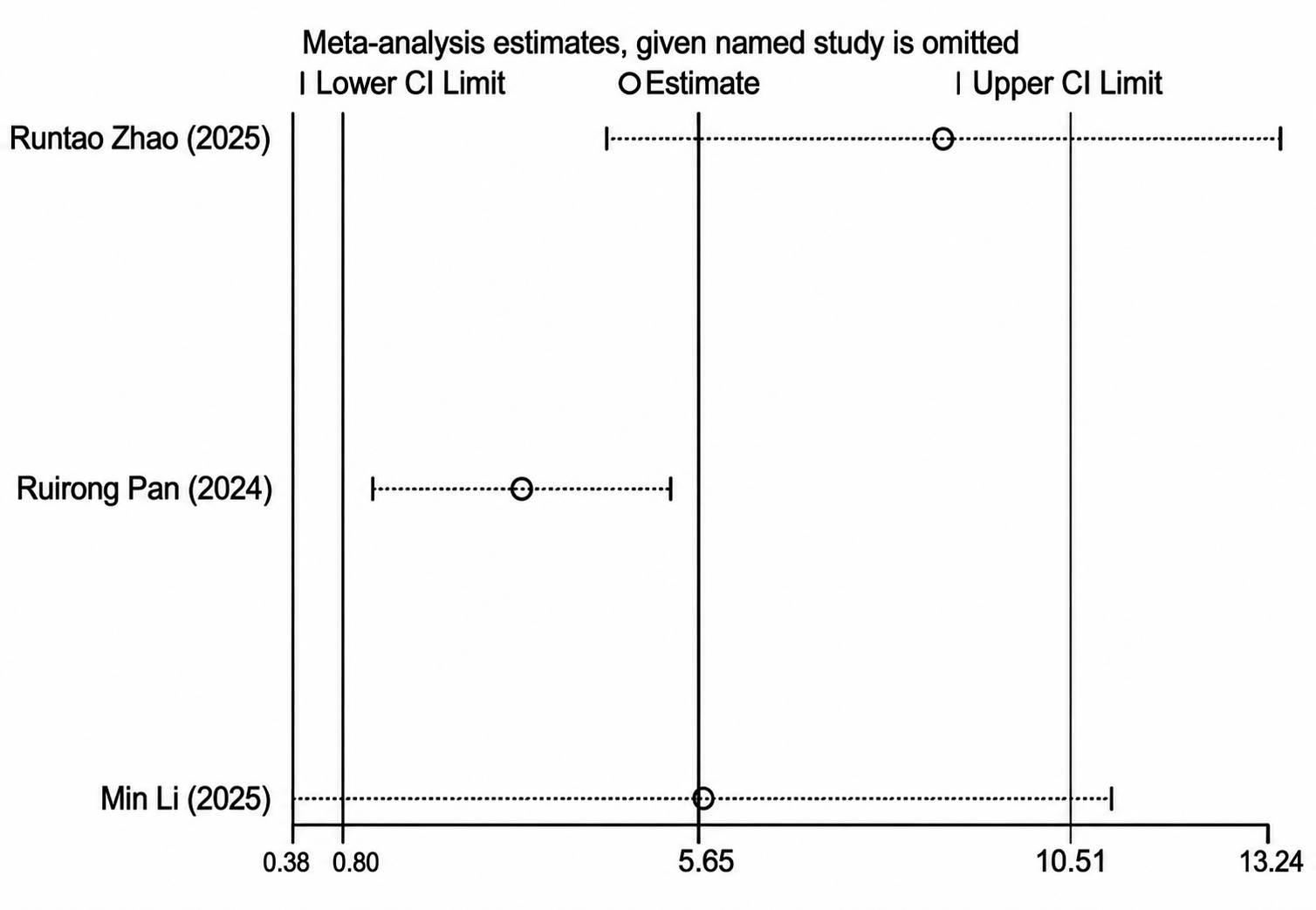

Supplement: Supplemental Information 8 [file peerj-14-21424-s008.png]

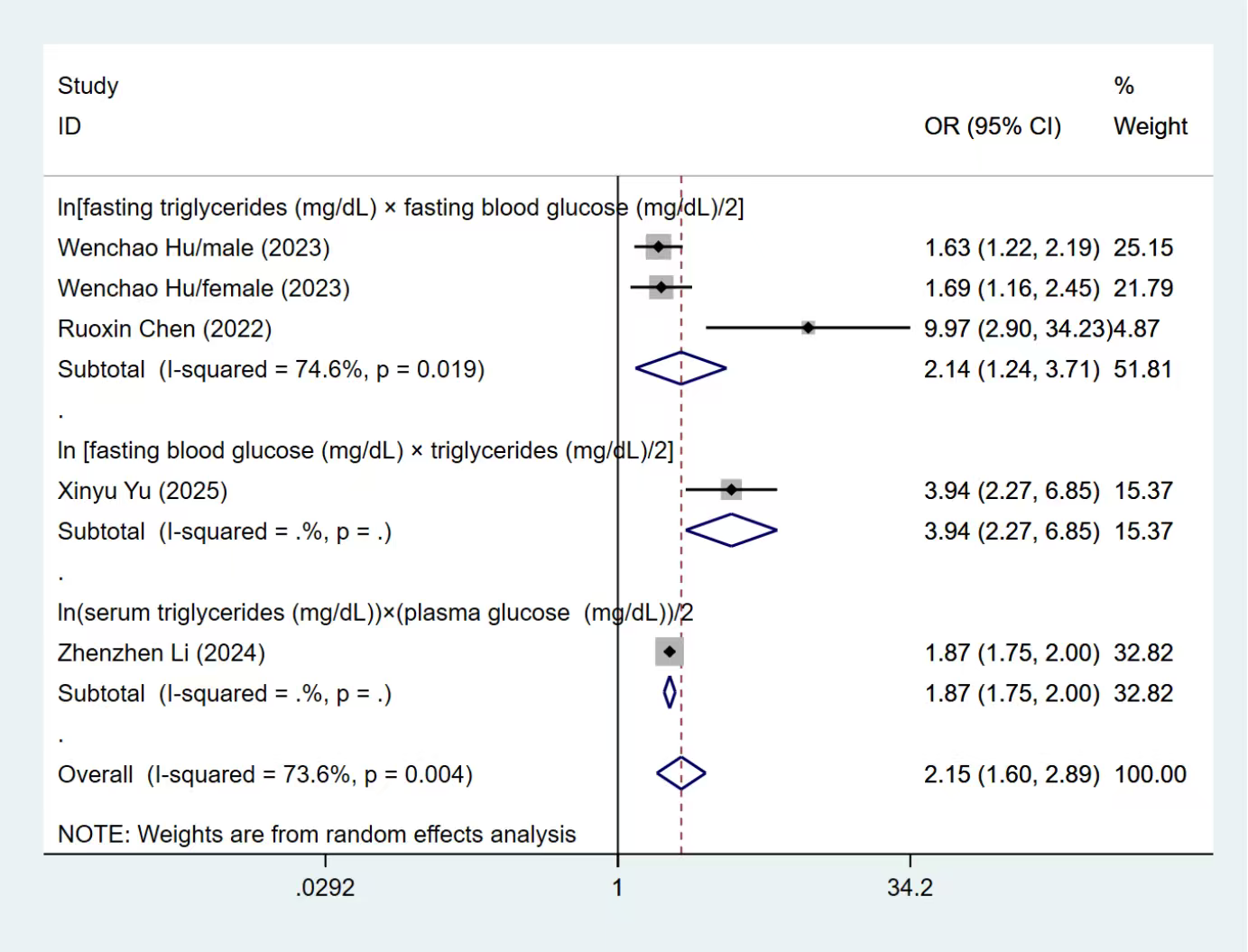

Supplement: Supplemental Information 9 [file peerj-14-21424-s009.png]

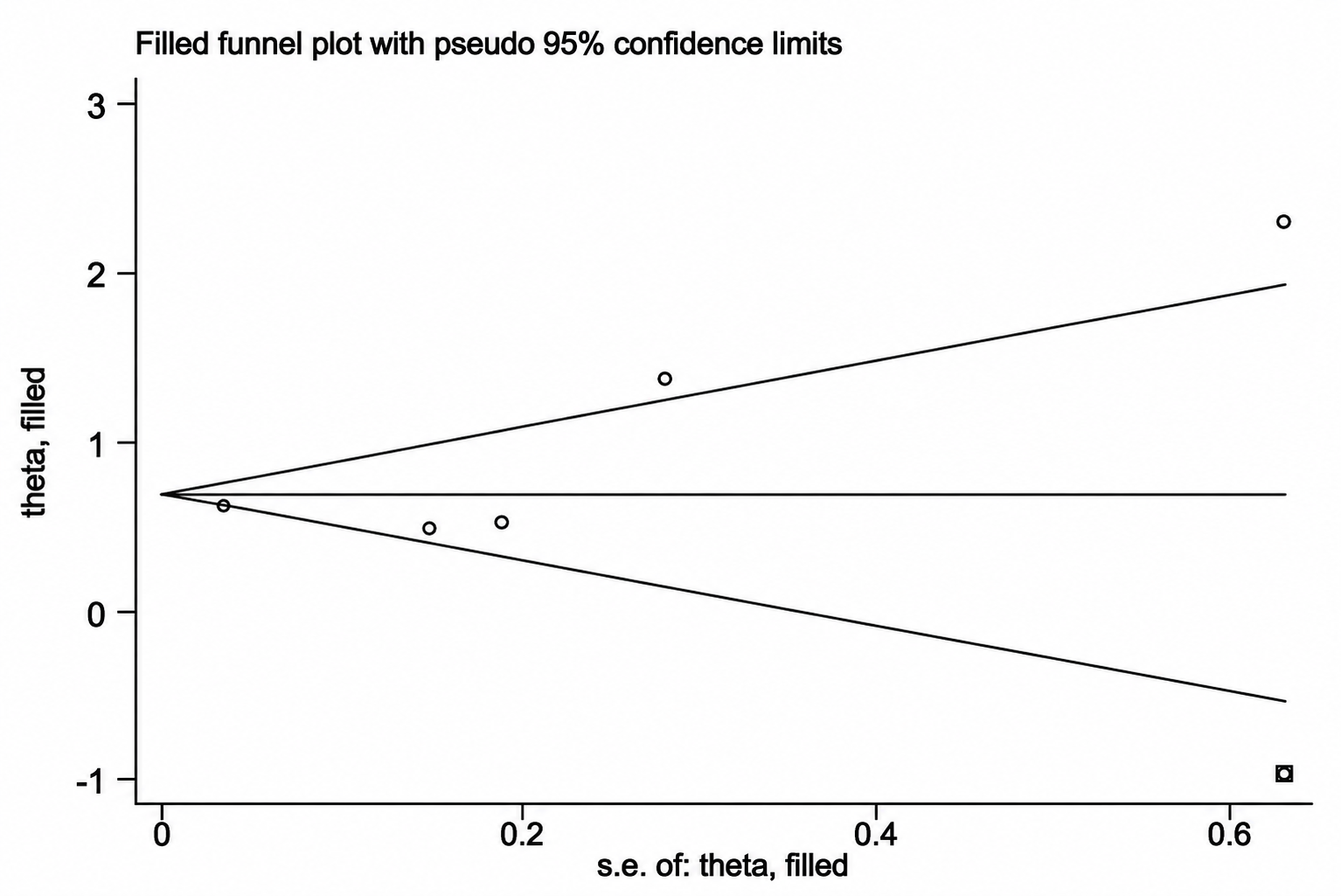

Supplement: Supplemental Information 10 [file peerj-14-21424-s010.png]

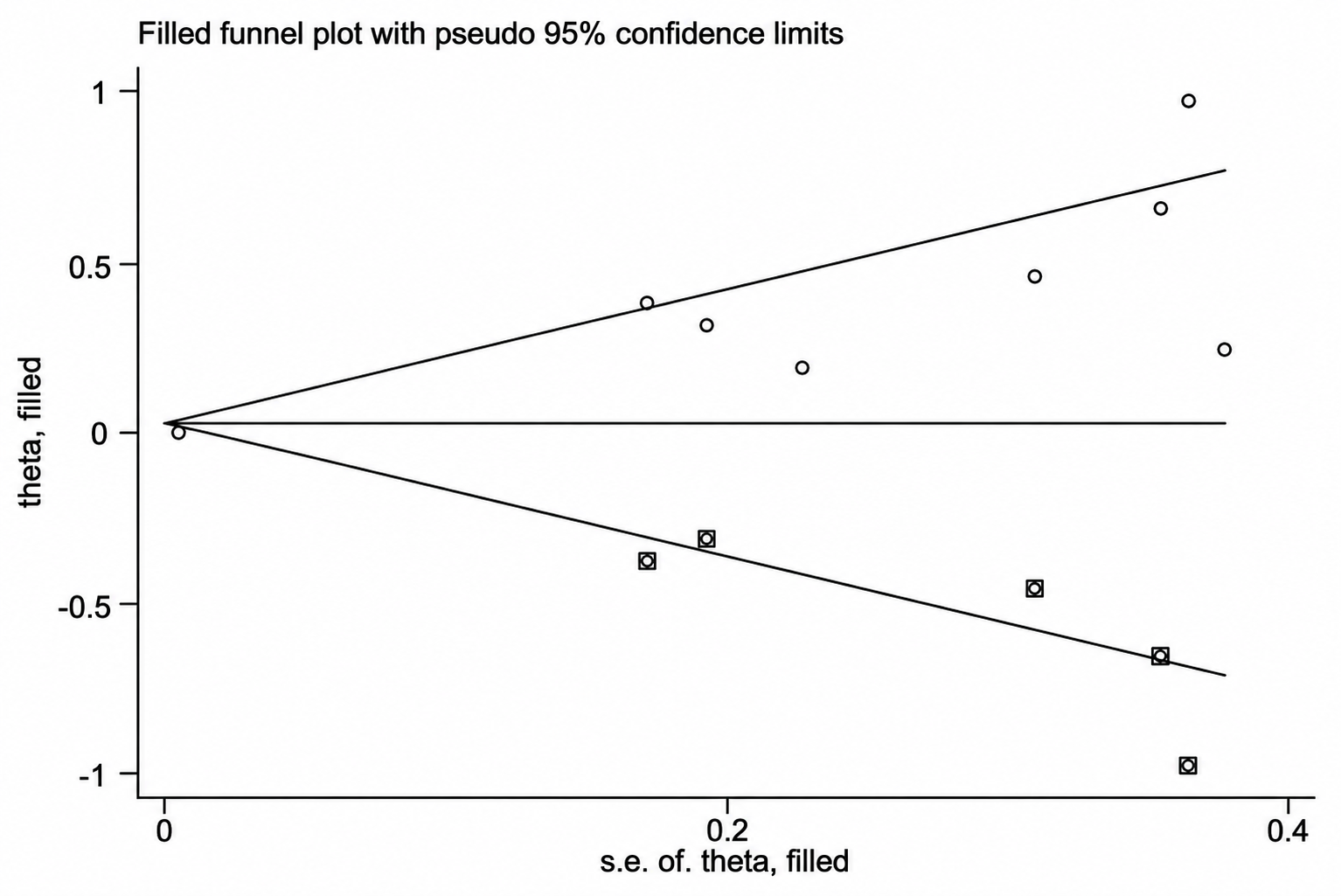

Supplement: Supplemental Information 11 [file peerj-14-21424-s011.png]

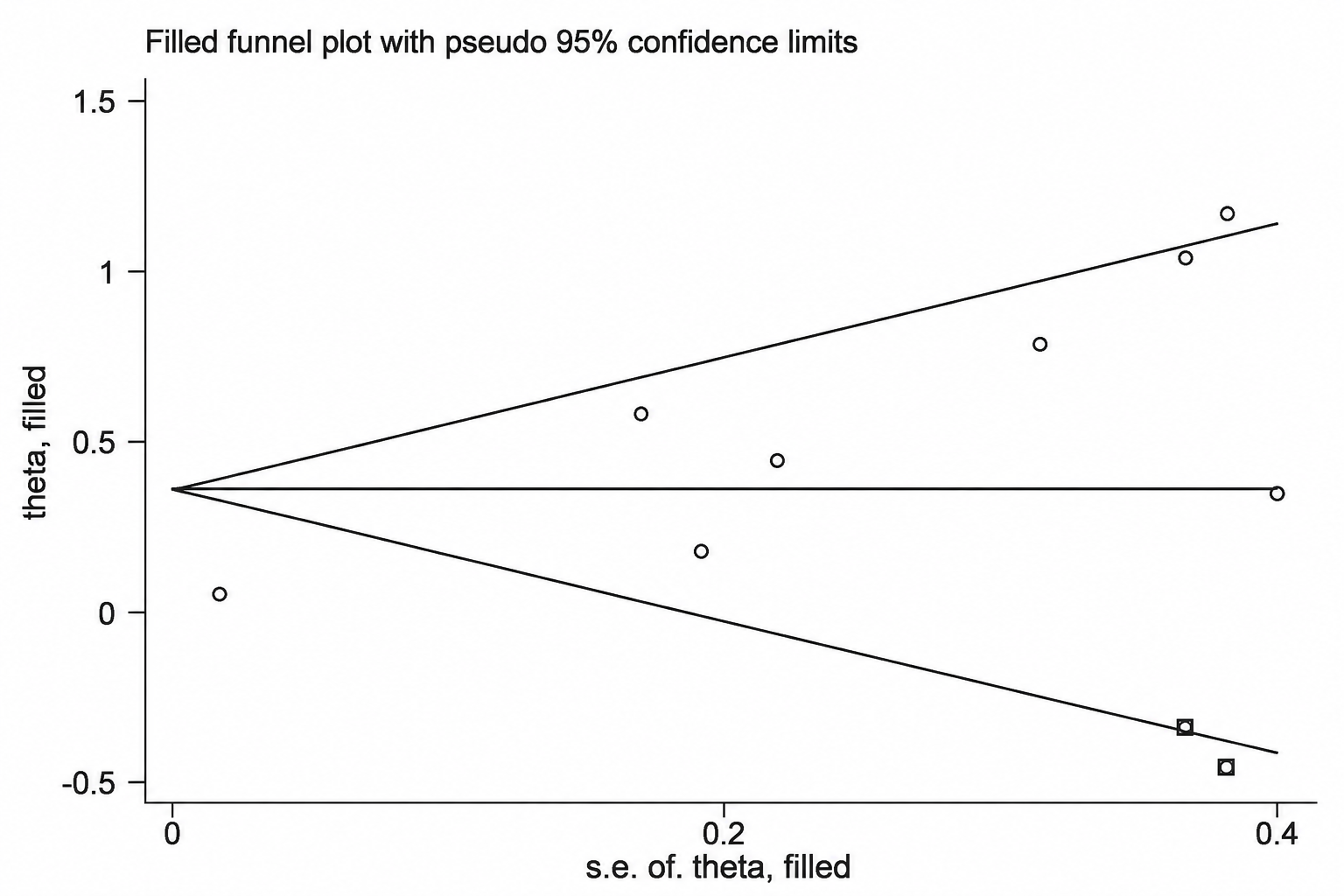

Supplement: Supplemental Information 12 [file peerj-14-21424-s012.png]

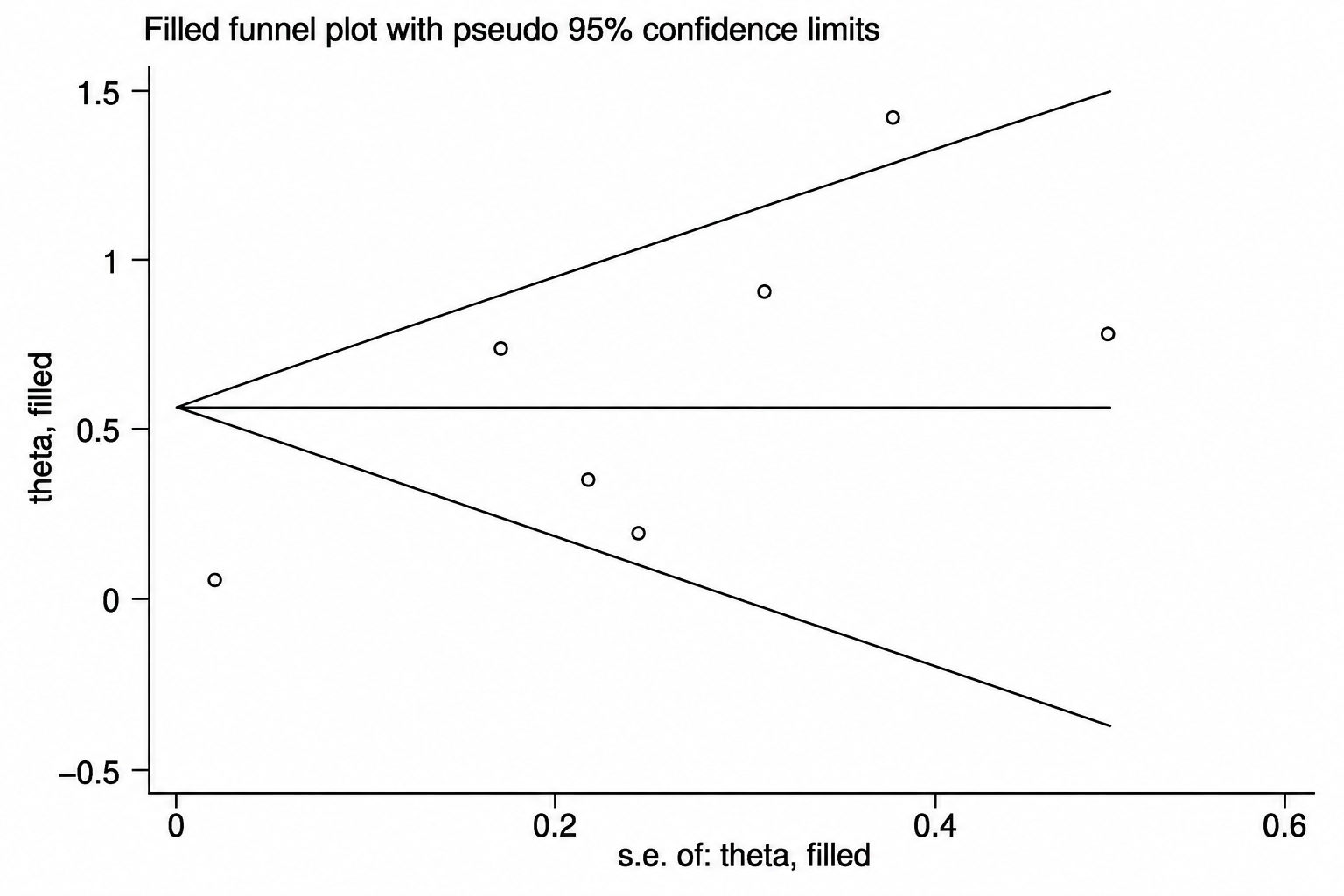

Supplement: Supplemental Information 13 [file peerj-14-21424-s013.png]

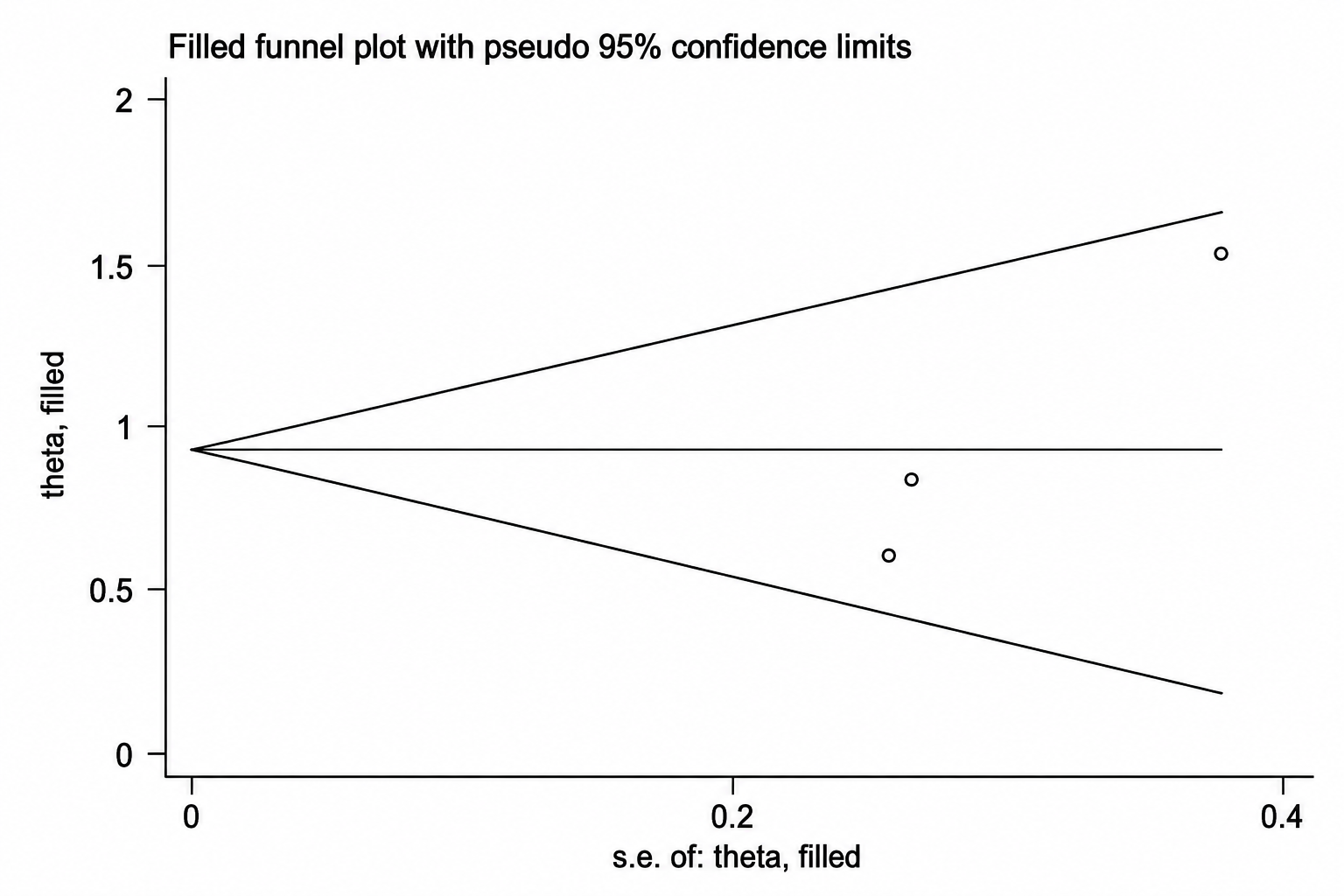

Supplement: Supplemental Information 14 [file peerj-14-21424-s014.png]

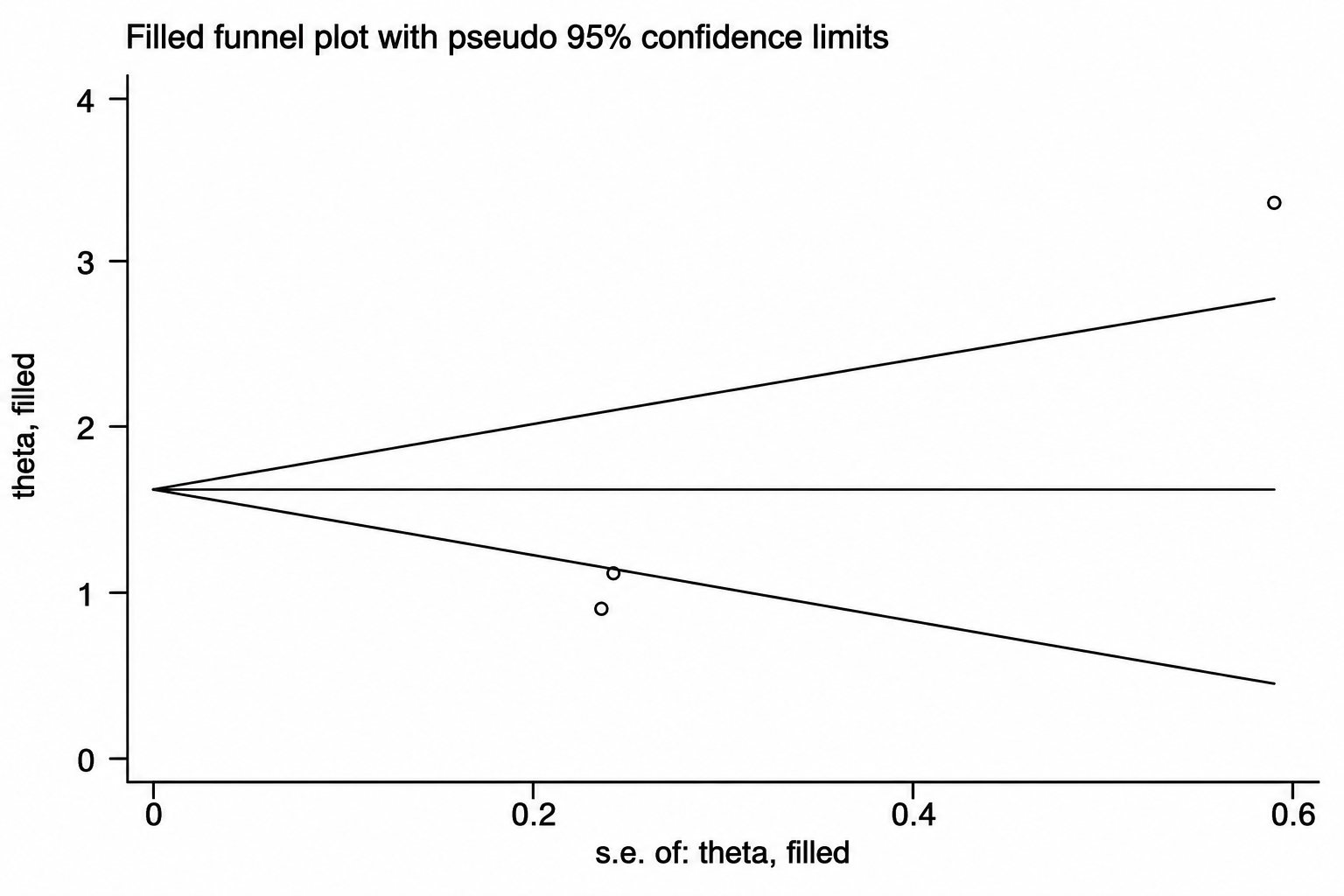

Supplement: Supplemental Information 15 [file peerj-14-21424-s015.png]

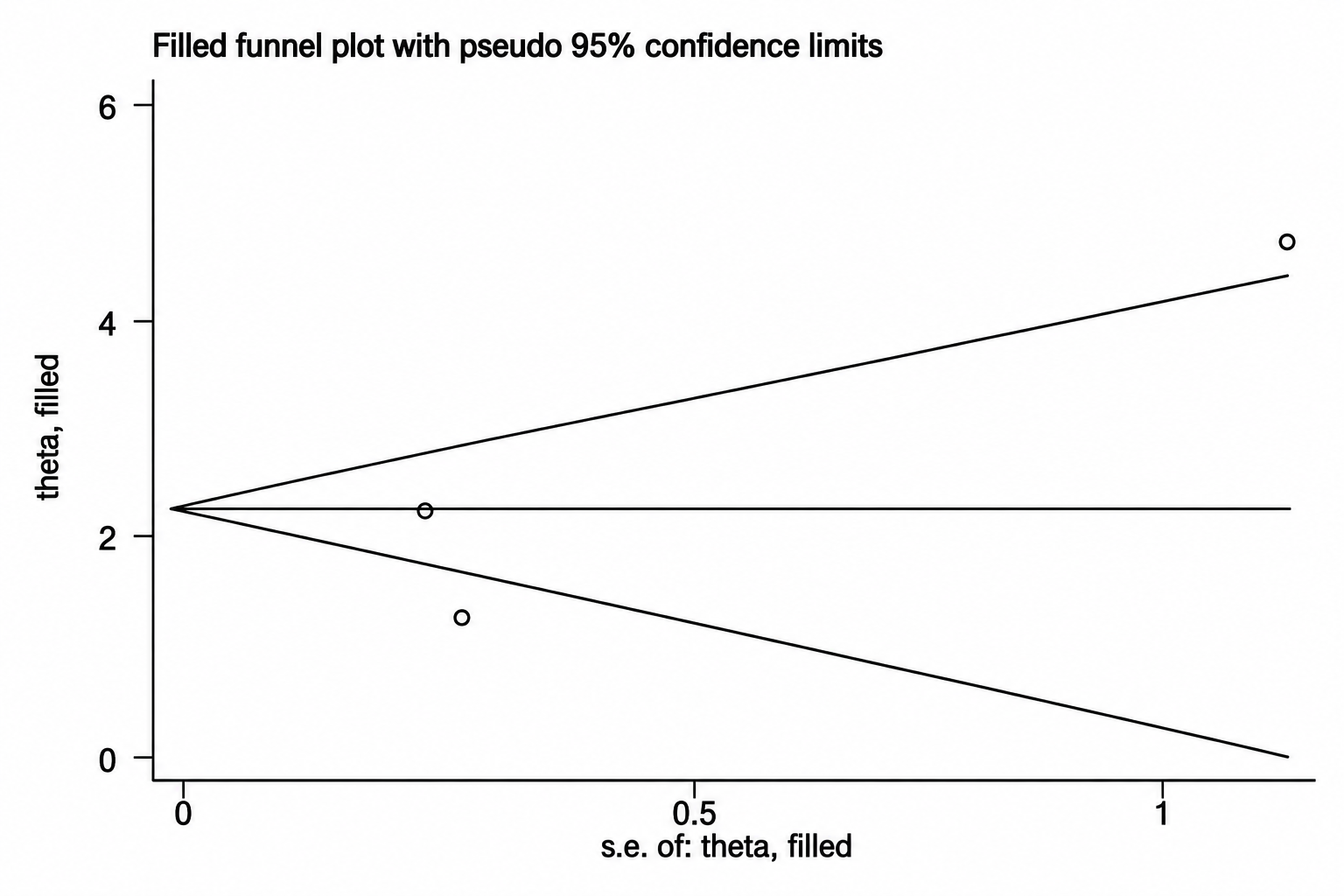

Supplement: Supplemental Information 16 [file peerj-14-21424-s016.png]
