# Supplementary material for: Association between triglyceride-glucose index and sarcopenia: a meta-analysis
Source: PeerJ. 2026 Jul 23;14:e21424. doi: 10.7717/peerj.21424 (PMC13401845; doi:10.7717/peerj.21424)
Supplement: Supplemental Information 17 [file peerj-14-21424-s017.docx]

| Section and Topic | Item # | Checklist item | Location where item is reported |
| --- | --- | --- | --- |
| TITLE | | |  |
| Title | 1 | Dose-Response Association Between Triglyceride-Glucose Index and Sarcopenia: A Meta-Analysis. | Lines1-2 |
| ABSTRACT | | |  |
| Abstract | 2 | Background: With the acceleration of population aging, the incidence of sarcopenia has significantly increased and is closely associated with the quality of life and mortality in the elderly. The triglyceride-glucose index has been shown to correlate with sarcopenia; however, a systematic review and meta-analysis providing a quantitative assessment of this relationship is still lacking.This study aims to investigate the association between the triglyceride-glucose index and sarcopenia by conducting a systematic review and meta-analysis.  Methods：A comprehensive and systematic search was conducted in the PubMed, Web of Science, Embase, and Cochrane Library databases for studies published from the inception of each database up to 15 June 2025 that examined the association between the the triglyceride-glucose index and sarcopenia. Data were analyzed using odds ratios (ORs) with 95% confidence intervals (CIs). All statistical analyses were performed using STATA version 15.0.  Results：This meta-analysis initially identified 122 publications, ultimately including 14 studies with a total sample size of 82,798 participants. The results indicated that the overall TyG (OR = 2.15, 95% CI: 1.60-2.89; P = 0.004), TyG-Q2 (OR = 1.38, 95% CI: 1.08-1.75; P = 0.003), TyG-Q3 (OR = 1.64, 95% CI: 1.21-2.20; P = 0.000), and TyG-Q4 (OR = 1.75, 95% CI: 1.21-2.55;P=0.000) were significantly associated with the risk of sarcopenia.Additionally, TyG-BMI-Q2(OR=2.51, 95% CI: 1.55-4.05;), TyG-BMI-Q3 (OR = 5.01, 95% CI: 1.89-13.25; P = 0.001), and TyG-BMI-Q4 (OR = 9.08, 95% CI: 2.91-28.37; P = 0.001) were significantly associated with an increased risk of sarcopenia. Subgroup analyses revealed that, according to the AWGS 2019 diagnostic criteria (OR = 5.28, 95% CI:2.27-12.3;), middle-aged individuals (OR = 1.87, 95% CI:1.46-2.42; P = 0.043), individuals with comorbidities (OR=1.94,95%CI:1.54-2.44;P= 0.044), and different TyG measurement methods (OR = 2.14, 95% CI: 1.24-3.71) were also significantly associated with the risk of sarcopenia.  Conclusions:This meta-analysis confirms a significant association between the TyG index and sarcopenia. As the TyG index increases, the risk of developing sarcopenia also rises, demonstrating a clear dose-response relationship between the two.  Trial registration: CRD420251067364.June 25, 2025  Keywords:Triglyceride-Glucose Index;Sarcopenia;Meta-analysis;Systematic review | Lines14-36 |
| INTRODUCTION | | |  |
| Rationale | 3 | Sarcopenia is a progressive musculoskeletal condition defined by a loss of muscle mass, strength, and physical performance. Following its onset, patients typically experience a marked reduction in muscular strength, ultimately leading to restricted mobility and significant deterioration in quality of life.Key adverse outcomes encompass heightened risks of fragility fractures, falls, injuries, disability, and reduced physical capacity. Moreover, the condition can threaten the independent living of older adults. Reductions in muscle mass and strength are also linked to worse outcomes from mechanical ventilation in hospitalized individuals and longer hospital admissions. Epidemiological data show that sarcopenia affects at least 4.6% of the older population, with prevalence rising to approximately 25% in elderly hospital inpatients. Muscle loss becomes especially pronounced in those aged 70 and older, with annual rates of muscle mass loss reaching 0.5% to 1.0% after the age of 70.Consequently, sarcopenia is now widely regarded as a major public health issue.The triglyceride-glucose (TyG) index is a recently established marker of metabolic function and a practical surrogate for insulin resistance. This index is calculated by combining the values of fasting plasma glucose and fasting triglyceride concentrations.Compared with the hyperinsulinemic-euglycemic clamp technique—the reference method for quantifying insulin resistance (IR)—the TyG index provides distinct practical benefits: it is inexpensive, easy to implement, and demonstrates high reproducibility. Its calculation relies solely on routine laboratory assays and a straightforward formula (7).In recent years, the TyG index has gained recognition as a robust predictor for incident metabolic diseases. Because of its strong correlation with insulin resistance, this index is useful for estimating the risk of several metabolic disorders, including metabolic syndrome, type 2 diabetes, non‑alcoholic fatty liver disease, cardiovascular diseases, and atherosclerosis (8).Sarcopenia is strongly linked to chronic low‑grade inflammation (CLIP). Older individuals with CLIP commonly exhibit reductions in both muscle mass and strength.In addition, inflammatory processes can worsen insulin resistance, which may consequently elevate the TyG index (9). A study suggests that proinflammatory mediators contribute importantly to tissue‑specific insulin resistance through disruption of insulin signaling pathways (10).Observational research has further established a direct connection between sarcopenia and insulin resistance, for which the TyG index represents a convenient proxy (11). A cross‑sectional investigation documented a significant positive association between the TyG index and skeletal muscle mass index among Korean adults (5). Additionally, a nationwide cohort study and Mendelian randomization analysis found a inverse relationship of the TyG index and both grip strength and walking speed. | Line36-70 |
| Objectives | 4 | This systematic review and meta-analysis aims to seeks to evaluate their relationship and generate quantitative evidence regarding the utility of the TyG index in predicting sarcopenia risk. | Lines71-73 |
| METHODS | | |  |
| Eligibility criteria | 5 | 1.Published within peer-reviewed journals;2. Observational studies, including case-control studies, cohort studies, and cross-sectional studies;3.Sarcopenia diagnosis in study subjects should be based on muscle mass assessment, in combination with muscle strength or physical function indicators, adhering to international definitions of sarcopenia ( AWGS 2019, AWGS 2014(2), FNIH(13), or EWGSOP 2010(14));4.Studies reporting the association between the TyG index or TyG-BMI index and sarcopenia;5.Outcomes must include the calculation of combined odds ratios and 95% confidence intervals, based on he TyG index stratified by quartiles (Q2: 2.90≤TyG<3.40; Q3: 3.40≤TyG<8.75; Q4:TyG≥8.75) and the TyG-BMI index stratified by quartiles (Q2:196≤TyG-BMI<236; Q3: 236≤ TyG-BMI<283;Q4:TyG-BMI≥283-571). | Lines103-114 |
| Information sources | 6 | Pumbed;web Of science ;embase;corhance library | Lines83-84 |
| Search strategy | 7 | ((sarcopenia[MeSH Terms]) OR ((sarcopenia[Title/Abstract]) OR (sarcopenias[Title/Abstract]))) AND (((((TyG index[Title/Abstract]) OR (triglyceride-glucose index[Title/Abstract])) OR ("triglyceride and glucose index"[Title/Abstract])) OR (triglyceride glucose index[Title/Abstract])) OR (triacylglycerol glucose index[Title/Abstract])) | Supplmentary materials |
| Selection process | 8 | Two researchers collaboratively developed the search strategy. Based on predefined inclusion and exclusion criteria, the retrieved literature was screened using EndNote X9 software. A comprehensive search strategy was designed across PubMed, Embase, Web of Science, and the Cochrane Library databases, up to June 15, 2025, using a combination of Medical Subject Headings (MeSH) and free-text terms.Key terms included "sarcopenia," "Triglyceride-Glucose Index," "sarcopenias," and "TyG index." Two researchers independently conducted an initial screening of titles and abstracts to assess potential relevance. Subsequently, two reviewers rigorously evaluated the eligibility of the articles. In cases of disagreement, a third researcher acted as an arbitrator to facilitate discussion and reach a consensus. | Lines79-100（The revisions are on pages 92-100) |
| Data collection process | 9 | The study screening and data collection were conducted independently by two evaluators. Any discrepancies were resolved through discussion within the research team. | Lines15-19 |
| Data items | 10a | first author, year of publication, country, sample size, participant age and sex, comorbidities, the diagnostic criteria for sarcopenia, and the statistical model employed. | Lines19-20 |
|  | 10b | definition of sarcopenia, and the statistical model used |  |
| Study risk of bias assessment | 11 | Funnel plots and Egger's test were also employed to detect publication bias. If funnel plots exhibited asymmetry, a trimming procedure was applied to further assess the robustness of the results. A p-value of <0.05 was considered statistically significant. | Lines139-140 |
| Effect measures | 12 | OR;95%CI | Lines132 |
| Synthesis methods | 13a | All data analyses in this study were performed using STATA 15.0 software for meta-analysis | Lines130 |
|  | 13b | A random-effects model was used when I² >50%, and a fixed-effects model was applied otherwise. | Lines133-134 |
|  | 13c | Sensitivity analysis was conducted using a stepwise exclusion method, recalculating the pooled effect size after sequentially excluding one study at a time. | Lines135-136 |
|  | 13d | Funnel plots and Egger's test were also employed to detect publication bias. | Lines137-138 |
|  | 13e | Heterogeneity among studies was assessed using the Q test and I² statistic. | Lines137-138 |
|  | 13f | Funnel plots and Egger's test were also employed to detect publication bias. | Lines137-138 |
| Reporting bias assessment | 14 | We assessed potential reporting bias using funnel plots and Egger’s test when ≥10 studies were included in a meta-analysis. For outcomes with insufficient studies, we descriptively compared registered protocols with published results when available. | Lines136-139 |
| Certainty assessment | 15 | was evaluated using the Grading of Recommendations, Assessment, Development, and Evaluation (GRADE) framework. |  |
| RESULTS | | |  |
| Study selection | 16a | A preliminary literature search identified 122 articles, which was reduced to 88 after duplicates were removed. Following a screening of titles and abstracts, 23 articles remained. After reviewing the full texts, 5 articles were excluded due to incomplete data. Of the remaining 18 articles, 2 were excluded because the data could not be extracted, and 2 others were excluded due to duplication in the original database. | Lines140-148 |
|  | 16b | Ultimately, 14 studies were included in the data analysis. | Lines140-148 |
| Study characteristics | 17 | involving a total of 82,798 participants. The mean age of the participants was 52.18 years, with males accounting for 52% of the sample. Geographically, all studies were conducted in Asia. Except for the study by Jung A. Kim et al.,which originated from South Korea, the remaining studies were from China. In terms of sarcopenia diagnostic criteria, eight studies adopted the AWGS criteria, one study used the 2010 EWGSOP criteria, and four studies employed non-international diagnostic standards. Additionally, one study used the FNIH diagnostic criteria. | Lines149-157 |
| Risk of bias in studies | 18 | The Egger’s testyielded a P-value <0.05, and the funnel plot showed right-skewed asymmetry, both suggesting the presence of publication bias in the included studies. | Lines173-174 |
| Results of individual studies | 19 | Of the 14 studies included, all were rated as either moderate or high quality, with an average AHRQ score of 6, reflecting good methodological quality. | Lines158-163 |
| Results of syntheses | 20a | Analysis using a random-effects model (I^²^ = 73.6%, P<0.01) showed that higher TyG levels were significantly associated with an increased risk of sarcopenia (OR = 2.15, 95% CI:1.60-2.89;P=0.004). | Lines165-174 |
|  | 20b | A random-effects model analysis (I^²^ = 67.2%, P < 0.01) indicated that higher TyG-Q2 levels were significantly associated with an increased risk of sarcopenia (OR = 1.38, 95% CI:1.08-1.75; P = 0.003) | Lines177-187 |
|  | 20c | The results of the meta-analysis (Figure 4) revealed a significant association between higher TyG-Q3 levels and an increased risk of sarcopenia (OR=1.64, 95%CI:1.21-2.20; P=0.000), as assessed using a random-effects model (I²=79.8%, P<0.01).esults. | Lines188-195 |
|  | 20d | higher TyG-Q4 levels were significantly associated with an increased risk of sarcopenia (OR=1.75,95%CI:1.21-2.55; P = 0.000). | Lines196-206 |
| Reporting biases | 21 | Sensitivity analysis and publication bias assessment suggest that, despite some bias in publication across studies, the association between the TyG index and sarcopenia remains stable and robust. | Lines303-305 |
| Certainty of evidence | 22 | Based on the GRADE assessment for the primary association (highest vs. lowest TyG index category and risk of sarcopenia), the certainty of evidence across all observational studies was rated as "low". The certainty for longitudinal cohort studies specifically was "moderate", while for cross-sectional studies it was "low". |  |
| DISCUSSION | | |  |
| Discussion | 23a | Summary of main results  This meta-analysis comprehensively included 14 eligible cross-sectional studies involving a total of 82,798 participants. The findings indicate that both the TyG index and the TyG-BMI index exhibit significant correlations with sarcopenia, with the likelihood of developing the condition rising notably as TyG index levels increase. This relationship was validated across multiple subgroup analyses, which were based on population characteristics, the presence of comorbidities, TyG measurement methods, and different sarcopenia diagnostic criteria. To our knowledge, the present work constitutes one of the earliest comprehensive meta-analyses to explore the potential link between sarcopenia and the TyG index. | Lines258-267 |
|  | 23b | **Mechanisms linking TyG index, insulin resistance and sarcopenia**  The decline in muscle mass associated with aging shows a strong connection with various adverse clinical outcomes, including reduced quality of life, longer hospitalization durations, elevated mortality, greater susceptibility to fractures and falls, poor responses to mechanical ventilation, and impaired cognitive function(29).Previous meta-analyses have mainly focused on the relationship between immune-inflammatory markers, malnutrition, resistance training interventions, protein supplementation,and the association between metabolic syndrome and sarcopenia(30-38). This meta-analysis, however, primarily consolidates evidence reveals a significant relationship between the TyG index—an indicator of insulin resistance—and sarcopenia.Given that skeletal muscle represents the major tissue responsible for glucose uptake, a decrease in muscle mass and strength leads to a considerable reduction in insulin sensitivity within skeletal muscle.According to a recent systematic review(39), patients with type 2 diabetes exhibit markedly reduced skeletal muscle insulin sensitivity, along with suppressed insulin-like growth factor 1 (IGF-1), which inhibits the PI3K/Akt/mTOR signaling pathway. Following a reduction in insulin sensitivity and impaired signaling pathways, glucose uptake rates decrease, leading to persistent hyperglycemia. At the same time, muscle protein synthesis is also suppressed.As individuals enter old age, the combined effects of aging, metabolic dysfunction, and chronic inflammation often result in a state of sustained low-grade inflammation. Pro-inflammatory mediators such as TNF-α, IL-6, and CRP activate cell surface signaling pathways including TNFR1/STAT3.This activation suppresses the Akt/mTOR pathway and consequently hinders protein synthesis, while simultaneously inducing autophagy and accelerating the degradation of proteins(40).This activation suppresses the Akt/mTOR pathway and consequently hinders protein synthesis, while simultaneously inducing autophagy and accelerating the degradation of proteins(40).Inflammatory cytokines, for instance C-reactive protein, exacerbate insulin resistance, which in turn adversely affects skeletal muscle protein metabolism(40).These mechanistic changes, both direct and indirect, compromise insulin sensitivity and disrupt normal IGF-1 and insulin signal transduction,ultimately leading to accelerated declines in muscle strength and mass.With the progression of insulin resistance, compromised glucose uptake and disrupted metabolic function exacerbate the adverse consequences of sarcopenia among middle-aged and elderly populations(40).As a result, excessive muscle loss is not solely a consequence of aging and obesity, but also a significant contributor to metabolic disorders and disease progression(41). | Lines268-358 |
|  | 23c | several limitations should be noted. First, given that all included studies are cross-sectional, the potential for reverse causality cannot be excluded. As a result, caution is warranted when inferring causal links between the TyG index and sarcopenia risk.Secondly, all cross-sectional studies incorporated in this review were conducted in Asian populations, lacking representation from other regions such as the Americas or Europe, which limits the generalizability of the findings. Thirdly, substantial heterogeneity was observed across the included studies,which can affect the stability and external validity of the pooled estimates. This heterogeneity is likely due to variations in study populations, comorbidities, and diagnostic criteria for sarcopenia. While subgroup analyses were conducted and a random-effects model was utilized to adjust for inter-study differences, persistent heterogeneity continues to be a limiting factor. | Lines375-385 |
|  | 23d | Consequently, the findings of this investigation warrant careful interpretation, and further rigorously designed, standardized prospective studies are required to more effectively elucidate the origins of the sources of heterogeneity. | Lines385-387 |
| OTHER INFORMATION | | |  |
| Registration and protocol | 24a | This systematic review and meta-analysis was conducted in strict adherence to the Preferred Reporting Items for Systematic Reviews and Meta-Analyses (PRISMA 2020) guidelines | Lines74-78 |
|  | 24b | the study protocol has been registered with the International Prospective Register of Systematic Reviews (PROSPERO), under the registration number CRD420251067364. | Lines74-78 |
|  | 24c | No changes. |  |
| Support | 25 | Not applicable. | Line414 |
| Competing interests | 26 | The authors declare that they have no competing interests. | Line413 |
| Availability of data, code and other materials | 27 | All data generated or analysed during this study are included in this published article [and its supplementary information files]. | Lines411-412 |

From: Page MJ, McKenzie JE, Bossuyt PM, Boutron I, Hoffmann TC, Mulrow CD, et al. The PRISMA 2020 statement: an updated guideline for reporting systematic reviews. BMJ 2021;372:n71. doi: 10.1136/bmj.n71. This work is licensed under CC BY 4.0. To view a copy of this license, visit <https://creativecommons.org/licenses/by/4.0/>
